# Awareness, treatment, and control of dyslipidemia in rural South Africa: The HAALSI (Health and Aging in Africa: A Longitudinal Study of an INDEPTH Community in South Africa) study

**DOI:** 10.1371/journal.pone.0187347

**Published:** 2017-10-27

**Authors:** Sheridan Reiger, Thiago Veiga Jardim, Shafika Abrahams-Gessel, Nigel J. Crowther, Alisha Wade, F. Xavier Gomez-Olive, Joshua Salomon, Stephen Tollman, Thomas A. Gaziano

**Affiliations:** 1 Department of Cardiovascular Medicine, Brigham & Women’s Hospital, Boston, MA, United States of America; 2 Center for Health Decision Science, Harvard T.H. Chan School of Public Health, Boston, MA, United States of America; 3 National Health Laboratory Service and Department of Chemical Pathology, Faculty of Health Sciences, University of the Witwatersrand, Johannesburg, South Africa; 4 Medical Research Council / University of the Witwatersrand Rural Public Health and Health Transitions Research Unit, School of Public Health, Faculty of Health Sciences, Johannesburg, South Africa; 5 Department of Global Health and Population, Harvard T.H. Chan School of Public Health, Boston, MA, United States of America; Mexican Social Security Institute, MEXICO

## Abstract

Dyslipidemia is a primary driver for chronic cardiovascular conditions and there is no comprehensive literature about its management in South Africa. The objective of this study was to assess the prevalence, awareness, treatment, and control of dyslipidemia in rural South Africa and how they are impacted by different behaviors and non-modifiable factors. To fulfill this objective we recruited for this cohort study adults aged ≥40 years residing in the Agincourt sub-district of Mpumalanga Province. Data collection included socioeconomic and clinical data, anthropometric measures, blood pressure (BP), HIV-status, point-of-care glucose and lipid levels. Framingham CVD Risk Score was ascribed to patients based upon categories for 10 year cardiovascular risk of low (<3%), moderate (≥3% and <15%), high (≥15% and <30%), and very high (≥30%).LDL cholesterol control by risk category was defined according to South African Guidelines. Multivariable logistic regression models were built to identify factors that were significantly associated with dyslipidemia and awareness of dyslipidemia From 5,059 respondents a total of 4247 subjects (83.9%) had their cholesterol levels measured and were included in our analysis. Overall, 67.3% (2860) of these met criteria for dyslipidemia, only 30 (1.05%) were aware of their condition, and only 21 subjects (0.73%) were on treatment. The majority have abnormalities in triglycerides (59.3%). As cardiovascular risk increased the rates of lipid control according to LDL level dropped. Multivariate logistic regression analyses showed that being overweight was predictive of dyslipidemia (OR 1.76; 95%CI 1.51–2.05, p<0.001) and dyslipidemia awareness (OR 2.58; 95%CI 1.19–5.58; p = 0.017). In conclusion, the very low awareness and treatment of dyslipidemia in this cohort indicate a greater need for systematic screening and education within the population and demonstrate that there are multiple opportunities to allay this burden.

## Introduction

As the epidemiologic transition continues to unfold, chronic cardiovascular conditions grow in their impact upon morbidity, health-related costs, and mortality in low resource countries[1, 2]. Dyslipidemia, defined as abnormalities of serum cholesterol or triglyceride levels which portend heightened risk of cardiovascular events including myocardial infarction and stroke, is a primary driver for these changes[3]. According to multiple global estimates, elevated total cholesterol remains a significant risk factor for overall disability and premature death[4]. Fortunately, dyslipidemia prevalence and impact have been stagnant or slowly decreasing in prevalence and impact over the last twenty to thirty years globally–including in Sub-Saharan Africa[5].

However, country specific studies in South Africa tell a more concerning story. A 2012 South African survey revealed that 28% of women and 19% of men older than 18 had elevated total cholesterol, and 52% and 44% of men and women respectively had low levels of HDL[6]. Higher total cholesterol is less common in the Black African population, though dyslipidemia overall is grossly similar to global averages[6]. Community level assessments have found prevalence rates of dyslipidemia between 14% and 69%[7–9].In South Africa, the treatment effects of HIV/AIDS complicate the picture of dyslipidemia[10]. This is particularly true given the well-known effect of protease inhibitors, a common component of antiretroviral therapy, which can elevate serum lipids by over 25% and accelerate progression towards cardiovascular events, but is also notable for first-line non-nucleoside and nucleoside reverse transcriptase inhibitors[11].

Unfortunately, there is nothing in the available literature about awareness, treatment, and control of dyslipidemia in South Africa. Though several reliable studies document prevalence rates of various lipid abnormalities, it is unknown how they are impacted by different behaviors and non-modifiable factors. These shortcomings in understanding what informs dyslipidemia prevalence, awareness, treatment, and control led to the development of the Cardiometabolic Disease in an Aging South African Cohort within the Health and Aging in Africa: Longitudinal Studies of an INDEPTH Community (HAALSI) Program. Specifically, this cohort study was initiated to discover the primary causes for changes in the prevalence, incidence, and risk factors of cardiometabolic disease in rural South Africa through the use of both self-reporting and direct assessment of disease and risks[12]. Baseline findings on the prevalence, awareness, treatment, and control of dyslipidemia in this cohort are reported. It is hoped that this will help elucidate the severity and impact of dyslipidemia in rural South Africa and provide guidance on potential avenues for control.

## Materials and methods

The HAALSI cohort is based in the Agincourt Health and Demographics Surveillance System (HDSS) site, a sub-district of rural Mpumalanga Province comprising approximately 116,000 people living in 21,000 households and 31 villages in an area of ~450km^2^. An annual census update, conducted by experienced local field staff, provides up-to-date denominator data on the full population with systematic recording of all vital events including deaths, births, and in-/out-migrations[13].

### Eligibility

All adults aged 40 years and older who had permanently resided in the Agincourt sub-district (Mpumalanga Province) for at least one year prior to the 2013 census update were eligible.

### Sampling and sample size

A total of 5,890 persons were identified for recruitment from the HDSS database using random sampling based on the 2013 Agincourt census data.

### Ethics approval

The study received ethical approvals from the Ethics Committees of three Institutions directly involved in the project: University of the Witwatersrand Human Research Ethics Committee (ref M141159), the Harvard T.H. Chan School of Public Health, Office of Human Research Administration (ref C13-1608-02) and the Mpumalanga Provincial Research and Ethics Committee (approved on 22nd October 2014).

### Recruitment and follow up

Prior to the survey, the HAALSI study was introduced to community members across the study villages, and discussed in-depth with a representative Community Advisory Group. This facilitated community review of study objectives and contributed to the effective response achieved.

Between November 2014 and November 2015 all identified individuals were visited at home by a supervised local field worker who described the study in the local language (Shangaan), and requested permission to read and explain the relevant informed consent forms. Those who agreed to participate signed a consent form or, if not able to sign their name, were asked to have a literate witness sign and date the informed consent on their behalf. Field workers also signed and dated the informed consent form. The first follow-up telephone call (to sustain cohort participation) was made to all participants six months after the interview and future follow-up calls will be made at six-month intervals moving forward.

### Data collection

Two to three hour interviews were conducted in participants’ households. The visit included a two part questionnaire: household and socioeconomic data then individual interview data; followed by physical and anthropometric assessments. Additionally, a random blood sample collection in the form of dried blood spots (DBS) was conducted. Blood sample collection was considered fasting if no caloric intake for at least 8 hours prior to the collection could be determined and that period was calculated as the reported difference between the time of the participant’s last meal and the time of the interview. Data were entered on laptop computers using a Computer Assisted Personal Interview (CAPI) program. Data collected included BP (OmronM6W automated cuff; Omron, Kyoto, Japan), weight (GenesisGrowth Management Electronic Scale; Johannesburg, South Africa); height using a height sensor with infrared measurement; waist and hip circumferences with a flexible tape measure (SECA, Hamburg, Germany). Blood drops were collected via finger prick on Whatman 903 paper(Whatman, Buckinghamshire, UK) and used to measure HIV status (1–3 drops), high-sensitivity C-reactive protein (hsCRP), glucose in point of care machines (Caresense N Monitor, Seoul, Korea) and individual lipid levels (Cardiocheck PA Silver version; Indianapolis, Indiana, USA).Three blood pressure readings (systolic and diastolic) were obtained with 2 minutes between each reading and the mean blood pressure was calculated using the average between the second and third reading[14].All self-reported conditions were captured using the question “Have you ever been told by a doctor, nurse, or other healthcare worker that you have (condition)?”. Self-reported current medication use for dyslipidemia and HIV (anti-retroviral) was also obtained during the interview.

### Definitions

Dyslipidemia was defined by participants meeting one or more of the following criteria: total cholesterol >5.0 mmol/L, LDL-cholesterol >3.0 mmol/L, HDL-cholesterol <1.2mmol/L, triglycerides >1.7 mmol/L, or self-reported treatment[6]. Framingham CVD Risk Score was ascribed to patients based upon categories for 10 year cardiovascular risk of low (<3%), moderate (≥3% and <15%), high (≥15% and <30%), and very high (≥30%)[15]. LDL cholesterol control by risk category was defined according to South African Consensus Guidelines of LDL <3mmol/L for Framingham low/moderate risk, LDL <2.5mmol/L for high risk, and LDL <1.8mmol/L for very high risk[16].

Clinical determination of HIV status was determined by first using the Vironostika Uniform 11 (Biomeriuex, France) screening assay. Negative results were assigned a HIV-negative status, while positive results triggered a second (confirmatory) test using the Roche Elecys (USA) assay to determine the viral load. If both screening and confirmatory tests were positive, a final status of HIV-positive was assigned and the viral load subsequently calculated. If the screening and confirmatory tests yielded opposing results, a third assay was run on the Siemens Centaur XP (USA) immunoassay. This third test served as the tie-breaker to determine a final HIV-positive or negative status, in accordance with World Health Organization (WHO) guidelines[17, 18]. HIV-positive status was defined as a self-reported history of being informed of the condition by a health professional or positive result on assay analysis.

Age stratification followed the WHO definition for elderly in developing countries (≥60 years)[19] while Body Mass Index (BMI) in kg/m^2^ was used to categorize subjects as overweight (BMI ≥25kg/m^2^) using World Health Organization (WHO) cutoffs[20]. Socioeconomic status (SES) is a composite, constructed variable incorporating measures of traditional and modern wealth captured in the computerized questionnaire, using methodology created for Demographic and Health Surveys(DHS) and divided into 5 quintiles–higher SES included 4th and 5th higher quintiles[21, 22]. Immigrants were defined as the subjects who were born outside South Africa. Cardiovascular disease was defined by self-report of stroke, myocardial infarction, angina, or a diagnosis of angina by Rose criteria (World Health Organization Rose questionnaire is widely used in epidemiological studies and is a validated and standardised method for defining angina pectoris)[23]. Smoking status was defined by self-reported current smoking status. Awareness captured persons with a self-reported history of being informed of the condition by a health professional. The subset of persons reporting medication use among those aware of their condition was defined as those being treated. Finally, those deemed as having control of their condition was defined as the subset of persons who met target levels and who are also taking medication for the given condition, including dyslipidemia.

### Analyses

All analyses were conducted using STATA ® V14 software. Continuous variables were compared using T test (expressed in mean values and standard deviations, since they were normally distributed) and categorical variables using Chi Square test (expressed in absolute numbers and percentiles). Multivariable logistic regression models were built to identify factors that were significantly associated with dyslipidemia and awareness of dyslipidemia. The variables included in the two models were the same (gender, HIV diagnosis, age, immigration status, social economic status, cardiovascular disease and being overweight or obese) and were previously assessed in this population[24]. The results are presented using odds ratios and 95% confidence intervals. The level of significance was set at 5%.

## Results

From the full sample of 5,890 persons who were alive and residing in the area at the time of recruitment 5059 (85.9%) agreed to participate, 430 refused (7.3%), 353 (6.0%) were not located, and 48 (0.8%) were unable to participate. A total of 4247 subjects (83.9% from the participants) had their cholesterol levels measured and therefore were included in this analysis. The mean age of the population was 61.9±12.9 years with the dyslipidemic subjects being roughly the same age (61.8±12.6 years). The mean BMI was 27.4±6.96 kg/m^2^ and was slightly higher in the dyslipidemic population (28.1±6.68 kg/m^2^) compared to non-dyslipidemic subjects (25.9±7.28 kg/m^2^). Additionally, the dyslipidemic subjects had a larger mean waist circumference and waist-to-hip ratio, had lower smoking rates and higher blood pressure and glucose levels than non-dyslipidemic patients. Regarding the effect of HIV positivity and treatment there was no statistical difference in dyslipidemia rates for subjects that were HIV positive, and HIV treatment was associated with lower rates of dyslipidemia. Clinical and laboratory characteristics of these participants are summarized in Table 1.

**Table 1:** Overall, non-dyslipidemic and dyslipidemic population characteristics, Agincourt sub-district, South Africa 2015.

Factor	Overall	Non-dyslipidemic	Dyslipidemic	p-value*
***n***	4247	1387	2860	
**Male sex**	1917 (45.1%)	666 (48.0%)	1251 (43.7%)	0.009
**Age (years)**	61.87 (±12.89)	61.95 (±13.36)	61.83 (±12.65)	0.770
**Weight (kg)**	72.08 (±17.68)	67.94 (±16.34)	74.10 (±17.95)	<0.001
**Height (m)**	1.63 (±0.09)	1.63 (±0.09)	1.63 (±0.09)	0.860
**Body mass index (kg/m**^**2**^**)**	27.36 (±6.96)	25.92 (±7.28)	28.06 (±6.68)	<0.001
**Waist circumference (cm)**	92.76 (±15.21)	88.77 (±14.10)	94.72 (±15.35)	<0.001
**Waist/hip ratio**	0.91 (±0.08)	0.89 (±0.08)	0.91 (±0.08)	<0.001
**Systolic blood pressure (mmHg)**	138.12 (±23.27)	136.17 (±22.80)	139.06 (±23.44)	<0.001
**Diastolic blood pressure (mmHg)**	82.13 (±12.62)	81.00 (±12.10)	82.68 (±12.83)	<0.001
**Glucose (mmol/L)**	6.68 (±3.20)	6.17 (±2.11)	6.93 (±3.59)	<0.001
**Total cholesterol (mmol/L)**	4.24 (±1.26)	3.63 (±0.80)	4.53 (±1.34)	<0.001
**HDL-cholesterol (mmol/L)**	1.57 (±0.55)	1.74 (±0.39)	1.49 (±0.59)	<0.001
**Triglyceride (mmol/L)**	1.76 (±1.57)	1.14 (±0.30)	2.06 (±1.83)	<0.001
**LDL-cholesterol (mmol/L)**	2.12 (±1.50)	1.67 (±0.62)	2.37 (±1.77)	<0.001
**High-sensitivity CRP (mg/L)**	3.28 (±3.02)	3.13 (±3.01)	3.35 (±3.03)	0.033
**Smoker**	379 (8.9%)	159 (11.5%)	220 (7.7%)	<0.001
**HIV positive**	1004 (23.7%)	338 (24.4%)	666 (23.3%)	0.440
**HIV on treatment**	490 (11.5%)	184 (13.3%)	306 (10.7%)	0.014
**HIV without treatment**	514 (12.1%)	154 (11.1%)	360 (12.6%)	0.160

Dyslipidemia defined as total cholesterol > 5 mmol/L ***or*** low-density lipoprotein (LDL) > 3.0 mmol/L ***or*** high-density lipoprotein (HDL) < 1.2 mmol/L ***or*** Triglycerides > 1.7 mmol/L ***or*** Self-reported treatment. Data given as mean ± SD or n (%)

*p-value for comparison of dyslipidemic vs. non-dyslipidemic subjects

Overall, 67.3% (2860) of subjects met criteria for dyslipidemia, only 30 (1.05%) of those with dyslipidemia were aware of their condition, and only 21 subjects (0.73%) were on treatment. The majority of subjects with dyslipidemia had abnormalities in measured triglycerides (59.3%), with all other lipid irregularities ranging from 36.7 to 40.1% prevalence–summarized in Table 2. This translated to a population prevalence of elevated total cholesterol of 25.4%, elevated LDL of 24.7%, low HDL of 27.0%, and elevated triglycerides of 39.9%. The majority of reported treatments consisted of diet and weight loss (17/21), with only 4 subjects on allopathic medications. Herbal medicines were being taken by 9 of the 21 subjects under treatment. We assessed the relationship between HDL and triglycerides using a Pearson Coefficient and found that there was a statistically significant (p = 0.003) and negative (r = -0.094) relationship between HDL and triglycerides.

**Table 2:** Prevalence of dyslipidemia by different definitions in the HAALSI dyslipidemic population (n = 2860).

Dyslipidemia category	n (%)
**Total cholesterol > 5 mmol/L**	1077 (37.66)
**low-density lipoprotein (LDL) > 3.0 mmol/L**	1050 (36.71)
**high-density lipoprotein (HDL) < 1.2 mmol/L**	1147 (40.10)
**Triglycerides > 1.7 mmol/L**	1696 (59.30)
**Self-reported treatment**	21 (0.73)

Dyslipidemia defined as total cholesterol > 5 mmol/L ***or*** low-density lipoprotein (LDL) > 3.0 mmol/L ***or*** high-density lipoprotein (HDL) < 1.2 mmol/L ***or*** Triglycerides > 1.7 mmol/L ***or*** Self-reported treatment

When dyslipidemic subjects were categorized by Framingham Cardiovascular risk it was found that half of subjects were low risk, with 11.3% meeting criteria for high or very high risk (Table 3).

**Table 3:** Risk Category according to Framingham Risk Score among the HAALSI dyslipidemic population (n = 2,790).

Risk Category	n	%
**Low**^**1**^	1,399	50.14
**Moderate**^**2**^	1,076	38.57
**High**^**3**^	235	8.42
**Very High**^**4**^	80	2.87

(1) Risk of cardiovascular events < 3% in 10 years.

(2) Risk of cardiovascular events > = 3% and < 15% in 10 years.

(3) Risk of cardiovascular events > = 15 and < 30% in 10 years.

(4) Risk of cardiovascular events > = 30% in 10 years.

Additionally, as calculated cardiovascular risk increased for dyslipidemic subjects the proportion of subjects with controlled LDL decreased (see Table 4).

**Table 4:** Subjects with LDL-cholesterol under control according to Risk Category among the HAALSI dyslipidemic population (n = 2,790).

Risk Category	LDL-cholesterol controlled	
	n	%
**Low (n = 1,399)**	897	64.1
**Moderate (n = 1,076)**	682	63.4
**High (n = 235)**	123	52.3
**Very High (n = 80)**	9	11.3

LDL-cholesterol under control—Low risk and moderate risk–LDL cholesterol <3mmol/L; high risk–LDL cholesterol < 2.5mmol/L; very high risk–LDL cholesterol <1.8mmol/L.

According to the multivariate logistic regression analyses overweight or obesity was positively associated with both dyslipidemia and dyslipidemia awareness, while gender, higher SES bracket, HIV status, age ≥60 years old, being an immigrant and presence of co-morbid cardiovascular disease were not (Tables 5 and 6).

**Table 5:** Variables independently associated to dyslipidemia^1^ in the HAALSI population.

Variable	Dyslipidemia	
	OR (95% CI)	*p*
**Gender (Female)**	1.05 (0.92–1.20)	0.492
**HIV negative**^**2**^	0.98 (0.84–1.15)	0.853
**Elderly**^**3**^	1.04 (0.91–1.20)	0.497
**Immigrant**	0.96 (0.83–1.11)	0.567
**SES**^**4**^ **(higher)**	1.13 (0.99–1.31)	0.066
**CVD**^**5**^	0.97 (0.80–1.17)	0.758
**Overweight**^**6**^	1.76 (1.51–2.05)	<0.001

(1) Total cholesterol > 5 mmol/L or low-density lipoprotein (LDL) > 3.0 mmol/L or high-density lipoprotein (HDL) < 1.2 mmol/L or Triglycerides > 1.7 mmol/L or Self-reported treatment.

(2) HIV negative—defined as negative self-report and negative on assay analysis.

(3) Age ≥60 years.

(4) Variable constructed incorporating measures of wealth, using methodology created for Demographic Health Surveys (DHS) divide in 5 quintiles–higher SES included 4^th^ and 5^th^ higher quintiles.

(5) Cardiovascular disease—self-report of Stroke/Myocardial Infarction/Angina or Angina by Rose criteria.

(6) Body mass index ≥ 25 kg/m^2^.

**Table 6:** Variables independently associated to dyslipidemia awareness^1^ in the HAALSI population.

Variable	Dyslipidemia	
	OR (95% CI)	*p*
**Gender (Female)**	1.55 (0.69–3.48)	0.286
**HIV negative**^**2**^	2.13 (0.63–7.21)	0.222
**Elderly**^**3**^	1.87 (0.84–4.15)	0.123
**Immigrant**	1.50 (0.68–3.32)	0.319
**SES**^**4**^ **(higher)**	1.76 (0.83–3.73)	0.137
**CVD**^**5**^	1.31 (0.53–3.23)	0.562
**Overweight**^**6**^	2.58 (1.19–5.58)	0.017

(1) Ever told by a clinical practitioner that you have high cholesterol.

(2) HIV negative—defined as negative self-report and negative on assay analysis.

(3) Age ≥60 years.

(4) Variable constructed incorporating measures of wealth, using methodology created for Demographic and Health Surveys (DHS) divided into 5 quintiles–higher SES included 4^th^ and 5^th^ quintiles.

(5) Cardiovascular disease—self-report of Stroke/Myocardial Infarction/Angina or Angina based on Rose criteria.

(6)Body mass index ≥ 25 kg/m^2^.

## Discussion

This analysis of the HAALSI cohort characterized a population which was middle-aged and older, overweight, and with a significant burden of dyslipidemia predominantly due to elevated triglycerides. Based upon the regression models, higher body weight was the only characteristic independently associated with of both dyslipidemia and dyslipidemia awareness. Awareness was also very rare in this population. HIV positivity and HIV treatment were not associated with higher rates of dyslipidemia. Specifically, 70.0% of HIV-positive subjects not on anti-retroviral therapy were dyslipidemic, while only 62.4% of those on therapy were (p = 0.01)–which was the opposite of the expected effect of anti-retroviral therapy. The absence of protease inhibitors in first line HIV treatment in Agincourt partially explains this[25]. Lipid lowering therapy was very low regardless of HIV treatment status so interaction with medical care also is not an explanation. A relatively small subset of the population was found to be at high or very high risk of future cardiovascular events according to the Framingham Cardiovascular Risk Score, and this sub-population had poor control of their lipids. Furthermore, only 4 of 1,214 of subjects with measured dyslipidemia in this baseline survey were receiving allopathic medications for control of cholesterol levels–indicating that there is an opportunity for a risk-based approach to statin therapy in this population.

Comparing the results of the HAALSI analysis to other studies from Sub-Saharan Africa there are differences in the underlying populations and lipid measurement which can explain some of the variation. Compared to the SANHANES survey, the HAALSI population had a similar burden of total cholesterol elevation, but had roughly half the prevalence of low HDL despite the population being older[6].Total cholesterol elevation in the HAALSI cohort (25.4%) was very similar to a 2007 survey (25.6%) from the same Agincourt region by Thorogood at al. which was a survey restricted to adults 35 years and older[8]. Manning et al (2016) identified a much higher prevalence rate of 69% for total cholesterol elevation (though a lower threshold of 4.5mmol/L) in Cape Town, but this was for obese subjects with ages 18 and older[9]. Finally, Maimela et al(2016) performed a survey of 20–59 year old subjects in the Dikgale area of Limpopo Province in Northeastern South Africa, which found a similar prevalence level for elevated total cholesterol of 25.6%[7]. Strikingly, the rates of different lipid abnormalities in the HAALSI cohort closely approximate the most recently published United States NHANES estimates (2012) with an overall dyslipidemia prevalence of 53%, elevated LDL of 27%, low HDL of 23%, and high triglycerides of 30%[26].

This analysis indicates two primary opportunities for improvement in cardiovascular disease prevention and lipid management. First, there is a defined and relatively small subset of the population at greater calculated cardiovascular risk who could definitively benefit from statin therapy–which is known to be both safe and cost-effective in low resource settings[27, 28]. Second, is a large awareness gap for dyslipidemia with profoundly low rates of not only pharmacotherapy but also diet and exercise therapy–indicating a need to improve screening and programming for first line non-pharmacologic therapy.

There are several limitations of this descriptive study. Primarily, the sub-district population from which the cohort was drawn has been part of a HDSS since 1992. This creates a specific situation of long-term population surveillance that might overrate the results of dyslipidemia awareness, as previously shown in a hypertension analysis from the same cohort[24]. However, the levels of dyslipidemia awareness were sufficiently low that this effect is unlikely to have produced any significant bias. Secondly, the blood samples were not collected from strictly fasting subjects, which is known to potentially inflate triglyceride levels inaccurately [29].This potential effect was measured using a quality analysis from this data set, which identified that 76.0% of all subjects were, by self-report, not fasting a minimum of 8 hours at the time of blood sample collection. the proportion of fasting samples was significantly higher among non-dyslipidemic subjects (28.3%) when compared to dyslipidemic (22.4%) subjects (p<0.001). Similarly, the proportion of subjects fasting was also significantly lower for subjects with hypertriglyceridemia (17.8%) than those without (26%) (p<0.001). This difference was not seen for those categorized based on LDL cutoffs. Additionally, triglycerides and LDL (calculated based on total cholesterol and triglyceride levels) not a determinant in cardiovascular risk stratification given the use of Framingham Risk calculation per local guidelines and therefore our analysis–further minimizing the likelihood of meaningful miscategorization. The use of the CardioChek PA device for lipid measurement likely underestimates cardiovascular risk in our population. Per recent published validity assessments, the CardioChek PA seems to underestimate LDL, HDL, and total cholesterol, while being inaccurate by unbiased in its triglyceride measurements[30].This contrasts with prior studies which validated the CardioChek PA against laboratory-based methods[31, 32]. Unfortunately, there is no venous draw validation for lipid values in the HAALSI data set.

There are multiple strengths to this study. The assessment of lipid awareness, treatment, and control is unique for studies performed in this region. Additionally, the formation of the HAALSI cohort will provide a suitable platform for the investigation of possible interventions for dyslipidemia and related cardiometabolic disorders and provide a greater understanding of the dynamic changes in behavior and population characteristics with respect to non-communicable diseases. The assessment of both HIV and migrant status as risk factors, allows for a more practical assessment of potential cardiometabolic disease risk factors which are characteristic of both the local region and sub-Saharan Africa in general. Finally, the assessment of all major lipoproteins is rare in large community studies and adds to the nuance of the information this paper provides on cardiovascular risk from lipid abnormalities.

## Conclusion

In conclusion, this baseline examination of the HAALSI cohort confirms the suspected notable contribution of dyslipidemia on overall cardiovascular risk in the Agincourt sub-district of rural South Africa, and demonstrates that there are multiple opportunities to allay this burden. Firstly, there is a subset of the population at high risk for cardiovascular events who could benefit from medical management with statin therapy, in the context of almost no current use. Secondly, our regression analysis and descriptive statistics demonstrates that overweight and obesity appear the primary modifiable risk factor to be targeted. Thirdly, the very low awareness of dyslipidemia in the population indicates a greater need for systematic screening and education within the population. It is hoped that ongoing HAALSI cohort investigations will further illuminate strategies and opportunities for dyslipidemia–and broader cardiometabolic disease—prevention and control in rural South and southern Africa.

## Supporting information

S1 Survey HAALSI survey original. (DOCX)

## References

[1] Mathers CD, Loncar D. Projections of global mortality and burden of disease from 2002 to 2030. PLoS medicine. 2006;3(11):e442 Epub 2006/11/30. doi: 10.1371/journal.pmed.0030442 ; PubMed Central PMCID: PMCPMC1664601. 17132052 10.1371/journal.pmed.0030442 PMC1664601

[2] GBD 2013 Mortality and Causes of Death Collaborators, Naghavi M, Wang H, Lozano R, Davis A, Liang X, et al Global, regional, and national age-sex specific all-cause and cause-specific mortality for 240 causes of death, 1990–2013: a systematic analysis for the Global Burden of Disease Study 2013. Lancet. 2015;385(9963):117–71. PubMed Central PMCID: PMCPMC4340604. doi: 10.1016/S0140-6736(14)61682-2 25530442 10.1016/S0140-6736(14)61682-2 PMC4340604

[3] Feigin VL, Roth GA, Naghavi M, Parmar P, Krishnamurthi R, Chugh S, et al Global burden of stroke and risk factors in 188 countries, during 1990–2013: a systematic analysis for the Global Burden of Disease Study 2013. The Lancet Neurology. 15(9):913–24. doi: 10.1016/S1474-4422(16)30073-4 27291521 10.1016/S1474-4422(16)30073-4

[4] Murray CJ, Barber RM, Foreman KJ, Abbasoglu Ozgoren A, Abd-Allah F, Abera SF, et al Global, regional, and national disability-adjusted life years (DALYs) for 306 diseases and injuries and healthy life expectancy (HALE) for 188 countries, 1990–2013: quantifying the epidemiological transition. Lancet (London, England). 2015;386(10009):2145–91. Epub 2015/09/01. doi: 10.1016/s0140-6736(15)61340-x ; PubMed Central PMCID: PMCPMC4673910. 26321261 10.1016/S0140-6736(15)61340-X PMC4673910

[5] Barquera S, Pedroza-Tobias A, Medina C, Hernandez-Barrera L, Bibbins-Domingo K, Lozano R, et al Global Overview of the Epidemiology of Atherosclerotic Cardiovascular Disease. Archives of medical research. 2015;46(5):328–38. Epub 2015/07/03. doi: 10.1016/j.arcmed.2015.06.006 . 26135634 10.1016/j.arcmed.2015.06.006

[6] Shisana O LD, Rehle T, Simbayi L, Zuma K, Dhansay A, Reddy P, et al South African National Health and Nutrition Examination Survey (SANHANES-1). Cape Town, South Africa: Human Sciences Research Council and MRC, 2013.

[7] Maimela E, Alberts M, Modjadji SE, Choma SS, Dikotope SA, Ntuli TS, et al The Prevalence and Determinants of Chronic Non-Communicable Disease Risk Factors amongst Adults in the Dikgale Health Demographic and Surveillance System (HDSS) Site, Limpopo Province of South Africa. PLoS One. 2016;11(2):e0147926 Epub 2016/02/18. doi: 10.1371/journal.pone.0147926 ; PubMed Central PMCID: PMCPMC4755539. 26882033 10.1371/journal.pone.0147926 PMC4755539

[8] Thorogood M, Connor M, Tollman S, Hundt GL, Fowkes G, Marsh J. A cross-sectional study of vascular risk factors in a rural South African population: data from the Southern African Stroke Prevention Initiative (SASPI). BMC public health. 2007;7(1):326. 17999764 10.1186/1471-2458-7-326 PMC2206028

[9] Manning K, Senekal M, Harbron J. Non-communicable disease risk factors and treatment preference of obese patients in Cape Town. African Journal of Primary Health Care & Family Medicine. 2016;8(1):913 doi: 10.4102/phcfm.v8i1.913 27380784 10.4102/phcfm.v8i1.913 PMC4926721

[10] Levitt NS, Steyn K, Dave J, Bradshaw D. Chronic noncommunicable diseases and HIV-AIDS on a collision course: relevance for health care delivery, particularly in low-resource settings—insights from South Africa. The American journal of clinical nutrition. 2011;94(6):1690s–6s. Epub 2011/11/18. doi: 10.3945/ajcn.111.019075 ; PubMed Central PMCID: PMCPMC3226022. 22089433 10.3945/ajcn.111.019075 PMC3226022

[11] Grover SA, Coupal L, Gilmore N, Mukherjee J. Impact of dyslipidemia associated with Highly Active Antiretroviral Therapy (HAART) on cardiovascular risk and life expectancy. The American journal of cardiology. 2005;95(5):586–91. Epub 2005/02/22. doi: 10.1016/j.amjcard.2004.11.004 . 15721096 10.1016/j.amjcard.2004.11.004

[12] Gaziano TA, Abrahams-Gessel S, Gomez-Olive FX, Wade A, Crowther NJ, Alam S, et al Cardiometabolic risk in a population of older adults with multiple co-morbidities in rural south africa: the HAALSI (Health and Aging in Africa: longitudinal studies of INDEPTH communities) study. BMC Public Health. 2017;17(1):206 doi: 10.1186/s12889-017-4117-y ; PubMed Central PMCID: PMC5314614. 28212629 10.1186/s12889-017-4117-y PMC5314614

[13] Kahn K, Collinson MA, Gómez-Olivé FX, Mokoena O, Twine R, Mee P, et al Profile: Agincourt Health and Socio-demographic Surveillance System. International Journal of Epidemiology. 2012;41(4):988–1001. doi: 10.1093/ije/dys115 22933647 10.1093/ije/dys115 PMC3429877

[14] Mancia G, Fagard R, Narkiewicz K, Redon J, Zanchetti A, Böhm M, et al 2013 ESH/ESC Guidelines for the management of arterial hypertension. European Heart Journal. 2013;34(28):2159 doi: 10.1093/eurheartj/eht151 23771844 10.1093/eurheartj/eht151

[15] Lloyd-Jones DM, Wilson PW, Larson MG, Beiser A, Leip EP, D'Agostino RB, et al Framingham risk score and prediction of lifetime risk for coronary heart disease. The American journal of cardiology. 2004;94(1):20–4. Epub 2004/06/29. doi: 10.1016/j.amjcard.2004.03.023 . 15219502 10.1016/j.amjcard.2004.03.023

[16] Klug EQ, Raal F, Marais A, Taskinen M, Dalby A, Schamroth C, et al South African Dyslipidaemia Guideline Consensus StatementA joint statement from the South African Heart Association (SA Heart) and the Lipid and Atherosclerosis Society of Southern Africa (LASSA): guidelines. South African Family Practice. 2015;57(2):22, 4–31.

[17] World Health Organization. Consolidated guidelines on HIV prevention, diagnosis, treatment and care for key populations: World Health Organization; 2014. 25996019

[18] World Health Organization (WHO). Consolidated guidelines on HIV testing services 2015. Geneva, Switzerland: World Health Organization, 2015.

[19] The uses of epidemiology in the study of the elderly. Report of a WHO Scientific Group on the Epidemiology of Aging. World Health Organization technical report series. 1984;706:1–84. Epub 1984/01/01. . 6437089

[20] Obesity: preventing and managing the global epidemic. Report of a WHO consultation. World Health Organization technical report series. 2000;894. 11234459

[21] Rutstein SO, Johnson K, MEASURE OM. The DHS wealth index: ORC Macro, MEASURE DHS; 2004.

[22] Rutstein SO. Steps to constructing the new DHS Wealth Index. Available from: http://dhsprogram.com/programming/wealth%20index/Steps_to_constructing_the_new_DHS_Wealth_Index.pdf [Date Accessed: March 18, 2016]: 2014.

[23] Achterberg S, Soedamah-Muthu SS, Cramer MJ, Kappelle LJ, van der Graaf Y, Algra A. Prognostic value of the Rose questionnaire: a validation with future coronary events in the SMART study. Eur J Prev Cardiol. 2012;19(1):5–14. Epub 2011/04/01. doi: 10.1177/1741826710391117 . 21450623 10.1177/1741826710391117

[24] Jardim TV, Reiger S, Abrahams-Gessel S, Gomez-Olive FX, Wagner RG, Wade A, et al Hypertension management in a population of older adults in rural South Africa. J Hypertens. 2017;35(6):1283–9. Epub 2017/04/26. doi: 10.1097/HJH.0000000000001312 . 28441697 10.1097/HJH.0000000000001312 PMC5505070

[25] Church K, Kiweewa F, Dasgupta A, Mwangome M, Mpandaguta E, Gomez-Olive FX, et al A comparative analysis of national HIV policies in six African countries with generalized epidemics. Bull World Health Organ. 2015;93(7):457–67. Epub 2015/07/15. doi: 10.2471/BLT.14.147215 ; PubMed Central PMCID: PMCPMC4490813. 26170503 10.2471/BLT.14.147215 PMC4490813

[26] Toth PP, Potter D, Ming EE. Prevalence of lipid abnormalities in the United States: the National Health and Nutrition Examination Survey 2003–2006. Journal of clinical lipidology. 2012;6(4):325–30. Epub 2012/07/28. doi: 10.1016/j.jacl.2012.05.002 . 22836069 10.1016/j.jacl.2012.05.002

[27] Collins R, Reith C, Emberson J, Armitage J, Baigent C, Blackwell L, et al Interpretation of the evidence for the efficacy and safety of statin therapy. The Lancet. 388(10059):2532–61. doi: 10.1016/s0140-6736(16)31357-5 10.1016/S0140-6736(16)31357-5 27616593

[28] Lim SS, Gaziano TA, Gakidou E, Reddy KS, Farzadfar F, Lozano R, et al Chronic Disease 4—Prevention of cardiovascular disease in high-risk individuals in low-income and middle-income countries: health effects and costs. The Lancet. 2007;370(9604):2054–62. 10.1016/S0140-6736(07)61699-7 18063025

[29] Sundvall J, Leiviska J, Laatikainen T, Peltonen M, Salomaa V, Vanhala M, et al The use of fasting vs. non-fasting triglyceride concentration for estimating the prevalence of high LDL-cholesterol and metabolic syndrome in population surveys. BMC Med Res Methodol. 2011;11:63 Epub 2011/05/17. doi: 10.1186/1471-2288-11-63 ; PubMed Central PMCID: PMCPMC3112195. 21569280 10.1186/1471-2288-11-63 PMC3112195

[30] Park PH, Chege P, Hagedorn IC, Kwena A, Bloomfield GS, Pastakia SD. Assessing the accuracy of a point-of-care analyzer for hyperlipidaemia in western Kenya. Tropical Medicine & International Health. 2016;21(3):437–44. doi: 10.1111/tmi.12653 26663749 10.1111/tmi.12653

[31] Ferreira CE, Franca CN, Correr CJ, Zucker ML, Andriolo A, Scartezini M. Clinical correlation between a point-of-care testing system and laboratory automation for lipid profile. Clin Chim Acta. 2015;446:263–6. Epub 2015/05/09. doi: 10.1016/j.cca.2015.04.036 . 25952166 10.1016/j.cca.2015.04.036

[32] Cohen M, Boyle E, Delaney C, Shaw J. A comparison of blood glucose meters in Australia. Diabetes Res Clin Pract. 2006;71(2):113–8. Epub 2005/07/14. doi: 10.1016/j.diabres.2005.05.013 . 16011856 10.1016/j.diabres.2005.05.013

