# Supplementary material for: Awareness, treatment, and control of dyslipidemia in rural South Africa: The HAALSI (Health and Aging in Africa: A Longitudinal Study of an INDEPTH Community in South Africa) study
Source: PLoS One. 2017 Oct 27;12(10):e0187347. doi: 10.1371/journal.pone.0187347 (PMC5659770; doi:10.1371/journal.pone.0187347)
Supplement: S1 Survey — (DOCX) [file pone.0187347.s001.docx]

**Section: Household Consumption**

| HC001 | We wish to know your permanent household members' food expenditure and consumption for the last 30 days. Are you the primary person who purchases food for the household? [IWER: IF RESPONDENT IS NOT A MAIN FOOD PURCHASER, SAY: PLEASE FEEL FREE TO GET SOME HELP IN ANSWERING THE FOLLOWING FOOD-RELATED QUESTIONS.] | YES................................................1  NO ................................................2 |
| --- | --- | --- |
|  |  |  |
| HC002 | In the last 30 days, did your permanent household purchase any of the following? | [Staple foods (mielies, pasta, potatoes, bread, crackers, rice, wheat, sorghum, potatoes, cassava roots)]..............................1  [Meat (Beef, pork, mutton, chicken, turkey, guineafowl, game, wild birds, fish, sausage, canned meat, polony)]..............................2  [Fruits and vegetables (Apple, banana, guava, avocado, orange, naartjie, lemon, grape, raisin, melon, grapefruit, pineapple, mulberry, wild fruit), (butternut, carrot, spinach, muroho, cabbage, broccoli etc)]..............................................3  [Milk and eggs].............................4  [Spices and oils (salt, sugar, curry, garlic, ginger, pepper, chilli, or other - butter, lard, cooking oils (coconut oil, palm oil, vegetable oil, corn oil)].................................5  [Beverages and other non-alcoholic drinks such as coffee, tea, juice, water and soft drinks.]…………………………….……...6  [Tobacco and alcoholic beverages (including, beer, wine, spirits)]...7  [Food eaten outside the dwelling (for example, at vendors, kiosks or restaurants)].................................8  [Other food items]……..................9  [Edible insects]...........................10 |
|  |  |  |
| HC003 | In the last 30 days, how much (value in Rand) did your permanent household spend and consume on: | [Staple foods (mielies, pasta, potatoes, bread, crackers, rice, wheat, sorghum, potatoes, cassava roots)] ___________Rand  [Meat (Beef, pork, mutton, chicken, turkey, guineafowl, game, wild birds, fish, sausage, canned meat, polony)]____________Rand  [Fruits and vegetables (Apple, banana, guava, avocado, orange, naartjie, lemon, grape, raisin, melon, grapefruit, pineapple, mulberry, wild fruit), (butternut, carrot, spinach, muroho, cabbage, broccoli etc)]_____________Rand  [Milk and eggs]___________Rand  [Spices and oils (salt, sugar, curry, garlic, ginger, pepper, chilli, or other - butter, lard, cooking oils (coconut oil, palm oil, vegetable oil, corn oil))]_____________Rand  [Beverages and other non-alcoholic drinks such as coffee, tea, juice, water and soft drinks.]__________________Rand  [Tobacco and alcoholic beverages (including, beer, wine, spirits)]__________________Rand  [Food eaten outside the dwelling (for example, at vendors, kiosks or restaurants)]_____________Rand  [Other food items]_________Rand  [Edible insects]___________Rand |
|  |  |  |
| HC004 | Did your permanent household produce any:  Staple foods (mielies, pasta, potatoes, bread, crackers, rice, wheat, sorghum, potatoes, cassava roots)  Meat (Beef, pork, mutton, chicken, turkey, guineafowl, game, wild birds, fish, sausage, canned meat, polony)  Fruits and vegetables (Apple, banana, guava, avocado, orange, naartjie, lemon, grape, raisin, melon, grapefruit, pineapple, mulberry, wild fruit), (butternut, carrot, spinach, muroho, cabbage, broccoli etc)  Milk and eggs  Spices and oils (salt, sugar, curry, garlic, ginger, pepper, chilli, or other - butter, lard, cooking oils (coconut oil, palm oil, vegetable oil, corn oil))  Beverages and other non-alcoholic drinks such as coffee, tea, juice, water and soft drinks.  Tobacco and alcoholic beverages (including, beer, wine, spirits)  Food eaten outside the dwelling (for example, at vendors, kiosks or restaurants)  Other food items  Edible insects | YES................................................1  NO................................................2 |
|  |  |  |
| HC005 | What is the value of home produced…  Staple foods (mielies, pasta, potatoes, bread, crackers, rice, wheat, sorghum, potatoes, cassava roots)  Meat (Beef, pork, mutton, chicken, turkey, guineafowl, game, wild birds, fish, sausage, canned meat, polony)  Fruits and vegetables (Apple, banana, guava, avocado, orange, naartjie, lemon, grape, raisin, melon, grapefruit, pineapple, mulberry, wild fruit), (butternut, carrot, spinach, muroho, cabbage, broccoli etc)  Milk and eggs  Spices and oils (salt, sugar, curry, garlic, ginger, pepper, chilli, or other - butter, lard, cooking oils (coconut oil, palm oil, vegetable oil, corn oil))  Beverages and other non-alcoholic drinks such as coffee, tea, juice, water and soft drinks.  Tobacco and alcoholic beverages (including, beer, wine, spirits)  Food eaten outside the dwelling (for example, at vendors, kiosks or restaurants)  Other food items  Edible insects  …that you consumed? | ________________________Rand |
|  |  |  |
| HC006 | Did you eat any food in the last 30 days that the permanent household grew or collected itself (crops animals insects) that you did not pay for? | YES................................................1  NO ................................................2 |

**Section: Household Expenditure**

| HE001 | Now I'll ask you about expenditures in the last month for your permanent household for the following items. Right now, I am only asking about expenditures during the last 30 days.  In the past 30 days, approximately how much (in Rand) did your household spend on communication fees, including postage, Internet, telephone, mobile phone, and others? | ________________________Rand |
| --- | --- | --- |
|  |  |  |
| HE002 | In the past 30 days, approximately how much (in Rand) did your permanent household spend on utilities, such as water, electricity, waste disposal, etc.? | ________________________Rand |
|  |  |  |
| HE003 | In the past 30 days, approximately how much (in Rand) did your permanent household spend on fuels (gas, coal, kerosene, firewood, petrol, diesel, etc.)? | ________________________Rand |
|  |  |  |
| HE004 | In the past 30 days, approximately how much (in Rand) did your permanent household spend on payments for household servants, including cook, maid, driver, security, gardener, etc.? | ________________________Rand |
|  |  |  |
| HE005 | In the past 30 days, approximately how much (in Rand) did your permanent household spend on local transportation (to work place, market etc)? | ________________________Rand |
|  |  |  |
| HE006 | In the past 30 days, approximately how much (in Rand) did your permanent household spend on household items. such as spoons, pots, etc. (purchase of new items)? | ________________________Rand |
|  |  |  |
| HE007 | In the past 30 days, approximately how much (in Rand) did your permanent household spend on personal toiletries and personal care (e.g., soap, toothpaste, toothbrush, cosmetics, beauty salon, etc.)? | ________________________Rand |
|  |  |  |
| HE008 | In the past 30 days, approximately how much (in Rand) did your permanent household spend on entertainment, including games, table pool, stadium soccer matches, or attending bashes? | ________________________Rand |
|  |  |  |
| HE009 | In the past 30 days, approximately how much (in Rand) did your permanent household spend on clothing and bedding? | ________________________Rand |
|  |  |  |
| HE010 | In the past 30 days, approximately how much (in Rand) did your permanent household spend on monthly rent? | ________________________Rand |
|  |  |  |
| HE011 | In the past 30 days, approximately how much (in Rand) did your permanent household spend on mortgage or loan repayment on your house? | ________________________Rand |
|  |  |  |
| HE012 | Now I will ask you about your permanent household's expenditures in the last 12 months. Please tell me the expenditures in the last 12 months for your permanent household for the following items. Right now, I am only asking about expenditures during the last 12 months.  In the last 12 months, how much (in Rand) did your permanent household spend on long distance travel expenses, including travel by train, car, bus, and plane? | ________________________Rand |
|  |  |  |
| HE013 | In the last 12 months, how much (in Rand) did your permanent household spend on wedding expenses including gifts and lobola? | ________________________Rand |
|  |  |  |
| HE014 | In the last 12 months, how much (in Rand) did your permanent household spend on birthdays? | ________________________Rand |
|  |  |  |
| HE015 | In the last 12 months, how much (in Rand) did your permanent household spend on funerals? | ________________________Rand |
|  |  |  |
| HE016 | In the last 12 months, how much (in Rand) did your permanent household spend on festivals and religious ceremonies, including festival clothes, gifts, foods, etc.? | ________________________Rand |
|  |  |  |
| HE017 | In the last 12 months, how much (in Rand) did your permanent household spend on education and training, including tuition, school/training fees, books, uniforms and other related expenses (including hostel/ meals charges related to education)? | ________________________Rand |
|  |  |  |
| HE018 | In the last 12 months, how much (in Rand) did your permanent household spend on insurance premiums for furniture, electronics, life, health, hospital, funeral, car, legal insurance, etc? | ________________________Rand |
|  |  |  |
| HE019 | In the last 12 months, how much (in Rand) did your permanent household spend on home maintenance and repair expenses (not including additions to your house)? | ________________________Rand |
|  |  |  |
| HE020 | In the last 12 months, how much (in Rand) did your permanent household spend on vehicle service charges, including car, truck, bicycles, and other vehicles? | ________________________Rand |
|  |  |  |
| HE021 | In the last 12 months, how much (in Rand) did your permanent household spend on taxes, fees, registration, and service charges paid to the government, including income tax and annual taxes on land granted by the Traditional Authority? | ________________________Rand |
|  |  |  |
| HE022 | In the last 12 months, how much (in Rand) did your permanent household spend on doctor, nurse and dentists fees? | ________________________Rand |
|  |  |  |
| HE023 | In the last 12 months, how much (in Rand) did your permanent household spend on other hospital and clinic fees? | ________________________Rand |
|  |  |  |
| HE024 | In the last 12 months, how much (in Rand) did your permanent household spend on medicines, bandages, medical supplies, chemist and pharmacy purchases? | ________________________Rand |
|  |  |  |
| HE025 | In the last 12 months, how much (in Rand) did your permanent household spend on traditional healer’s fees? | ________________________Rand |
|  |  |  |
| HE026 | In the last 12 months, how much (in Rand) did your permanent household spend on any other health care costs? | ________________________Rand |
|  |  |  |
| HE027 | In the last 12 months, how much (in Rand) did your household spend on other loan re-payments, including interest? | ________________________Rand |
|  |  |  |
| HE028 | In the last 12 months, how much (in Rand) did your permanent household spend on donation to charities, churches, organization, etc.? | ________________________Rand |
|  |  |  |
| HE029 | Please tell me all other expenses that occurred during the past year that we have not already talked about (not including food and drink, which will be covered separately). How much did you spend on these other expenses? | ________________________Rand |
|  |  |  |
| HE030 | Could you please specify what this additional expenditure was for? |  |

**Section: Household Labor Income**

| HL001 | Now I will ask you about the members of your permanent household who worked in the last month.  In the last month, which of the following members of your permanent household worked for a wage, salary, commission or any payment in kind; including doing paid domestic work? | 1 ^HHMemberNameOver12[1] 2 ^HHMemberNameOver12[2] 3 ^HHMemberNameOver12[3] 4 ^HHMemberNameOver12[4] 5 ^HHMemberNameOver12[5] 6 ^HHMemberNameOver12[6] 7 ^HHMemberNameOver12[7] 8 ^HHMemberNameOver12[8] 9 ^HHMemberNameOver12[9] 10 ^HHMemberNameOver12[10] 11 ^HHMemberNameOver12[11] 12 ^HHMemberNameOver12[12] 13 ^HHMemberNameOver12[13] 14 ^HHMemberNameOver12[14] 15 ^HHMemberNameOver12[15] 16 ^HHMemberNameOver12[16] 17 ^HHMemberNameOver12[17] 18 ^HHMemberNameOver12[18] 19 ^HHMemberNameOver12[19] 20 ^HHMemberNameOver12[20] 99 None |
| --- | --- | --- |
|  |  |  |
| HL002 | How much was (HHMemberName)'s last monthly payment before taxes and deductions? (Rand) | ________________________Rand |
|  |  |  |

**Section: Business Income**

| BI001 | Over the past 12 months, has anyone in your permanent household operated any business which produces goods or services (for example, artisan, metalworking, tailoring, repair work; also include processing and selling your own crops or animals) or has anyone in your household owned a shop or operated a trading business or profession? | \| YES.................................................1  NO.................................................2 \| \| --- \| |
| --- | --- | --- | --- |
|  |  |  |
| BI002 | Please describe the business. What are the main goods/services produced at this business or what is the business’s main function? [IWER: DESCRIBE THE INDUSTRY E.G. FARM, RETAURANT, GROCERY STORES, MARKET STALL ETC] | Bottle store...................................1  Supermarket.................................2  Pharmacy......................................3  Surgery…………………………………….4  Private/preschool.........................5  Driving school...............................6  Shop (any type)............................7  Car towing…….....................................8  Funeral parlour.............................9  Large equipment hire................10  Money lender.............................11  Hawkers......................................12  Tailors……….................................13  Hair dresser……..................................14  Mechanic....................................15  Artisan........................................16  Beer brewing.......................................17  Other………………..……………………18 |
|  |  |  |
| BI003_a | What were the total sales from this business over the past year? | ________________________Rand |
|  |  |  |
| BI003_b | What were the total expenses from this business over the last year? | ________________________Rand |
|  |  |  |
| BI003_c | Is this the total profit that was available to you last year? | ________________________Rand |
|  |  |  |
| BI004 | Which members of the permanent HH were involved in this business in the last year? [IWER: RECORD MEMBERS FOR ALL HOUSEHOLD MEMBERS THAT ARE MENTIONED] | 1 ^HHMemberNamePermanent[1] 2 ^HHMemberNamePermanent[2] 3 ^HHMemberNamePermanent[3] 4 ^HHMemberNamePermanent[4] 5 ^HHMemberNamePermanent[5] 6 ^HHMemberNamePermanent[6] 7 ^HHMemberNamePermanent[7] 8 ^HHMemberNamePermanent[8] 9 ^HHMemberNamePermanent[9] 10 ^HHMemberNamePermanent[10] 11 ^HHMemberNamePermanent[11] 12 ^HHMemberNamePermanent[12] 13 ^HHMemberNamePermanent[13] 14 ^HHMemberNamePermanent[14] 15 ^HHMemberNamePermanent[15] 16 ^HHMemberNamePermanent[16] 17 ^HHMemberNamePermanent[17] 18 ^HHMemberNamePermanent[18] 19 ^HHMemberNamePermanent[19] 20 ^HHMemberNamePermanent[20] |
|  |  |  |
| BI005 | Over the past 12 months, has anyone in your household operated any **other** business which produces goods or services or has anyone else in your household owned a shop or operated a trading business or profession? | YES................................................1  NO ................................................2 |

**Section: Government Transfers**

| GT001 | Now I will ask about any government subsidies or transfers your permanent household members received in the last month. Did your household receive any of the following government subsidies or transfers in the last month? | \| Care dependency……....................1  Disability……..................................2  Child support.................................3  Foster care..…………………….……….4  Additional Aid grant......................5  Old age………..................................6  Social relief of distress..................7  War veterans.................................8  Unemployment insurance fund..9  Other (specify)….........................10  None……………..............................11 \| \| --- \| |
| --- | --- | --- | --- |
|  |  |  |
| GT002 | How many people in the household received this (subsidy or transfer)? | Number:_____________________ |
|  |  |  |
| GT003 | What is the total amount you received from (this subsidy or transfer) in the past month? (Rand) | ________________________Rand |

**Section: Remittances**

| RS001 | Now I will ask about any remittances your permanent household may have received. Did your permanent household receive any remittances from anybody living elsewhere? | YES................................................1  NO ................................................2 |
| --- | --- | --- |
|  |  |  |
| RS002 | Did any remitter (outside your permanent household) provide school fees, money, food, clothing, and/or something else, in the last 12 months? | YES................................................1  NO................................................2 |
|  |  |  |
| RS003 | Please tell me who gave the largest remittances your permanent household in the past 12 months? Please select the person from the list or enter the relationship of the remitter to the household head. | ^HHMemberNameNonPermanent[1] ^HHMemberNameNonPermanent[2] ^HHMemberNameNonPermanent[3] ^HHMemberNameNonPermanent[4] ^HHMemberNameNonPermanent[5] ^HHMemberNameNonPermanent[6] ^HHMemberNameNonPermanent[7] ^HHMemberNameNonPermanent[8] ^HHMemberNameNonPermanent[9] ^HHMemberNameNonPermanent[10] ^HHMemberNameNonPermanent[11] ^HHMemberNameNonPermanent[12] ^HHMemberNameNonPermanent[13] ^HHMemberNameNonPermanent[14] ^HHMemberNameNonPermanent[15] ^HHMemberNameNonPermanent[16] ^HHMemberNameNonPermanent[17] ^HHMemberNameNonPermanent[18] ^HHMemberNameNonPermanent[19] ^HHMemberNameNonPermanent[20] 99 Other, please specify |
|  |  |  |
| RS004 | Did your permanent household receive any part of this remittance as cash? | YES................................................1  NO ................................................2 |
|  |  |  |
| RS005 | In the last 12 months how much did the donor send? (Rand) | ________________________Rand |
|  |  |  |
| RS006 | Did your permanent household receive any part of this remittance as non-cash? | YES................................................1  NO ................................................2 |
|  |  |  |
| RS007 | For the last 12 months, what would be your best estimate for the total money value of these non-cash remittances that your permanent household received? (Rand) | ________________________Rand |
|  |  |  |
| RS008 | Did anyone else send remittances to your permanent household in the past 12 months? | YES................................................1  NO ................................................2 |
|  |  |  |
| RS009 | In the last 12 months did your household receive any gifts, donations, inheritance, transfers of cash, or items such as food from charities, or religious or other groups? Please do not include any remittances you might have already mentioned in the previous question. | YES................................................1  NO ................................................2 |
|  |  |  |
| RS010 | For the last 12 months, what would be your best estimate for the total money value of these gifts that your household received? | ________________________Rand |
|  |  |  |
| RS011 | In the last 12 months, did your permanent household have any other income that we have not already discussed? | YES................................................1  NO ................................................2 |
|  |  |  |
| RS012 | What is this other household income that we have not already discussed? | ____________________________ |
|  |  |  |
| RS013 | What was the total amount of this other income in the last 12 months? | ________________________Rand |

**Section: Durables**

| DS001 | Now I will ask about the permanent household's ownership of durable goods. Please tell me about any functional vehicles your permanent household owns. Please feel free to ask someone else for help.  Does your household own any of the following? | Cars…………………………….................1  Trucks…..…….................................2  Any other automobiles, including 3 wheelers (e.g tractors)..............3  Bicycles……..………………………..…….4  Motorcycles………….......................5  Scooters.…….................................6  Carts/sleds………...........................7 |
| --- | --- | --- |
|  |  |  |
| DS002 | How many (of the above vehicles) does the permanent household own? | Specify______________________ |
|  |  |  |
| DS003 | Did you purchase any of these (vehicles) in past 12 months? | YES................................................1  NO ................................................2 |
|  |  |  |
| DS004 | How much did it cost? (Rand) [IWER: IF MORE THAN ONE, ENTER COST OF LAST ONE PURCHASED] | ________________________Rand |
|  |  |  |
| DS005 | Did your permanent household receive any income from renting/leasing these (vehicles) in the past 12 months? | YES................................................1  NO ................................................2 |
|  |  |  |
| DS006 | What was the amount of rental income your permanent household received in the past 12 months from these (vehicles)? (Rand) | ________________________Rand |
|  |  |  |
| DS007 | Does your permanent household own any of the following? | Refrigerator……………………………….1 Washing machine…………….………..2 Sewing machine………………….……..3 Tube TV……………………………………..4 Flat screen TV……………….……………5 Video or Dvd Player…………………...6 Satellite Dish…………………….………..7 Radio (stand-alone)………….………..8 Computer/laptop………..……………..9 Stereo system…………………….……10 Camera, video camera……….…….11 Air conditioner………….……………..12 Ordinary cell phone…….……………13 Smart phone………………….………..14 Cement mixer or grinder…….……15 Clock or watch………………………...16 Pressure cooker……….………………17 Sofa………….………………………………18 Bed………………………………………….19 Cot…………………………………………..20 Table………………………………………..21 Electric fan (ceiling fan and/or stand fan)………..……………………….22 Stove………………….…………………….23 Solar Energy………………….………….24 Power Generator………….………….25 Alternative power source…….…..26 Telephone (fixed line)……….……..27 Microwave………….……………………28 Internet by Computer………….…..29 Internet by mobile phone….…….30 Toilet Facilities……………….………..31 Tractor…………………………………….32 Plough………………….………………….33 |
|  |  |  |
| DS008 | How many (of the above products) does your household own? | Specify_____________________ |
|  |  |  |
| DS009 | Did your household purchase any of these (products) in the past 12 months? | YES................................................1  NO................................................2 |
|  |  |  |
| DS010 | How much did it cost? | ________________________Rand |

**Section: Housing**

| HG001 | The following questions pertain to your permanent household's dwelling structure. (Regardless of whether you own it or not, the dwelling structure where your permanent household lives most often is referred to as your dwelling structure.) Please feel free to get some help in answering the following questions.  Is your dwelling structure owned by a permanent household member(s)? | YES................................................1  NO ................................................2 |
| --- | --- | --- |
|  |  |  |
| HG002 | Are you renting/leasing your current residence? | YES................................................1  NO................................................2We don’t own it, but we are living there for free.………………………….3 |
|  |  |  |
| HG003 | How did your permanent household member acquire your current dwelling structure? Have you built it, bought it, inherited it, received from family or friends, received from the government, or by other means? | Built it……….………….……………………1 Bought it…………….……….…………….2 Inherited…………….…………………….3 Received from family or friends…………………………….………..4 Received from the government...5 Other…………….………………………….6 |
|  |  |  |
| HG004 | Excluding the dwelling structure in which your permanent household lives, do you or any other members of your permanent household own any other residential properties? | YES................................................1  NO ................................................2 |
|  |  |  |
| HG005 | How many other dwelling structures do you or members of your permanent household currently own? | ________________________Units |
|  |  |  |
| HG006 | Do you or your household rent/lease out any of those housing units or any part of the house you currently live in? | YES................................................1  NO ................................................2 |
|  |  |  |
| HG007 | What was the total rental income your permanent household received during the last twelve months? | ________________________Rand |
|  |  |  |
| HG008 | If you were to build your house from scratch today, how much would it cost? | ________________________Rand |

**Section: Land Ownership**

| LO001 | Now I will ask you some questions about land ownership.  Does your permanent household have land (a stand or agricultural area) allocated from the traditional authority? | YES................................................1  NO ................................................2 |
| --- | --- | --- |
|  |  |  |
| LO002 | How much land was allocated to your household from the traditional authority? [IWER: ENTER DIMENSIONS IN METERS] | Amount_____________________ |
|  |  |  |
| LO003 | Do you or other members of your household own any land (not including land you rent/lease from others)? | YES................................................1  NO ................................................2 |
|  |  |  |
| LO004 | If you were to sell all of your land today, what would be the total value that you will receive (before taxes and deductions)? | ________________________Rand |
|  |  |  |
| LO004_brackets | Within which interval would the estimate of your total land value lie? | None.……….………..………………………1 R 1 – 50000.……….…………..………….2 R 50001 – 250000……….……………..3 R 250001 – 1500000……….….……..4  More than R 1500001………….…….5 |
|  |  |  |
| LO005 | Did you rent/lease out any of your land in the past 12 months? | YES................................................1  NO ................................................2 |
|  |  |  |
|  |  |  |
| LO006_monthly | Average monthly income: | ________________________Rand |
|  |  |  |
| LO006_number | Number of months earned: | ______________________Months |
|  |  |  |
| LO006_total | Total rental income: | __________Rand/past 12 months |
|  |  |  |
| LO006_brackets | OR, Within which interval does the earned rental income from land in the past twelve months lie? | None.…………………………………………1 R 1 – 6000...……………………………….2 R 6001 – 18000….……………….……..3 R 18001 – 72000…….……………..…..4  More than R 72000….…………….….5 |
|  |  |  |
| LO008_acres | How much land did you rent /lease in the past twelve months? [IWER: PLEASE ROUND ANSWER TO 1 DECIMAL PLACE] | _______________________Acres |
|  |  |  |
| LO009_brackets | OR, Within which interval does the paid rental income from land in the past twelve months lie? | None.…………………………………………1 R 1 – 6000...……………………………….2 R 6001 – 18000….……………….……..3 R 18001 – 72000…….……………..…..4  More than R 72000….…………….….5 |
|  |  |  |
| LO009_intro | How much land rent did you pay in the past 12 months? |  |
|  |  |  |
| LO009_monthly | Average monthly rent: | ________________________Rand |
|  |  |  |
| LO009_number | Number of months paid: | ______________________Months |
|  |  |  |
| LO009_total | Total rental paid: | __________Rand/past 12 months |

**Section: Livestock**

| LK001 | Now I will ask about livestock ownership.  Do you/your permanent household own any livestock? | YES...............................................1  NO................................................2 |
| --- | --- | --- |
|  |  |  |
| LK002 | Overall, what is the value of all the livestock you own if you were to sell them today? | ________________________Rand |
|  |  |  |
| LK002_brackets | Within which interval does the total value of all livestock you own if you were to sell them today lie? | None.…………………………………………1 R 1 – 6000...……….….………………….2 R 6001 – 18000….……………….……..3 R 18001 – 72000…….………….……..4  More than R 72000….………….…….5 |
|  |  |  |
| LK003 | Does your household currently own any of the following? | Cows………………….………………………1 Goats………………….….……………..…..2 Horses………………..……………….…....3 Donkeys/Mules.………………………..4 Chickens…………….………………………5 Turkeys……………………………………..6 Other poultry.………….………………..7  Pigs……………….…………………………..8 Other…………….…………………………..9 |
|  |  |  |
| LK004 | How many (of the above animal) do you currently own? | ________________________Units |

**Section: Financial Assets**

| FA001 | Now I will ask you some questions about the financial assets of the permanent household members.  What is your best estimate of the total value of financial assets owned by the permanent household? Financial assets include cash money, bank accounts, deposits, shares, bonds and stocks. | ________________________Rand |
| --- | --- | --- |
|  |  |  |
| FA001_brackets | Within which interval does the total value of financial assets owned by the household lie? | None.…………………………………………1 R 1 – 50000.……….…..………………….2 R 50001 – 250000……………….……..3 R 250001 – 1500000…………………..4  More than R 1500001………….…….5 |
|  |  |  |
| FA002 | Did you or any of your household members receive any interest or dividends from your financial assets during the past year? | YES................................................1  NO ................................................2 |
|  |  |  |
| FA003 | What was the total returns on these financial investments you and your household received during the past year (before tax)? | ________________________Rand |
|  |  |  |
| FA003_brackets | Within which interval does the total return on these financial investments you and your household received during the past year lie? | None.……….……..…………………………1 R 1 – 6000...……….……..……………….2 R 6001 – 18000….…………….………..3 R 18001 – 72000…….……….….……..4  More than R 72000….….…………….5 |
|  |  |  |
| FA004 | Do you or members of your household possess any of the following financial assets? | Current accounts………….……………1 Savings accounts, postal accounts……………………………………2 Stocks or mutual funds……….……..3 Bonds………………..……………………...4 Outstanding balances in revolving funds, informal savings groups, etc……………………………………………..5 Stockvel……………….…………………….6 Other……………….………………………..7 |
|  |  |  |
| FA005 | What is the approximate current asset value of (the above asset)? | ________________________Rand |
|  |  |  |
| FA006 | Now I will ask about any outstanding loans.  Do you OR any of your permanent household members have any outstanding loans from banks and other institutions, such as shop credit, hire purchase, microfinance, NGOs, government, credit union or cooperatives, employer, local money lenders, family or friends? | YES................................................1  NO ................................................2 |
|  |  |  |
| FA007 | Whom did you or your household members borrow from? Please identify all institutions or individuals you or your household members borrowed money from form the list below. | Banks………….……………………………. 1 Cooperatives or other non-profit organizations………….………………… 2 Employer………………….………………. 3 Local money lenders……….……….. 4 Family members or friends…….….5 Local shops……….……………………… 6 Hire Purchase…………….…………….. 7 Other…………………….…………………. 8 |
|  |  |  |
|  |  |  |
| FA009 | How much do you or your household members currently owe these (institutions or individuals)? | ________________________Rand |
|  |  |  |
| FA010 | What is the average monthly installment that you have to pay? | __________________Rand/month |
|  |  |  |
| FA011 | What is the total value of all outstanding (unpaid) loans that you and your household have- excluding business loans? | ________________________Rand |
|  |  |  |
| FA011_brackets | Within which interval does the total value of all outstanding loans you and your household have lie? | None.…………………………………………1 R 1 – 50000.……….…..………………….2 R 50001 – 250000……………….……..3 R 250001 – 1500000…………………..4  More than R 1500001………….…….5 |
|  |  |  |
| FA012 | Are any of these loans for your primary dwelling? | YES................................................1  NO ................................................2 |
|  |  |  |
| FA013 | How much do you currently owe for home loans for your primary dwelling? | ________________________Rand |
|  |  |  |
| FA013_brackets | Within which interval does the total value of how much you currently owe for home loans for your primary dwelling lie? | None.…………………………………………1 R 1 – 50000.……….…..………………….2 R 50001 – 250000……………….……..3 R 250001 – 1500000…………………..4  More than R 1500001………….…….5 |
|  |  |  |
| FA014 | Are any of these loans (that you talked about) for your business? | YES................................................1  NO ................................................2 |
|  |  |  |
| FA015 | How much do you currently owe for business loans? | ________________________Rand |
|  |  |  |
| FA015_brackets | Within which interval does the total value of business loans owed lie? | None.…………………………………………1 R 1 – 50000.……….…..………………….2 R 50001 – 250000……………….……..3 R 250001 – 1500000…………………..4  More than R 1500001………….…….5 |
|  |  |  |
| FA016 | Please tell me about the largest asset/stockvel/informal group that your permanent HH member(s) participate in.  Do you get the money at the end? | YES...............................................1  NO................................................2No (other) stockvels……..…………3 |
|  |  |  |
| FA017 | How much have you put in? | ________________________Rand |
|  |  |  |
| FA018 | Is it with other participant members? | YES................................................1  NO ................................................2 |
|  |  |  |
| FA019 | What is its total balance today? | ________________________Rand |
|  |  |  |
| FA020 | How many participants? | __________________Participants |
|  |  |  |
| FA021 | For how many months have you participated? | ______________________Months |
|  |  |  |
| FA022 | How much to you put in each month? | ________________________Rand |
|  |  |  |
| FA023 | Have you already received your lumpsum? | YES................................................1  NO ................................................2 |

**Section: Visit Check**

| VC001_form1 | [IWER: HOUSEHOLD INFORMANT CONSENT (FORM 1).Make sure that:  it has been SIGNED and DATED by the field worker, it has been SIGNED and DATED by the respondent, a copy has been LEFT with the respondent, you KEPT a copy for our records] |  |
| --- | --- | --- |
|  |  |  |
| VC002_form2 | [IWER: INDIVIDUAL INFORMANT CONSENT (FORM 2). Make sure that: it has been SIGNED and DATED by the field worker, it has been SIGNED and DATED by the respondent, a copy has been LEFT with the respondent, you KEPT a copy for our records] |  |
|  |  |  |
| VC003_form3 | [IWER: INDIVIDUAL INFORMANT CONSENT FOR SAGE/HIV/NCD PARTICIPANTS (FORM 3). Make sure that: it has been SIGNED and DATED by the field worker, it has been SIGNED and DATED by the respondent, a copy has been LEFT with the respondent, you KEPT a copy for our records] |  |
|  |  |  |
| VC006 | We would now like to take your picture for our records. We will use the picture to confirm your identity when we follow up with you later in the study |  |
|  |  |  |
| VC006_form6 | [IWER: PROXY INFORMANT CONSENT (FORM 6). Make sure that: it has been SIGNED and DATED by the field worker, it has been SIGNED and DATED by the respondent, a copy has been LEFT with the respondent, you KEPT a copy for our records] |  |
|  |  |  |
| VC007 | Answer options | All consents upfront…………………..1 Consent at start, biomarker module and end…………………………2 |
|  |  |  |

**Section: Background**

| BD001 | Now I have some general questions about your background.  What is your date of birth? In which year and month were you born? | Month:______________________  Year:________________________ |
| --- | --- | --- |
|  |  |  |
| BD002 | How old are you (in completed years)? | ________________________Years |
|  |  |  |
| BD003 | What is your current marital status? | Never married..............................1  Currently married or living with partner……………............................2  Separated/Deserted.....................3  Divorced.......................................4  Widowed………………..…………..…….5 |
|  |  |  |
| BD004 | What is your spouse/partner’s name? [IWER: FOR MULTIPLE PARTNERS, REFER TO MAIN SPOUSE.] | Name_______________________ |
|  |  |  |
| BD005 | Does your spouse or partner live in this household? | YES................................................1  NO................................................2 |
|  |  |  |
| BD006 | [IWER: PLEASE IDENTIFY THE SPOUSE FROM THE HOUSEHOLD ROSTER] |  |
|  |  |  |
| BD007 | What is the primary reason that your spouse does not live in this household? [IWER: PROBE: IS IT BECAUSE OF HIS/HER JOB, SCHOOLING, HEALTH OR SOME OTHER REASON?] | Due to job……...............................1  Due to schooling..........................2  Due to health reasons..................3  Due to family reasons (taking care of old parents, etc.)......................4  Other…….………………………..…..…….5 |
|  |  |  |
| BD008 | How old is your spouse (completed years)? | ________________________Years |
|  |  |  |
| BD009 | Can your spouse read and write? | Able to read only..........................1  Able to write only…......................2  Able to both read and write.........3  Cannot read or write.………….…….4 |
|  |  |  |
| BD010 | Has your spouse ever attended school? | YES................................................1  NO ................................................2 |
|  |  |  |
| BD011 | How many years of schooling has your spouse had? | ________________________Years |
|  |  |  |
| BD012 | What is the highest level of school that your spouse has completed? | (N) None…....………………………………1 (R) Preschool……….………..…………..2 (A) Sub-A/Gr 1….………………………..3 (B) Sub-B/Gr 2.…………………………..4 (1) Std 1/Gr 3..……………………………5 (2) Std 2/Gr 4……………………………..6 (3) Std 3/Gr 5……………………………..7  (4) Std 4/Gr 6……………………………..8 (5) Std 5/Gr 7……………………………..9 (6) Std 6/Gr 8……………………………10 (7) Std 7/Gr 9……………..…………….11 (8) Std 8/Gr 9….………………………..12 (9) Std 9/Gr 11……….…………………13 (0) Stad 10/Gr 12/Matric…..……..14 (P) Partial Tertiary……………………15 (T) Completed Tertiary……..….....16 (A1) ABET 1..…….………………………17 (A2) ABET 2………………………………18 (A3) ABET 3..…………………………….19 (A4) ABET 4….…………………………..20 (U1) Partial University.……………..21  (U2) University..……………………….22 |
|  |  |  |
| BD013_age | How old were you at the time of your first marriage? |  |
|  |  |  |
| BD014_age | How old were you at the time of your spouse's death? |  |
|  |  |  |
| BD015_age | How old were you when your marriage dissolved, or how many years ago was it? |  |
|  |  |  |
| BD016 | Altogether, how many times have you been married [including your current marriage]? | Once……….…………………………………1 Twice………….……………………………..2 Three times…..…………………………..3 Four or more……………………………..4 |
|  |  |  |
| BD017 | Do you have any children? | YES….............................................1  NO….............................................2 |
|  |  |  |
| BD018 | How many living children do you have? | _____________________Children |
|  |  |  |
| BD019 | How many of your children who were born alive are now deceased? | _____________________Children |
|  |  |  |
| BD020 | What is your oldest child’s name? | ___________________________ |
|  |  |  |
| BD021 | Is [NAME] your son or daughter? | Son….............................................1  Daughter…....................................2 |
|  |  |  |
| BD022 | How old is [NAME] in completed years? | ________________________Years |
|  |  |  |
| BD023 | Where does (your oldest child) live? Does (your oldest child) live within the community, village/city inside state, outside state, or outside country? | Within Agincourt…..……………………1 Within Bushbuckridge………………..2 Within SAF.…..….………………………..3 Outside SAF………..……………………..4 |
|  |  |  |
| BD024 | Now I am going to ask about grandchildren.  Do you have any grandchildren? | YES….............................................1  NO….............................................2 |
|  |  |  |
| BD025 | How many grandchildren do you have? | Number_____________________ |
|  |  |  |
| BD026 | How many of your grandchildren are under age 16 now? | Number_____________________ |
|  |  |  |
| BD027 | How many of your grandchildren reside with you? | Number_____________________ |
|  |  |  |
| BD028 | Do you look after any of these grandchildren? | YES….............................................1  NO ….............................................2 |
|  |  |  |
| BD029 | How many days per week do you care for your grandchildren on average? | ________________ Days per week |
|  |  |  |
| BD030 | Now I will ask about any siblings you have.  Do you have any siblings? | YES….............................................1  NO ….............................................2 |
|  |  |  |
| BD031 | How many of your siblings are still alive? | ______________________Siblings |
|  |  |  |
| BD032 | How many of your living siblings are brothers? | ______________________Siblings |
|  |  |  |
| BD033 | How many of your living siblings are sisters? | ______________________Siblings |
|  |  |  |
| BD034 | Do you belong to a religious denomination? [IWER: ALLOW THE RESPONDENT TO REPLY WITHOUT READING CATEGORIES. CLARIFY AS NEEDED.] | No, none……………………………………1 Christianity (including Roman Catholic, Protestant, Orthodox, Other)…….….……………………………..2 Islam………..………………………………..3 African Traditional..…………………..4  Other…………………..……………………7 |
|  |  |  |
| BD035 | Can you read and write? [IWER: IF RESPONDENT DOES NOT KNOW HOW TO BEST ANSWER, ASK THEM TO READ SENTENCES FROM CARD.] | Able to read only….......................1  Able to write only……...................2  Able to both read and write….....3  Cannot read or write.……………….4 |
|  |  |  |
| BD036 | What is the highest level of education you have attained? | (N) None…….………………………………1 (R) Preschool……….………..…………..2 (A) Sub-A/Gr 1….………………………..3 (B) Sub-B/Gr 2.…………………………..4 (1) Std 1/Gr 3..……………………………5 (2) Std 2/Gr 4……………………………..6 (3) Std 3/Gr 5……………………………..7  (4) Std 4/Gr 6……………………………..8 (5) Std 5/Gr 7……………………………..9 (6) Std 6/Gr 8……………………………10 (7) Std 7/Gr 9……………..…………….11 (8) Std 8/Gr 9….………………………..12 (9) Std 9/Gr 11……….…………………13 (0) Stad 10/Gr 12/Matric…..……..14 (P) Partial Tertiary……………………15 (T) Completed Tertiary……..……..16 (A1) ABET 1..…….………………………17 (A2) ABET 2………………………………18 (A3) ABET 3..…………………………….19 (A4) ABET 4….…………………………..20 (U1) Partial University.……………..21  (U2) University..……………………….22 |
|  |  |  |
| BD037 | When did you go to college? | _______________ _________Year |
|  |  |  |
| BD038 | At what age did you begin formal schooling? |  |
|  |  |  |
| BD039 | At what age did you finish schooling? |  |
|  |  |  |
| BD040 | Now, I am going to ask some questions about your parents and your early life.  Was your mother married or in a union with your father when you were born? | YES................................................1  NO................................................2 |
|  |  |  |
| BD041_insideSFA | Were you born in South Africa? | YES................................................1  NO ................................................2 |
| BD041_town | Where were you born? | Town/village_________________ |
| BD041_municipality | Where were you born? | Municipality__________________ |
|  |  |  |
| BD041_district | Where were you born? | District______________________ |
|  |  |  |
| BD042 | How long have you been living continuously in this (city/town/village) (where you live now)? | Years:________............................1  Lived here all my life.....................2 |
|  |  |  |
| BD043 | Now, I’m going to ask you some questions about your father.  Is he alive? | YES................................................1  NO................................................2 |
|  |  |  |
| BD044 | How old is he? | ________________________Years |
|  |  |  |
| BD045 | How old was he when he died? | ________________________Years |
|  |  |  |
| BD046 | How old were you when your father died? | ________________________Years |
|  |  |  |
| BD047 | Does your father reside with you? | YES...............................................1  NO................................................2 |
|  |  |  |
| BD048 | Does your father live alone or with others? | Live alone……................................1  Live with mother or his partner..2  Live with other children..............3  Live with others……….………..…….4 |
|  |  |  |
| BD049 | Has (Did) he ever attended school? | YES................................................1  NO................................................2 |
|  |  |  |
| BD050 | What was your father’s main occupation? | Farm work………………………………….1 Domestic work………………..…………2 Construction work……………………..3 Security work……………………………..4 Cleaning work……………………………5  Small business owner…………..……6 Mine work………………………………….7 Teacher………………………………………8 Traditional healer……..……………….9 Health sector (formal)………………10 Game farm/game reserve (e.g. ranger)……………………………………..11 Driver……………………………………….12 Skilled worker (e.g. plumber, mechanic, electrician)..…………….13 Cook/ chef/ catering………………..14 Unskilled worker (e.g. general labourer)………………………………….15  Artisan (e.g. carpenter, wood carver, weaver)………..………………………….16  Waiter/ barman……………………….17 Informal selling………..………………18 Small business assistant……………19 Clerical and office work…..……….20 Cattle herder……………………………21 Sewing, hairdressing, baking, brewing……………………………………22 Police, soldier, fireman…………….23 Petrol attendant……………..……….24 Timber, sawmill, poles………..……25  Gardening services…………………..26 Fieldworker - NGO or university…...…………………………...27 Art, craft, photography, fashion design……………………………………...28 Senior administrator, manager, professional……………………………..29 Priest/pastor…………………………….30 Other……………………………………….31 |
|  |  |  |
| BD051 | Now, I’m going to ask you some questions about your mother.  Is she alive? | YES...............................................1  NO................................................2 |
|  |  |  |
| BD052 | How old is she? | ________________________Years |
|  |  |  |
| BD053 | How old was she when she died? | ________________________Years |
|  |  |  |
| BD054 | How old were you when your mother died? | ________________________Years |
|  |  |  |
| BD055 | Does your mother reside with you? | YES...............................................1  NO................................................2 |
|  |  |  |
| BD056 | Does your mother live alone or with others? | Live alone…………...........................1  Live with father or her partner...2  Live with other children…….........3  Live with others……….………..….….4 |
|  |  |  |
| checkBirthDate | [IWER: Go back and check the respondents birth year and month.] |  |
| **Created Variables:** | **Background Section** |  |
| *Variable* | *Label* | *Source* |
|  |  |  |
| c_bd_country | Country of origin | Country of origin (derived from bd041) |
|  |  |  |
| c_bd_educ4 | Education category | Education category (derived from bd036) |
|  |  |  |
| c_bd_mar | Marital status | Marital status (derived from bd003) |
|  |  |  |

**Section: General Health**

| GH001 | Now, I will ask your views about your health. If you are unsure about how to answer a question, please give the best answer you can.  In general, how would you rate your health today? | Very good ………...........................1  Good………………………….................2  Moderate……………….....................3  Bad…………………….…………………….4  Very bad…………………………………..5 |
| --- | --- | --- |
|  |  |  |
| GH002 | Compared to one year ago, how would you rate your health today? | Much worse.……...........................1  Worse….………………..….................2  Same……..…………………………........3  Better..…………………….………….….4  Much better.…………………………..5 |
|  |  |  |
| GH003 | How would you rate your health during your childhood? | Very good ………...........................1  Good………………………….................2  Moderate……………….....................3  Bad…………………….…………………….4  Very bad…………………………………..5 |
|  |  |  |

**Section: Subjective Wellbeing**

| SW001 | All things considered, how satisfied are you with your life as a whole these days? Use a 0 to 10 scale, where 0 is dissatisfied and 10 is satisfied. | ____________________________ |
| --- | --- | --- |
|  |  |  |
| SW002 | Now, please think about yesterday, from the morning until the end of the day. Think about where you were, what you were doing, who you were with, and how you felt.  Did you feel well-rested yesterday? | YES................................................1  NO……...........................................2 |
|  |  |  |
| SW003 | Were you treated with respect all day yesterday? | YES................................................1  NO……...........................................2 |
|  |  |  |
| SW004 | Did you smile or laugh a lot yesterday? | YES................................................1  NO……...........................................2 |
|  |  |  |
| SW005 | Did you learn or do something interesting yesterday? | YES................................................1  NO……...........................................2 |
|  |  |  |
| SW006 | Did you experience the following feelings during A LOT OF THE DAY yesterday? How about...  enjoyment for a lot of the day yesterday? | YES................................................1  NO……...........................................2 |
|  |  |  |
| SW007 | .. physical pain for a lot of the day yesterday? | YES................................................1  NO……...........................................2 |
|  |  |  |
| SW008 | .. worry for a lot of the day yesterday? | YES................................................1  NO……...........................................2 |
|  |  |  |
| SW009 | .. sadness for a lot of the day yesterday? | YES................................................1  NO……...........................................2 |
|  |  |  |
| SW010 | .. stress for a lot of the day yesterday? | YES................................................1  NO……...........................................2 |
|  |  |  |
| SW011 | .. anger for a lot of the day yesterday? | YES................................................1  NO……...........................................2 |
|  |  |  |
| SW012 | .. happiness for a lot of the day yesterday? | YES................................................1  NO……...........................................2 |
|  |  |  |
| SW013 | Please imagine a ladder with steps numbered from 0 at the bottom to 10 at the top. Suppose we say that the top of the ladder represents the best possible life for you and the bottom of the ladder represents the worst possible life for you.  On which step of the ladder would you say you personally feel you stand at this time, assuming that the higher the step the better you feel about your life, and the lower the step the worse you feel about it? Which step comes closest to the way you feel? | ____________________________ |
|  |  |  |
| SW014 | On which step of the ladder would you say you stand relative to others in your village or community? (compared to people in your village) | ____________________________ |
|  |  |  |
| SW015 | Next we would like to ask your opinion about how likely you think various events might be. When I ask a question I'd like for you to give me a number from 0 to 100, where '0' means that you think there is absolutely no chance, and '100' means that you think the event is absolutely sure to happen.  For example, no one can ever be sure about tomorrow's weather, but if you think that rain is very unlikely tomorrow, you might say that there is a low percent chance of rain. If you think there is a very good chance that it will rain tomorrow, you might say that there is a high percent chance of rain. |  |
|  |  |  |
| SW016 | What is the percent chance that you will live to be  85 80 85 90 95 100  [FLSW016] or more? [IWER: INSTRUCTIONS: 0 IS ABSOLUTELY NO CHANCE AND 100 IS ABSOLUTELY CERTAIN.] | ___________________________% |

**Section: Social Networks**

| SN001 | Now I will ask you some questions about your relationships with other people. We will be asking for their names in order to refer to these people during our interviews with you. You can be sure that we will not contact any of these people about you nor share any of your information with them so please feel free to give us their names. Please tell me the names of 6 adults with whom you have been in communication either in person or by phone or by internet in the past 6 months, starting with the person who is most important to you for any reason. These adults may live inside or outside of your household. They could be friends, relatives, acquaintances, neighbors, colleagues, advisors, caretakers, religious leaders, health workers, counselors or anyone else. They must still be alive. |  |
| --- | --- | --- |
|  |  |  |
| SN002 | [IWER: AFTER THE RESPONDENT PROVIDES A NAME, WRITE IT ON THE NAME ROSTER BELOW. MAKE SURE TO WRITE THE NAMES IN THE ORDER WHICH THEY ARE GIVEN TO YOU.] [IWER: AFTER EACH NEW NAME IS GIVEN, PROMPT THE PARTICIPANT: “DID YOU GIVE ME ALL THE NAMES THAT YOU KNOW FOR THIS PERSON?”] THEN, PROMPT THE RESPONDENT FOR THE NEXT NAME BY SAYING, “THANK YOU. PLEASE TELL ME THE NAME OF ANOTHER ADULT WITH WHOM YOU'VE BEEN IN CONTACT OVER THE PAST 6 MONTHS, WHO IS THE NEXT MOST IMPORTANT TO YOU. REMEMBER YOU CAN KNOW THIS PERSON IN ANY WAY, COMMUNICATE IN ANY WAY, AND THEY CAN STAY INSIDE OR OUTSIDE OF YOUR HOUSEHOLD."] [IWER: IF RESPONDENT MENTIONS PEOPLE WITH THE SAME NAME, ASK FOR ANOTHER NAME OR AN IDENTIFYING CHARACTERISTIC ABOUT THIS PERSON, SUCH AS 'OLDER SARAH' OR 'SISTER SARAH'. WRITE DOWN THAT IDENTIFYING CHARACTERISTIC TOO. DO NOT JUST ADD A ‘2’ TO THE NAME BECAUSE THE RESPONDENT WON’T KNOW WHICH SARAH IS 1 AND WHICH SARAH IS 2.] |  |
|  |  |  |
| SN003 | [SOCIAL NETWORK NAME ROSTER] |  |
|  |  |  |
| SNASKPARTNER | Fill variable: If respondent is married and does not mention spouse, spouse auto-populates for contact number 7. |  |
| SN004 | [IWER: IF IT IS OBVIOUS MARK THE CORRECT RESPONSE] Is SN003male or female? | Male…...........................................1  Female..........................................2 |
|  |  |  |
| SN005 | Compared to where you live, where has (this person) currently resided over the past 6 months? Has (this person) generally stayed in…? | Your household............................1  Your village...................................2  Within Agincourt but not your village...........................................3  In South Africa but not Agincourt…………………………........4  In another country…….................5 |
|  |  |  |
| SN006 | Please tell me how you are mainly related to (this person). Is he/she your…? [IWER: READ THROUGH RESPONSE OPTIONS.] | No relationship/do not know each other………………………………….…..1  Acquaintances…….......................2  Married to each other or living together…....................................3  Relatives with each other, but not married to each other………….....4  Friends with each other..............5  Co-workers with each other..…..6  Involved in a club or organization with each other..……………………..7  Other…….………………………………..8 |
|  |  |  |
| SN007 | What is (this person)'s age (in completed years)? [IWER: PROBE. BEST GUESS IS FINE.] | ________________ _______Years |
|  |  |  |
| SN008 | Can (this person) read and write? [IWER: PROBE. BEST GUESS IS FINE.] | YES...............................................1  NO................................................2 |
|  |  |  |
| SN009 | Which of the following answers best describes how often you typically interacted with (this person) in person over the past 6 months? [IWER: READ THROUGH RESPONSE CHOICES. PROBE. BEST GUESS IS FINE.] [IWER: IF A RESPONDENT WANTS TO KNOW WHETHER YOU ARE ASKING ABOUT HOW OFTEN HE OR SHE SEES THE PERSON WHEN THE PERSON IS IN TOWN (IF THEY LIVE IN THE SAME VILLAGE) OR ON AVERAGE OVER A YEAR, THEN ASK R TO THINK ABOUT HOW OFTEN R SEES THIS PERSON OVER THE COURSE OF 6 MONTHS (NOT JUST WHEN THE PERSON IS IN THE VILLAGE).] | Every day or almost every day...1  A few times per week.................2  Once per week.............................3  A few times per month.……........4  Once per month…………..............5  A few times in the past 6 month……………………………………..6  Not at all..………………………………..7 |
|  |  |  |
| SN010 | Which of the following answers best describes how often you typically interacted with (this person) on the phone, by SMS, through email or the internet over the past 6 months? [IWER: READ THROUGH RESPONSE CHOICES. PROBE. BEST GUESS IS FINE.] | Every day or almost every day...1  A few times per week.................2  Once per week.............................3  A few times per month.……........4  Once per month…………..............5  A few times in the past 6 month……………………………………..6  Not at all..………………………………..7 |
|  |  |  |
| SN011 | Which of the following answers best describes how often you typically received emotional support from (this person), such as when you are feeling sad or anxious or upset, over the past 6 months? | Every day or almost every day...1  A few times per week.................2  Once per week.............................3  A few times per month.……........4  Once per month…………..............5  A few times in the past 6 month……………………………………..6  Not at all..………………………………..7 |
|  |  |  |
| SN012 | Which of the following answers best describes how often you typically received physical support from (this person), such as when you have needed help with chores around the house or at work, taking care of yourself or going from one place to another, over the past 6 months? | Every day or almost every day...1  A few times per week.................2  Once per week.............................3  A few times per month.……........4  Once per month…………..............5  A few times in the past 6 month……………………………………..6  Not at all..………………………………..7 |
|  |  |  |
| SN013 | Which of the following answers best describes how often you typically received informational support from (this person), such as receiving advice about important health issues, employment issues, or any other important matters, over the past 6 months? | Every day or almost every day...1  A few times per week.................2  Once per week.............................3  A few times per month.……........4  Once per month…………..............5  A few times in the past 6 month……………………………………..6  Not at all..………………………………..7 |
|  |  |  |
| SN014 | Which of the following answers best describes how often you typically received financial support from (this person), such as borrowing money, receiving food, being given a job or anything else related to money or in-kind transfers, over the past 6 months? | Every day or almost every day...1  A few times per week.................2  Once per week.............................3  A few times per month.……........4  Once per month…………..............5  A few times in the past 6 month……………………………………..6  Not at all..………………………………..7 |
|  |  |  |
| SN015 | Which of the following answers best describes how often (this person) typically physically fought or verbally argued with you or (this person) criticized you in a humiliating way, over the past 6 months? | Every day or almost every day...1  A few times per week.................2  Once per week.............................3  A few times per month.……........4  Once per month…………..............5  A few times in the past 6 month……………………………………..6  Not at all..………………………………..7 |
|  |  |  |
| SN016 | Please tell me which of the following describes the main relationship between (name X) and (name Y). [IWER: READ THROUGH RESPONSE CHOICES 1 THROUGH 8. PROBE. BEST GUESS IS FINE.] | No relationship/do not know each other………………………………….…..1  Acquaintances…….......................2  Married to each other or living together….....................................3  Relatives with each other, but not married to each other………….....4  Friends with each other..............5  Co-workers with each other..…..6  Involved in a club or organization with each other..……………………..7  Other…….………………………………..8 |
|  |  |  |
| SN016_intro | We will now ask you about how the people you mentioned know one another. |  |
|  |  |  |
| SN017 | Which of the following answers best describes how frequently (name X) and (name Y) typically spoke with each other over the past 6 months, either in person or through any other method, according to your perception? | Every day or almost every day…..1  A few times per week..................2  Once per week.............................3  A few times per month.……..........4  Once per month…………................5  A few times in the past 6 months.………………………………..…..6  Not at all..………………………………...7 |
|  |  |  |
| SN018 | I have one more question about these people whom you have mentioned.  Thinking about these people whom we have been talking about right now, with how many of these people have you had a sexual relationship in the past 24 months? | ____________________________ |

**Section: Social Conditions**

| SC001 | Now I will ask you some questions about how often you see your children who do not live with you.  How often do you see (outside child’s name)? | Almost every day………………..……..1  2 -3 times a week………………….…..2  Once a week………………….………….3  Every two weeks……………….………4  Once a month………………….………..5  Once every 3 months…………….….6  Once every 6 months……….……….7  Once a year………..……..………………8  Almost never……………..………………9  Other…………………………………..…..10 |
| --- | --- | --- |
|  |  |  |
| SC002 | How often do you have contact with (outside child’s name) either by phone, text message, mail, or email? | Almost every day………..……………..1  2 -3 times a week………….…………..2  Once a week………………..…………….3  Every two weeks………………..………4  Once a month……………….…………..5  Once every 3 months……………..….6  Once every 6 months……………..….7  Once a year……………….………………8  Almost never…………………..…………9  Other………………………………..……..10 |
|  |  |  |
| SC003 | Are there any adult members of your family (spouse, parents, parents of spouse, siblings, and/or children) who are unable to carry out their basic daily activities? Basic daily activities refer to everyday routines such as eating, putting on clothes, taking a bath, and using the toilet, etc. | YES................................................1  NO................................................2 |
|  |  |  |
| SC004 | Do you take care of any of your family who are unable to carry out their basic daily activities? | YES................................................1  NO................................................2 |
|  |  |  |
| SC005 | How often do you take care of at least one member of your family who is unable to carry out basic daily activities? | Daily…………..….………..………………..1  Several times a week………….……..2  Once a week………………………..…….3  Several times a month……..…..……4  At least once a month………….......5  Not in the last month……..………….6 |
|  |  |  |
| SC006_number | For how many hours did you provide such care during the past week? If it is easier you may give the response in hours per day or days per week. | __________________Hours/Days |
|  |  |  |
| SC006_period | Answer options | Hours per day:________________  Days per week:_______________ |
|  |  |  |
| SC007 | Do you care for any other sick or disabled adults in your household, or your family? | YES................................................1  NO................................................2 |
|  |  |  |
| SC008 | How often do you care for a sick or disabled adult in your household, or your family? | Daily…………………………………………..1  Several times a week…..………..…..2  Once a week…………………………..….3  Several times a month…..……..……4  At least once a month……….……...5  Not in the last month……………..….6 |
|  |  |  |
| SC009_number | For how many hours did you provide such care during the past week? If it is easier you may give the response in hours per day or days per week. | __________________Hours/Days |
|  |  |  |
| SC009_period | Answer options | Hours per day:________________  Days per week:_______________ |
|  |  |  |
| SC010 | Did you spend any time taking care of your grandchildren last year? | YES................................................1  NO................................................2 |
|  |  |  |
| SC011 | For which child’s children did you provide care? | 1 ^ChildName[1] 2 ^ChildName[2] 3 ^ChildName[3] 4 ^ChildName[4] 5 ^ChildName[5] 6 ^ChildName[6] 7 ^ChildName[7] 8 ^ChildName[8] 9 ^ChildName[9] 10 ^ChildName[10] 11 ^ChildName[11] 12 ^ChildName[12] 13 ^ChildName[13] 14 ^ChildName[14] 15 ^ChildName[15] 16 ^ChildName[16] 17 ^ChildName[17] 18 ^ChildName[18] 19 ^ChildName[19] 20 ^ChildName[21] 21 ^ChildName[21] 22 ^ChildName[22] 23 ^ChildName[23] 24 ^ChildName[24] 25 ^ChildName[25] |
|  |  |  |
| SC012 | Approximately how many weeks did you spend in the last year taking care of (child’s name)'s children? [IWER: INSTRUCTIONS: ENTER '1' WEEK IF THE PERIOD IS LESS THAN 7 DAYS.] | Myself:________________Weeks |
|  |  |  |
| SC013 | Approximately how many weeks did your spouse spend in the last year taking care of (child’s name)'s children? [IWER: INSTRUCTIONS: ENTER '1' WEEK IF THE PERIOD IS LESS THAN 7 DAYS, ENTER '0' IF THEY DO NOT TAKE CARE OF THE CHILDREN.] | My spouse:_____________Weeks |
|  |  |  |
| SC014 | Approximately how many days per week in those weeks did you spend in the last year taking care of this child’s children? | Myself:___________Days per week |
|  |  |  |
| SC015 | Approximately how many days per week in those weeks did your spouse/partner spend in the last year taking care of this child’s children? [IWER: INSTRUCTIONS: ENTER '0' IF THEY DO NOT TAKE CARE OF THE CHILDREN.] | My spouse:________Days per week |
|  |  |  |
| SC016 | Did you (or your spouse) take care of your parents (or parents-in-law) during the last year in assisting them in their daily activities or other activities (e.g., household chores, meal preparation, laundry, going out, grocery shopping, financial management, etc.)? | YES................................................1NO..............................................2 |
|  |  |  |
| SC017_father |  | Your father:_____________Weeks |
|  |  |  |
| SC017_fatherinlaw |  | Your father-in-law:_______Weeks |
|  |  |  |
| SC017_intro | Approximately how many weeks did you yourself spend last year taking care of your parents (or parents-in-law)? |  |
|  |  |  |
| SC017_mother |  | Your mother:___________Weeks |
|  |  |  |
| SC017_motherinlaw |  | Your mother-in-law:______Weeks |
|  |  |  |
| SC018_father |  | Your father:______Days per week |
|  |  |  |
| SC018_fatherinlaw |  | Your father-in-law:_______Days per week |
|  |  |  |
| SC018_intro | Approximately how many days per week did you yourself spend last year taking care of your parents (or parents-in-law)? |  |
|  |  |  |
| SC018_mother |  | Your mother:_________Days per week |
|  |  |  |
| SC018_motherinlaw |  | Your mother-in-law:_______Days per week |
|  |  |  |
| SC019_father |  | Your father:_____________Weeks |
|  |  |  |
| SC019_fatherinlaw |  | Your father-in-law: ______Weeks |
|  |  |  |
| SC019_intro | Approximately how many weeks did your spouse spend last year taking care of your parents or parents-in-law? |  |
|  |  |  |
| SC019_mother |  | Your mother:___________Weeks |
|  |  |  |
| SC019_motherinlaw |  | Your mother-in-law: _______Weeks |
|  |  | Your father:_______Days per week |
| SC020_father |  |  |
|  |  |  |
| SC020_fatherinlaw |  | Your father-in-law:______Hours per week |
|  |  |  |
| SC020_intro | Approximately how many days per week did your spouse spend last year taking care of your parents or parents-in-law? |  |
|  |  |  |
| SC020_mother |  | Your mother:______Days per week |
|  |  |  |
| SC020_motherinlaw |  | Your mother-in-law:_____Days per week |
|  |  |  |
| SC021 | The next few questions ask about what help or assistance you, as a caregiver, received from other people or groups to assist you in providing care for anyone in your household.  What kind of help have you, as a caregiver, received? [IWER: INSTRUCTIONS: READ EACH OPTION AND CHECK ALL THAT APPLY] | Financial, such as cash, paying for bills, fees, food or medicines, clothing or other provisions….……1  Emotional, like social support, counseling, time with friends…………………………..……..……2 Health, including providing health care, administering medicines, changing bandages, arranging health care providers…………………………….…….3 Physical including household chores, transportation………………………..….4 Personal care, help with bathing, eating, dressing, toileting, moving around……………………………………….5 Other………………………………..……….6 None……………………………………..…..7 |
|  |  |  |
| SC022 | Who provided this help or assistance? [IWER: CIRCLE ALL ANSWERS THAT THE RESPONDENT SPONTANEOUSLY MENTIONS. 'ANYONE ELSE?'.] | Spouse………………………..…………....1 Family inside the household (not spouse)………………………..…..……….2 Family outside household…..…..…3 Friend(s)……………………..……………..4 Neighbours/community…….………5 Government…………………………..….6  Church……………………………..………..7 NGOs…………………………………..…….8 Other………………..……………………….9  None………………………….…………….10 |
|  |  |  |

**Section: Performance Tests**

| PT001 | Normal walk [IWER: REFER TO SHOWCARDS] Did respondent complete the walk at usual pace? | YES……………………….…………………..1  NO,REFUSED………….…..……………..2  NO, CANNOT WALK< EVEN WITH SUPPORT……………………………………3 |
| --- | --- | --- |
|  |  |  |
| PT002 | Normal walk. Time at 2.5 meters: | _____________________Seconds |
|  |  |  |
| PT003 | Normal walk. Did respondent complete the walk at rapid pace? | YES…………………..………………………..1  NO, REFUSED/UNABLE..……………..2  _____________________Seconds |
|  |  |  |
| PT004 | Normal walk. Time at 5.0 meters: | _____________________Seconds |
|  |  |  |
| PT005 | Semi-tandem. Before we begin, do you have any problems from recent surgery, injury or other health conditions that might prevent you from standing up from a chair and balancing? | YES................................................1  NO................................................2 |
|  |  |  |
| PT006 | Semi-tandem. Did the respondent complete the semi-tandem stand? | YES................................................1  NO ................................................2 |
|  |  |  |
| PT007 | Semi-tandem. Why didn’t Respondent complete the semi-tandem stand? [IWER: INSTRUCTIONS: CHECK ALL THAT APPLY.] | Respondent felt it would not be safe……………………………………………1  Interviewer felt it would not be safe………………………………………..….2 Respondent refused or was not willing to complete test....………….3 Respondent tried but was unable to complete test………………...........4 Respondent did not understand the instructions……………..………………..5 Respondent had surgery, injury or other health condition that prevented R from standing……………………………………..6 No suitable space……………………...7 Problem with equipment or supplies………………………………….….8 Other…………………………………………9 |
|  |  |  |
| PT008 | Did Respondent hold semi-tandem stand for a full 10 seconds without stepping out of place or grabbing hold of anything? | YES…………..………………………………..1 NO………………………………………….….2 NO, CHOOSE NOT TO DO IT………..3 NO, TRIED BUT NOT ABLE…………..4 |
|  |  |  |
| PT009 | How long did Respondent hold semi-tandem stand in seconds? | _____________________Seconds |
|  |  |  |
| PT010 | Did Respondent use any compensatory movements of his/her trunk, arms or legs to steady him/herself during semi-tandem stand? | YES................................................1  NO ................................................2 |
|  |  |  |
| PT011 | Respondent should do the 30 or 60 seconds full-tandem balance measurement. |  |
|  |  |  |
| PT012 | Did the respondent complete the full-tandem stand? | YES................................................1  NO ................................................2 |
|  |  |  |
| PT013 | Why didn’t Respondent complete the full-tandem stand? [IWER: INSTRUCTIONS: CHECK ALL THAT APPLY.] | Respondent felt it would not be safe……………………………………………1  Interviewer felt it would not be safe………………………………………..….2 Respondent refused or was not willing to complete test....………….3 Respondent tried but was unable to complete test………………...........4 Respondent did not understand the instructions……………..………………..5 Respondent had surgery, injury or other health condition that prevented R from standing……………………………………..6 No suitable space……………………...7 Problem with equipment or supplies………………………………….….8 Other…………………………………………9 |
|  |  |  |
| PT014 | Did Respondent hold full-tandem stand for a full 30 or 60 seconds without stepping out of place or grabbing hold of anything? | YES…………..………………………………..1 NO………………………………………….….2 NO, CHOOSE NOT TO DO IT………..3 NO, TRIED BUT NOT ABLE…………..4 |
|  |  |  |
| PT015 | How long did Respondent hold full-tandem stand in seconds? | _____________________Seconds |
|  |  |  |
| PT016 | Did Respondent use any compensatory movements of his/her trunk, arms or legs to steady him/herself during full-tandem stand? | YES................................................1  NO ................................................2 |
|  |  |  |
| PT017 | Record the type of floor surface that the balance measures were conducted on. | Linoleum/tile/wood……………………1  Carpet………………………………………..2  Clay……………………………………………3  Concrete…………………………………….4  Not sure……………………………………..5  Other………………………………………….6 |
|  |  |  |
| PT018 | How compliant was Respondent during the balance measurements? | Respondent was fully compliant…1 Respondent was prevented from fully complying due to illness, pain, or other symptoms or discomforts……………………………….2 Respondent did not appear to be fully compliant, but no obvious reason for this……………………………3 |
|  |  |  |
| PT019 | Did the respondent complete the side-by-side stand? | YES................................................1  NO ................................................2 |
|  |  |  |
| PT020 | Why didn’t Respondent complete the side-by-side stand? [IWER: INSTRUCTIONS: CHECK ALL THAT APPLY.] | Respondent felt it would not be safe……………………………………………1  Interviewer felt it would not be safe………………………………………..….2 Respondent refused or was not willing to complete test....………….3 Respondent tried but was unable to complete test………………...........4 Respondent did not understand the instructions……………..………………..5 Respondent had surgery, injury or other health condition that prevented R from standing……………………………………..6 No suitable space……………………...7 Problem with equipment or supplies………………………………….….8 Other…………………………………………9 |
|  |  |  |
| PT021 | Did Respondent hold side-by-side stand for a full 10 seconds without stepping out of place or grabbing hold of anything? | YES…………..………………………………..1 NO………………………………………….…2 NO, CHOSE NOT TO DO IT……..…..3 NO, TRIED BUT NOT ABLE………....4 |
|  |  |  |
| PT022 | How long did Respondent hold side-by-side stand in seconds? | YES................................................1  NO ................................................2 |
|  |  |  |
| PT023 | Did Respondent use any compensatory movements of his/her trunk, arms or legs to steady him/herself during side-by-side stand? | YES................................................1  NO ................................................2 |
|  |  |  |
| PT024 | Record the type of floor surface that the balance measures were conducted on. | Linoleum/tile/wood……………………1  Carpet………………………………………..2  Clay……………………………………………3  Concrete…………………………………….4  Not sure……………………………………..5  Other………………………………………….6 |
|  |  |  |
| PT025 | How compliant was Respondent during the balance measurements? | Respondent was fully compliant…1 Respondent was prevented from fully complying due to illness, pain, or other symptoms or discomforts……………………………….2 Respondent did not appear to be fully compliant, but no obvious reason for this……………………………3 |
|  |  |  |
| PT032_intro | [IWER: ASK THE RESPONDENT TO HOLD THE TAPE MEASURE AT THEIR HIPS AND WALK AROUND THE SUBJECT. INSERT THE END OF THE TAPE INTO THE SLOT ON THE DEVICE AND RETRACT ANY SLACK IN THE TAPE. ENSURE THE TAPE IS HORIZONTAL AND CORRECTLY POSITIONED AT THE WIDEST PART OF PARTICIPANT'S HIPS, AROUND THEIR REAR END. MEASURE AND RECORD RESPONDENTS HIP CIRCUMFERENCE IN CENTIMETERS] |  |
|  |  |  |
| PT032  *[use c_pt_hip]* | [IWER: MEASURE AND ENTER RESPONDENTS HIP CIRCUMFERENCE IN CENTIMETERS] | __________________Centimeters |
|  |  |  |
| PT032_check  *[use c_pt_hip]* | [IWER: CHECK HIP ENTRY] | __________________Centimeters |
|  |  |  |
| PT032_reenter  *[use c_pt_hip]* | [IWER: RE-ENTER AND RECORD RESPONDENTS HIP CIRCUMFERENCE IN CENTIMETERS] | __________________Centimeters |
|  |  |  |
| PT033_intro | Now I would like to measure the circumference of your waist and hips. [IWER: ENSURE THE SUBJECTS IS NOT WEARING ANY BULKY OVERGARMENTS AND THAT THEIR POCKETS ARE EMPTY. ASK THE RESPONDENT TO HOLD THE TAPE MEASURE AT THEIR NAVEL AND WALK AROUND THE SUBJECT. INSERT THE END OF TAPE INTO THE SLOT ON THE DEVICE AND RETRACT ANY SLACK IN THE TAPE. ENSURE THE TAPE IS HORIZONTAL AND CORRECTLY POSITIONED AT THE PARTICIPANT'S NAVEL. RECORD RESPONDENTS WAIST CIRCUMFERENCE IN CENTIMETERS] |  |
|  |  |  |
| PT033  *[use c_pt_waist]* | [IWER: MEASURE AND RECORD RESPONDENTS WAIST CIRCUMFERENCE IN CENTIMETERS] | __________________Centimeters |
|  |  |  |
| PT033_check  *[use c_pt_waist]* | [IWER: CHECK WAIST MEASURE] | __________________Centimeters |
|  |  |  |
| PT033_reenter  *[use c_pt_waist]* | [IWER: RE-ENTER RESPONDENTS WAIST CIRCUMFERENCE IN CENTIMETERS] | __________________Centimeters |
|  |  |  |
| PT040 | Have you had any surgery on your left arm, hand or wrist in the last 3 months OR arthritis or pain in your left hand or wrist? | YES................................................1  NO ................................................2 |
|  |  |  |
| PT041 | Have you had any surgery on your right arm, hand or wrist in the last 3 months OR arthritis or pain in your right hand or wrist? | YES................................................1  NO ................................................2 |
|  |  |  |
| PT042 | Which hand do you consider your dominant hand? [IWER: INSTRUCTIONS: IF A RESPONDENT IS AMBIDEXTROUS, THE HAND THAT IS USED FOR SIGNING/WRITING IS CONSIDERED THE DOMINANT HAND.] | LEFT……………………………………………1  RIGHT…………………………………………2  USE BOTH THE SAME………………….3 |
|  |  |  |
| PT043  *[use c_pt043]* | First test, left hand | _________________________Kg |
|  |  |  |
| PT044  *[use c_pt044]* | Second test, left hand | _________________________Kg |
|  |  |  |
| PT045  *[use c_pt045]* | First test, right hand | _________________________Kg |
|  |  |  |
| PT046  *[use c_pt046]* | Second test, right hand | _________________________Kg |
|  |  |  |
| **Created Variables:** | **Performance Tests Section** |  |
| *Variable* | *Label* | *Variable* |
|  |  |  |
| c_pt_hip | Hip measurement (cm) | Hip measure (cm): Measures taken from pt032, pt032_reenter, and comments data. |
|  |  |  |
| c_pt_waist | Waist measurement (cm) | Waist measure (cm): Measures taken from pt033, pt033_reenter, and comments data. |
|  |  |  |
| c_pt_whratio | Waist/hip ratio | Waist/hip ratio (if hip and waist not out of range) |
|  |  |  |
| c_pt043 | Grip strength first LEFT | Grip strength (kg) first LEFT (cleaned from comments data) |
|  |  |  |
| c_pt044 | Grip strength second LEFT | Grip strength (kg) second LEFT (cleaned from comments data) |
|  |  |  |
| c_pt045 | Grip strength first RIGHT | Grip strength (kg) first RIGHT (cleaned from comments data) |
|  |  |  |
| c_pt046 | Grip strength second RIGHT | Grip strength (kg) second RIGHT (cleaned from comments data) |

|  |  |  |
| --- | --- | --- |
| fl_c_pt_hip | Flag for c_pt_hip | Cleaned hip measure out of range (<50 or >160 cm) |
|  |  |  |
| fl_c_pt_waist | Flag for c_pt_waist | Cleaned waist measure out of range (<50 or >160 cm) |

**Section: Employment & Pensions**

| EP001 | Now I have some questions about work.  What would be the best description of your current work status? [IWER: CIRCLE ALL THAT APPLY] | Unable to Work (Disabled)………..1 Unemployed……………………………..2 Homemaker………………………………3 Working Full Time (working 35 or more hours per week)……………….4 Working Part Time (working less than 35 hours per week)…………...5 Retired………………………………………6  Sick or other leave…………………….7  Other…………………………………………8 |
| --- | --- | --- |
|  |  |  |
| EP002 | In the last month (past 30 days), did you work for a wage or salary? | YES................................................1  NO ................................................2 |
|  |  |  |
| EP003 | In the last month (past 30 days), did you work without being paid? | YES................................................1  NO ................................................2 |
|  |  |  |
| EP004 | Now I'd like to ask you about your current jobs. How many jobs do you currently hold? | ________________________Jobs |
|  |  |  |
| EP005 | Starting with your main job, or the job where you earn the most:  For this job, are you… | Self-employed……………………………1 Working for someone else…………2 |
|  |  |  |
| EP006 | What type of job is this? | Farm work………………………………….1 Domestic work…………………………..2 Construction work……………………..3 Security work…………………………….4 Cleaning work…………………………….5 Small business owner…………………6 Mine work………………………………….7 Teacher………………………………………8 Traditional healer……………………….9 Health sector (formal)………………10 Game farm/game reserve (e.g. ranger)……………………………………..11 Driver……………………………………….12 Skilled worker (e.g. plumber, mechanic, electrician)………………13 Cook/ chef/ catering…………………14 Unskilled worker (e.g. general labourer)………………………………….15  Artisan (e.g. carpenter, wood carver, weaver)………………………..16 Waiter/ barman……………………….17 Informal selling………………………..18 Small business assistant……………19 Clerical and office work…………...20 Cattle herder……………………………21 Sewing, hairdressing, baking, brewing……………………………………22 Police, soldier, fireman…………….23 Petrol attendant………………………24 Timber, sawmill, poles …………….25 Gardening services ………………….26 Fieldworker - NGO or university…………………………………27 Art, craft, photography, fashion design………………………………………28 Senior administrator, manager, professional……………………………..29 Priest/pastor…………………………….30 Other……………………………………….31 |
|  |  |  |
| EP007 | How many hours per week do you work on this job? | ______Number of hours per week |
|  |  |  |
| EP009 | How is your wage rate for this job primarily paid? | Hourly………………………………………..1  Daily…………………………………………..2 Weekly……………………………………….3 Monthly……………………………………..4 Fortnight (every 2 weeks)…………..5 |
|  |  |  |
| EP010 | What is your wage rate for this job before taxes and deductions? [IWER: MAKE SURE THAT WAGE RATE CORRESPONDS TO PREVIOUS COLUMN] | _______________________Rand |
|  |  |  |
| EP011 | Is income tax paid on your salary for this job? | YES................................................1  NO ................................................2 |
|  |  |  |
| EP012 | How many days of work did you miss in the last 30 days due to health problems? | ________________________Days |
|  |  |  |
| EP013 | Have you ever worked underground in mining? | YES................................................1  NO ................................................2 |
|  |  |  |
| EP014_months | For how long did you work underground in mining? | _____________________Months |
|  |  |  |
| EP014_years |  | _______________________Years |
|  |  |  |
| EP015 | Are you currently looking for work? | YES................................................1  NO ................................................2 |
|  |  |  |
| EP016 | Have you received any income from these sources in the past 12 months? [IWER: READ RESPONSES, CHECK ALL THAT APPLY.] | Public Unemployment benefit……1  Insurance……………………………………2  None………………………………………….3 |
|  |  |  |
| EP017 | Before taxes and deductions, what was the last monthly payment? | _______________________Rand |
|  |  |  |
| EP018 | In what month and year did you become disabled? | ______Month_____Year……….…..1  Since birth………………………………….2 |
|  |  |  |
| EP019 | How did you become disabled? | Work-related disability………………1 Non-work related accidents (e.g., falls, road traffic injuries)……………2 Other………………………………………….3 |
|  |  |  |
| EP020 | Have you received any income from any of these sources in the past 12 months? [IWER: READ RESPONSES. CHECK ALL THAT APPLY.] | Public Disability Insurance………….1 Public Invalidity or incapacity pension………………………………………2 Private Disability or Invalidity insurance……………………………………3 None………………………………………….4 |
|  |  |  |
| EP021 | Before taxes and deductions, what was the last monthly payment? | _______________________Rand |
|  |  |  |
| EP022 | Did you ever work? | YES................................................1  NO ................................................2 |
|  |  |  |
| EP023 | What age were you when you stopped working? | Age :________________Years old |
|  |  |  |
| EP024_amount | What was your wage rate, before taxes and deductions, when you last worked [IWER: INSTRUCTIONS: IF AMOUNT PER HOUR, ENTER BOTH RAND AND CENTS. ENTER AMOUNT IN RAND. DO NOT PROBE IF RESPONDENT ANSWERS “DON’T KNOW” OR REFUSES TO ANSWER.] | _______________________Rand |
|  |  |  |
| EP024_period | Per: | Hour…………………………………………..1  Week…………………………………………2  Month………………………………………..3  Year……………………………………………4 |
|  |  |  |
| EP025 | In your main job, what kind of industry or business did you work in -- that is, what did your company do or make at the place where you worked? | Farm work………………………………….1 Domestic work…………………………..2 Construction work……………………..3 Security work…………………………….4 Cleaning work…………………………….5 Small business owner…………………6 Mine work………………………………….7 Teacher………………………………………8 Traditional healer……………………….9 Health sector (formal)………………10 Game farm/game reserve (e.g. ranger)……………………………………..11 Driver……………………………………….12 Skilled worker (e.g. plumber, mechanic, electrician)………………13 Cook/ chef/ catering…………………14 Unskilled worker (e.g. general labourer)………………………………….15  Artisan (e.g. carpenter, wood carver, weaver)………………………..16 Waiter/ barman……………………….17 Informal selling………………………..18 Small business assistant……………19 Clerical and office work…………...20 Cattle herder……………………………21 Sewing, hairdressing, baking, brewing……………………………………22 Police, soldier, fireman…………….23 Petrol attendant………………………24 Timber, sawmill, poles …………….25 Gardening services ………………….26 Fieldworker - NGO or university…………………………………27 Art, craft, photography, fashion design………………………………………28 Senior administrator, manager, professional……………………………..29 Priest/pastor…………………………….30 Other……………………………………….31  Unknown………………………………….32 |
|  |  |  |
| EP026 | Did you make any profit? | YES................................................1  NO ................................................2 |
|  |  |  |
| EP027 | How much did you make in the last year? | ________________________Rand |
|  |  |  |
| **Created Variables:** | **Employment & Pensions Section** |  |
| *Variable* | *Label* | *Source* |
|  |  |  |
| c_ep_employed | Employment status | Categorized employment status from ep001 and ep001_other |

**Section: Earnings Benefits**

| EB001 | The following are fringe benefits which may be provided by a company/employer. Please answer if the following are provided by any of your current jobs. [IWER: CHECK ALL THAT APPLY] | Free lunch………………………………….1 Free breakfast……………………………2  Free dinner………………………………..3 Electricity…………………………………..4 Telephone………………………………….5 Meal cash subsidizations…………...6 Transportation cash subsidizations…………………………….7 Free accommodation…………………8 Subsidization of housing…………….9 Other subsidies………………………..10 None………………………………………..11 |
| --- | --- | --- |
|  |  |  |
| EB002 | How much are the (estimated) value of all these subsidies per month? | ________________________Rand |
|  |  |  |
| EB003 | Do you currently receive any pension based on employment or old-age or survivor-benefits? | YES................................................1  NO ................................................2 |
|  |  |  |
| EB004 | Do you receive any income from any of the following? [IWER: CHECK ALL THAT APPLY] | Public Old Age Pension………………1 Public Early Retirement/ Pre- Retirement Pension……………………2 Public Disability Insurance………….3 Public Survivor Pension………………4 Private Old Age Pension…………….5 Private Early Retirement/ Pre- Retirement Pension……………………6 Private Survivor Pension…………….7 |
|  |  |  |
| EB005 | Before taxes, what was the last monthly payment that you received from (this benefit)? | ________________________Rand |
|  |  |  |
| EB006 | Have you ever received a lump sum as a pension or disability payment? | YES................................................1  NO ................................................2 |
|  |  |  |
| EB007 | What age were you when you got it? | _____________________Years old |
|  |  |  |
| EB008 | How much was it? [IWER: LARGEST IF MULTIPLE] | ________________________Rand |
|  |  |  |
| EB010 | Have you contributed to the provident fund? | YES................................................1  NO ................................................2 |
|  |  |  |
| EB011 | How many years of contributions do you have? | ________________________Years |

**Section: Food Security**

| FS001 | In the past 1 year how often was there no food at all in your household because you lacked money to purchase more? | Never………………………………………..1 Rarely (once or twice)……………….2 Sometimes (3-10 times)…………….3  Often (more than 10 times)……….4 |
| --- | --- | --- |
|  |  |  |
| FS002 | In the past 1 year how often did you or any household members go to sleep at night hungry because there was not enough food? | Never………………………………………..1 Rarely (once or twice)……………….2 Sometimes (3-10 times)…………….3  Often (more than 10 times)……….4 |
|  |  |  |
| FS003 | In the past 1 year how often did you or any household members go a whole day without eating anything because there was not enough food? | Never………………………………………..1 Rarely (once or twice)……………….2 Sometimes (3-10 times)…………….3  Often (more than 10 times)……….4 |

**Section: Health Service Utilization**

| HU001 | Now I will ask you about health care services you received recently.  In the past 12 months, have you been admitted to (i.e., have you slept in) a hospital? | YES................................................1  NO ................................................2 |
| --- | --- | --- |
|  |  |  |
| HU002 | In the past 3 months, have you visited any of the places below? [IWER: SELECT ALL THAT APPLY] | A public primary care clinic………..2 A private doctor or private clinic…………………………………………..3 Isangoma (diviner)……………………..4 A faith healer……………………………..5  None………………………………………..99 |
|  |  |  |
| HU003_intro | On your last visit for this health care, how much did you pay out-of-pocket for ... |  |
|  |  |  |
| HU003_accomodation | Accommodation | ________________________Rand |
|  |  |  |
| HU003_food | Food | ________________________Rand |
|  |  |  |
| HU003_hospitalfees | Hospital/clinic fees | ________________________Rand |
|  |  |  |
| HU003_medicines | Medicines | ________________________Rand |
|  |  |  |
| HU003_phoning | Phoning or sms’ing | ________________________Rand |
|  |  |  |
| HU003_transport | Transport | ________________________Rand |
|  |  |  |
| HU003_visits_12months | How many visits in the last 12 months? | ________________________Visits |
|  |  |  |
| HU003_visits_3months | How many visits in the last 3 months? | ________________________Visits |

**Section: Cognition**

| CN001 | Now I am going to ask you some questions about cognition.  How would you rate your memory at the present time? Would you say it is excellent, very good, good, fair or poor? | Excellent…………………………………….1  Very good…………………………………..2  Good………………………………………….3  Fair…………………………………………….4  Poor…………………………………………..5 |
| --- | --- | --- |
|  |  |  |
| CN002 | Overall in the last 30 days, how much difficulty did you have with concentrating or remembering things? | None………………………………………….1  Mild……………………………………………2  Moderate…………………………………..3  Severe………………………………………..4  Extreme/cannot do…………………….5 |
|  |  |  |
| CN003 | Overall in the last 30 days, how much difficulty did you have in learning a new task (for example, learning how to get to a new place, learning a new game, learning a new recipe)? | None………………………………………….1  Mild……………………………………………2  Moderate…………………………………..3  Severe………………………………………..4  Extreme/cannot do…………………….5 |
|  |  |  |
| CN004 | We are going to read a list consisting of 10 words and we would like you to memorize as many as you can. We deliberately made the list long to make it difficult for anyone to memorize all of the words; most people will only remember a few of them. Please listen carefully as we read the list because we cannot repeat it. When we finish reading the list, we will ask you to recall and tell us as many words as you can remember, and they don’t have to be in the order that you heard them. Is this explanation clear? [IWER: IF NO: EXPLAIN] | YES................................................1  NO ................................................2 |
| CN005 | Randomized variable: [WHICH WORD LIST] |  |
| CN006 | [IWER: READ THE LIST SLOWLY, WITH AN INTERVAL OF ABOUT 2 SECONDS BETWEEN EACH WORD. DO NOT ALLOW PROXY ANSWERS.] Rice  River  Doctor  Clothes  Egg  Cat  Bowl  Child  Hand  Book |  |
|  |  |  |
| CN007 | Now please let us know the words you are able to recall. [IWER: INSTRUCTIONS: GIVE RESPONDENT ENOUGH TIME TO RECALL, APPROXIMATELY UP TO 2 MINUTES.] [IWER: INSTRUCTIONS: CIRCLE ALL THE WORDS MENTIONED BY THE RESPONDENT ON THE COLUMN.] | Rice…………………………………………..1 River………………………………………….2 Doctor……………………………………….3 Clothes………………………………………4 Egg…………………………………………….5Cat…………………………………………….6 Bowl………………………………………….7 Child………………………………………….8 Hand………………………………………….9 Book………………………………………..10  None recalled………………………….88 |
|  |  |  |
| CN008 | [IWER: INSTRUCTIONS: READ THE SAME LIST ONCE MORE, UP TO 3 TIMES, AND THEN GO ON. IF RESPONDENT DOES NOT RECALL ANY OF THE WORDS, ASSURE THEM THAT IT IS OK SO THAT RESPONDENT WILL FEEL COMFORTABLE/AT EASE.]  Rice  River  Doctor  Clothes  Egg  Cat  Bowl  Child  Hand  Book |  |
|  |  |  |
| CN009 | Try to remember the words I just read to you. I'll ask you to recall them later.  Please count from 1 to 20 | Counted correctly 1 to 20…………..1 Cannot count 1-20……………………..2 Counted incorrectly 1-20……………3 |
|  |  |  |
| CN010 | [IWER: MAKE SURE RESPONDENT HAS PENCIL AND PAPER READY FOR WRITING DOWN THE NUMBERS. REPEAT INSTRUCTIONS FOR THE RESPONDENT IF NECESSARY. ALLOW ENOUGH TIME WHEN READING NUMBERS FOR RESPONDENT TO WRITE DOWN SEQUENCE.] Next, I'm going to read you several numbers and I'd like you to write them down from left to right. There will be a blank number in the series that I read to you. Draw a dash or short blank line when I say “blank.” Then look at the pattern of numbers. Based on this pattern, tell me what number goes in the blank. Sometimes the blank will be at the end of the series, and sometimes the blank will be in the beginning or in the middle. For example, if I said the numbers ‘1. . . 2. . . BLANK. . .4’ then what number would go in the blank? [IWER: THE CORRECT RESPONSE IS 3. IF RESPONDENT DOES NOT GIVE THE CORRECT RESPONSE “3” THEN SAY: “THE ANSWER WE WERE LOOKING FOR IS 3.” PROBE IF NEEDED TO CHECK THAT THE RESPONDENT UNDERSTANDS THE TASK, BY ASKING: “DO YOU UNDERSTAND THE DIRECTIONS FOR THIS TASK?”] |  |
|  |  |  |
| CN011 | I’m going to read you a series of numbers. There will be a blank number in the series that I read to you. I would like you to write down the numbers from left to right and then tell me what number goes in the blank based on the pattern of numbers. 2. . . 4. . . 6. . .BLANK [IWER: NOW PLEASE LOOK AT THE NUMBER YOU JUST WROTE DOWN AND TELL ME THE NUMBER THAT GOES IN THE BLANK.] [IWER: THE SEQUENCE IS 2 4 6 8. 8 IS THE ANSWER WE WERE LOOKING FOR BECAUSE, IN THIS EXAMPLE, THE NUMBERS INCREASE BY 2.] | 8…………………………………………………1 Any number other than 8…………..2 Does not know answer………………3 Respondent does not understand instructions………………………………..4 |
|  |  |  |
| CN012 | A little while ago, I read you a list of words and you repeated the ones you could remember. Please tell me any of the words that you remember now. [IWER: ANSWERS ARE DISPLAYED ONLY FOR INTERVIEWER. PLEASE DO NOT SHOW THE SCREEN TO RESPONDENT.] [IWER: CHECK ALL THE WORDS MENTIONED BY THE RESPONDENT AND LIST ALL OF THE WORDS MENTIONED WHICH ARE NOT ON THE LIST] | Rice…………………………………………..1 River………………………………………….2 Doctor……………………………………….3 Clothes………………………………………4 Egg…………………………………………….5Cat…………………………………………….6 Bowl………………………………………….7 Child………………………………………….8 Hand………………………………………….9 Book………………………………………..10  Not on the list………………………….11  Not on the list………………………….12  Not on the list………………………….13  Not on the list………………………….14  Not on the list………………………….15  None recalled………………………….16 |
|  |  |  |
| CN013 | [IWER: HOW OFTEN DID THE RESPONDENT RECEIVE ASSISTANCE IN ANSWERING THIS SECTION?] | Never………………………………………..1  A few times……………………………….2  Most or all of the time……………….3 |
|  |  |  |
| CN014 | What is the year we are in now? | Correct……………………………………….1  Incorrect…………………………………….2 |
|  |  |  |
| CN015 | Can you tell me the month? | Correct……………………………………….1  Incorrect…………………………………….2 |
|  |  |  |
| CN016 | What is the date today? (what day of the month is it today) | Correct……………………………………….1  Incorrect…………………………………….2 |
|  |  |  |
| CN017 | Who is the current president? | Correct……………………………………….1  Incorrect…………………………………….2 |

**Section: Proxy Cognition**

| PC001 | Part of this study is concerned with people's memory, and ability to think about things. First, how would you rate (respondent’s name) 's memory at the present time? Would you say it is excellent, very good, good, fair, or poor? | Excellent…………………………………….1  Very good…………………………………..2  Good………………………………………….3  Fair…………………………………………….4  Poor…………………………………………..5 |
| --- | --- | --- |
|  |  |  |
| PC002 | Compared to two years ago, would you say (respondent’s name)'s memory is better now, about the same, or worse now than it was then? | Better…………………………………………1  Same………………………………………….2  Worse………………………………………..3 |
|  |  |  |
| PC003 | Now, I’m going to ask you a few questions about (respondent’s name).  Now, thinking about some current behaviors, does [she/he] ever get lost in a familiar environment? | YES................................................1  NO ................................................2 |
|  |  |  |
| PC004 | Does [she/he] ever wander off and not return by [herself/himself]? | YES................................................1  NO ................................................2 |
|  |  |  |
| PC005 | Can [she/he] be left alone for an hour or so? | YES................................................1  NO ................................................2 |
|  |  |  |
| PC006 | Does [she/he] ever see or hear things that are not really there? | YES................................................1  NO ................................................2 |

**Section: Perceptions Social Environment**

| PS001 | Now I have some questions about the area where you live.  In general, how much do you like your village? Do you like it a lot, like it, dislike it, dislike it a lot, or don’t care? | Like it a lot………………………………….1  Like it…………………………………………2  Dislike it……………………………………..3  Dislike it a lot……………………………..4 |
| --- | --- | --- |
|  |  |  |
| PS002 | How well are houses in the village maintained? | Very well……………………………………1  Well……………………………………………2  Not very well………………………………3 |
|  |  |  |
| PS003 | How well are public spaces in your village maintained? (such as: marketplaces, soccer fields, public meeting areas, common green space) | Very well……………………………………1  Well……………………………………………2  Not very well………………………………3 |
|  |  |  |
| PS004 | In general, how easy is it for people to walk in your village? | Very easy……………………………………1  Easy……………………………………………2  Not very easy..……………………………3 |
|  |  |  |
| PS005 | I am going to read some statements about things that most people in your village may or may not do. For each of these statements please tell me whether you strongly agree, agree, disagree, or strongly disagree.  If there is a problem in the village, most people in this village work together to deal with it. | Strongly agree (cohesion)…………..1 Agree…………………………………………2 Disagree…………………………………….3 Strongly disagree……………………….4 |
|  |  |  |
| PS006 | Most people in this village are willing to help their neighbors. | Strongly agree (cohesion)…………..1 Agree…………………………………………2 Disagree…………………………………….3 Strongly disagree……………………….4 |
|  |  |  |
| PS007 | Most people in this village can be trusted. | Strongly agree (cohesion)…………..1 Agree…………………………………………2 Disagree…………………………………….3 Strongly disagree……………………….4 |
|  |  |  |
| PS008 | Most people in this village do favors for each other (such as, watching your house when you are gone, taking care of other people’s children in an emergency, or lending people small things, or other actions like this). | Strongly agree (cohesion)…………..1 Agree…………………………………………2 Disagree…………………………………….3 Strongly disagree……………………….4 |
|  |  |  |
| PS009 | In general, how safe is your village? | Extremely safe……………………………1 Safe……………………………………………2  Not safe……………………………………..3 Extremely unsafe……………………….4 |
|  |  |  |
| PS010 | Do you feel as if you are really part of (a member of) this village? | YES................................................1  NO ................................................2 |

**Section: PTSD**

| PTSD001 | Now I have some questions about stressful experiences you may have had in the past.  In your life, have you ever had any experience that was so frightening, horrible, or upsetting that, in the past 30 days you avoided being reminded of this experience by staying away from certain places, people or activities? | YES................................................1  NO ................................................2 |
| --- | --- | --- |
|  |  |  |
| PTSD002 | In your life, have you ever had any experience that was so frightening, horrible, or upsetting that, in the past 30 days you lost interest in activities that were once important or enjoyable? | YES................................................1  NO ................................................2 |
|  |  |  |
| PTSD003 | In your life, have you ever had any experience that was so frightening, horrible, or upsetting that, in the past 30 days you began to feel more isolated or distant from other people? | YES................................................1  NO ................................................2 |
|  |  |  |
| PTSD004 | In your life, have you ever had any experience that was so frightening, horrible, or upsetting that, in the past 30 days you found it hard to have love or affection for other people? | YES................................................1  NO ................................................2 |
|  |  |  |
| PTSD005 | In your life, have you ever had any experience that was so frightening, horrible, or upsetting that, in the past 30 days you began to feel that there was no point in planning for the future? | YES................................................1  NO ................................................2 |
|  |  |  |
| PTSD006 | In your life, have you ever had any experience that was so frightening, horrible, or upsetting that, in the past 30 days you had more trouble than usual falling asleep or staying asleep? | YES................................................1  NO ................................................2 |
|  |  |  |
| PTSD007 | In your life, have you ever had any experience that was so frightening, horrible, or upsetting that, in the past 30 days you became become jumpy or got easily startled by ordinary noises or movements? | YES................................................1  NO ................................................2 |

**Section: Hearing Vision**

| HEV001 | Now I will ask you some questions about your hearing and vision.  Have you ever worn a hearing aid? | YES................................................1  NO ................................................2 |
| --- | --- | --- |
|  |  |  |
| HEV002 | Do you wear a hearing aid now? | YES................................................1  NO ................................................2 |
|  |  |  |
| HEV003 | How often do you usually wear a hearing aid these days? | Practically always use it……………..1 Occasionally or frequently use it……………………………………………….2 Never or almost never use it……..3 |
|  |  |  |
| HEV004 | If a person speaks to you in a normal voice, can you usually hear and understand him/her without seeing his/her face, whether or not you wear a hearing aid? | YES................................................1  NO ................................................2 |
|  |  |  |
| HEV005 | Now I have some questions about your eyesight  Do you have difficulty with your vision? | YES................................................1  NO ................................................2 |
|  |  |  |
| HEV006 | Do you usually wear glasses or corrective lenses? | Yes……………………………………………..1  No………………………………………………2  Legally blind……………………………….3 |
|  |  |  |
| HEV007 | How good is your eyesight for seeing things at a distance, like recognizing a friend from across the street (with glasses or corrective lenses if you wear them)? Would you say your eyesight for seeing things at a distance is excellent, very good, good, fair, or poor? | Excellent…………………………………….1  Very good…………………………………..2  Good………………………………………….3  Fair…………………………………………….4  Poor…………………………………………..5 |
|  |  |  |
| HEV008 | How good is your eyesight for seeing things up close, like reading ordinary newspaper print (with glasses or corrective lenses if you wear them)? Would you say your eyesight for seeing things up close is excellent, very good, good, fair, or poor? | Excellent…………………………………….1  Very good…………………………………..2  Good………………………………………….3  Fair…………………………………………….4  Poor…………………………………………..5 |
|  |  |  |
| HEV009 | Has a doctor, nurse, health care worker, ever told you that you have a cataract? | YES................................................1  NO ................................................2 |
|  |  |  |
| HEV010 | Have you ever had cataract surgery? | YES................................................1  NO ................................................2 |
|  |  |  |
| HEV011 | Have you had cataract surgery on both eyes or just one? | One eye only.................................1  Both eyes......................................2 |

**Section: Cardiometabolic**

| CM001 | The following questions ask about your health associated with a few chronic conditions.  Has a doctor, nurse, or other healthcare worker ever measured your blood pressure? | YES................................................1  NO ................................................2 |
| --- | --- | --- |
|  |  |  |
| CM002 | Have you ever been told by a doctor, nurse, or other healthcare worker that you have high blood pressure or hypertension? | YES................................................1  NO ................................................2 |
|  |  |  |
| CM003 | Have you been newly-diagnosed with high blood pressure in the last 12 months? | YES................................................1  NO ................................................2 |
|  |  |  |
| CM004 | Have you ever received treatment for high blood pressure prescribed by a doctor, nurse, or other healthcare worker? | YES................................................1  NO ................................................2 |
|  |  |  |
| CM005 | Are you currently on treatment for high blood pressure prescribed by a doctor, nurse, or other healthcare worker? | YES................................................1  NO ................................................2 |
|  |  |  |
| CM006 | Are you currently taking any herbal or traditional remedy for your high blood pressure? | YES................................................1  NO ................................................2 |
|  |  |  |
| CM007 | Has a doctor, nurse, or other healthcare worker ever measured your urine or blood for diabetes? | YES................................................1  NO ................................................2 |
|  |  |  |
| CM007_females | Have you ever been told by a doctor, nurse, or other healthcare worker that you have raised blood sugar or diabetes outside of pregnancy? | YES................................................1  NO ................................................2 |
|  |  |  |
| CM007_males | Have you ever been told by a doctor, nurse, or other healthcare worker that you have raised blood sugar or diabetes? | YES................................................1  NO ................................................2 |
|  |  |  |
| CM008 | Have you been newly-diagnosed with diabetes in the last 12 months? | YES................................................1  NO ................................................2 |
|  |  |  |
| CM009 | Have you ever received treatment for diabetes prescribed by a doctor, nurse or other healthcare worker? | YES................................................1  NO ................................................2 |
|  |  |  |
| CM010 | Are you currently receiving any treatment for diabetes prescribed by a doctor, nurse, or other healthcare worker? | YES................................................1  NO ................................................2 |
|  |  |  |
| CM011 | What are you doing to treat your diabetes? [IWER: CHECK ALL THAT APPLY.] | Special diet………………………………..1 Weight loss………………………………..2 Medication that you swallow……..3  Insulin injection…………………………4 Other………………………………………….5  None………………………………………….6 |
|  |  |  |
| CM012 | Are you currently taking any herbal or traditional remedy for your diabetes? | YES................................................1  NO ................................................2 |
|  |  |  |
| CM013 | Have you ever been tested for HIV? | YES................................................1  NO ................................................2 |
|  |  |  |
| CM093 | Do you know your status? | YES……………………………...............1  NO ……………………………...............2 |
|  |  |  |
| CM014 | Have you ever tested positive for HIV? | YES................................................1  NO ................................................2 |
|  |  |  |
| CM015 | Have you ever accessed an HIV treatment and care program? | YES................................................1  NO ................................................2 |
|  |  |  |
| CM016 | Have you ever received ART prescribed by a doctor, nurse or other healthcare worker? | YES................................................1  NO ................................................2 |
|  |  |  |
| CM017 | Are you currently receiving ART prescribed by a doctor, nurse or other healthcare worker? | YES................................................1  NO ................................................2 |
|  |  |  |
| CM018 | Are you currently taking any herbal or traditional remedy for HIV? | YES................................................1  NO ................................................2 |
|  |  |  |
| CM019 | Have you ever been counselled by a doctor, nurse, ART/HIV counselor, or other health worker on how you can avoid passing on HIV to others? | YES................................................1  NO ................................................2 |
|  |  |  |
| CM020 | Have you ever been told by a doctor, nurse, or other healthcare worker that you have TB? | YES................................................1  NO ................................................2 |
|  |  |  |
| CM020_newly | Have you been newly diagnosed with TB in the last 12 months? | YES................................................1  NO ................................................2 |
|  |  |  |
| CM021 | Have you ever received TB treatment prescribed by a doctor, nurse or other healthcare worker? | YES................................................1  NO ................................................2 |
|  |  |  |
| CM022 | Are you currently receiving TB treatment prescribed by a doctor, nurse or other healthcare worker? | YES................................................1  NO ................................................2 |
|  |  |  |
| CM023 | Are you currently taking any herbal or traditional remedy for TB? | YES................................................1  NO ................................................2 |
|  |  |  |
| CM024 | Have you ever been counselled by a doctor, nurse, ART/HIV counselor, or other health worker on how you can avoid passing on TB to others? | YES................................................1  NO ................................................2 |
|  |  |  |
| CM025 | Have you ever been told by a doctor, nurse, or other healthcare worker that you have had a stroke? | YES................................................1  NO ................................................2 |
|  |  |  |
| CM025_a | When was it first diagnosed? | _______________________Year |
|  |  |  |
| CM025_b | Have you ever been told by a doctor, nurse of other healthcare worker that you have had a ministroke, or transient ischemic attack? | YES................................................1  NO ................................................2 |
|  |  |  |
| CM025_c | Have you ever suddenly lost one half of your vision? | YES................................................1  NO ................................................2 |
|  |  |  |
| CM026 | Have you ever received medical treatment for this stroke from a doctor, nurse, or other healthcare worker? | YES................................................1  NO ................................................2 |
|  |  |  |
| CM027 | Are you currently on treatment to prevent a further stroke prescribed by a doctor, nurse or other healthcare worker? | YES................................................1  NO ................................................2 |
|  |  |  |
| CM028 | Are you currently taking any herbal or traditional remedy for a stroke? | YES................................................1  NO ................................................2 |
|  |  |  |
| CM029 | Have you ever been told by a doctor, nurse, or other healthcare worker that you have angina (chest pain due to heart disease)? | YES................................................1  NO ................................................2 |
|  |  |  |
| CM030 | Have you ever received treatment for angina (chest pain due to a heart disease) prescribed by a doctor, nurse or other healthcare worker? | YES................................................1  NO ................................................2 |
|  |  |  |
| CM031 | Are you currently taking any medications for angina (chest pain due to heart disease) prescribed by a doctor, nurse, or other healthcare worker? | YES................................................1  NO ................................................2 |
|  |  |  |
| CM032 | Are you currently taking any herbal or traditional remedy for your angina (chest pain due to heart disease)? | YES….............................................1  NO ….............................................2 |
|  |  |  |
| CM033 | During the last 12 months, have you experienced any pain or discomfort in your chest, or pain going to the left arm or neck when you walk uphill or hurry? | Yes……………………………………………..1  No………………………………………………2  Never walk uphill or hurry………….3 |
|  |  |  |
| CM034 | During the last 12 months, have you experienced any pain or discomfort in your chest, or pain going to the left arm or neck when you walk at an ordinary pace on level ground? | YES….............................................1  NO ….............................................2 |
|  |  |  |
| CM035 | What do you do if you get the pain or discomfort when you are walking? [IWER: READ CHOICES.] | Stop or slow down…………………….1 Carry on after taking a pain relieving medicine that dissolves in your mouth (e.g. A nitro spray or tablet)……………………………………….2 Carry on walking………………………..3 |
|  |  |  |
| CM036 | If you stand still, what happens to the pain or discomfort? [IWER: READ CHOICES.] |  |
|  |  |  |
| CM037 | Will you show me where you usually experience the pain or discomfort? [IWER: ENTER NUMBER RESPONDENT POINTS AT] |  |
|  |  |  |
| CM038 | Have you ever been told by a doctor, nurse, or other healthcare worker that you have had a heart attack? | YES……..........................................1  NO ……..........................................2 |
|  |  |  |
| CM039 | Did you receive medical treatment for your heart attack? | YES……..........................................1  NO ……..........................................2 |
|  |  |  |
| CM040 | Are you currently taking any herbal or traditional remedy for heart attack? | YES……..........................................1  NO ……..........................................2 |
|  |  |  |
| CM041 | Have you ever been told by a doctor, nurse, or other healthcare worker that you have had heart failure? | YES……….......................................1  NO ……….......................................2 |
|  |  |  |
| CM042 | Have you ever received medical treatment for your heart failure prescribed by a doctor, nurse or other healthcare worker? | YES…………....................................1  NO …………....................................2 |
|  |  |  |
| CM043 | Are you currently on treatment for heart failure prescribed by a doctor, nurse or other healthcare worker? | YES…………....................................1  NO …………....................................2 |
|  |  |  |
| CM044 | Are you currently taking any herbal or traditional remedy for heart failure? | YES…………....................................1  NO …………....................................2 |
|  |  |  |
| CM045 | Has a doctor, nurse, or other healthcare worker ever measured your cholesterol? | YES…………….................................1  NO …………….................................2 |
|  |  |  |
| CM046 | Have you ever been told by a doctor, nurse, or other healthcare worker that you have high cholesterol? | YES…………….................................1  NO …………….................................2 |
|  |  |  |
| CM047 | Have you ever been treated for high cholesterol by a doctor, nurse or other healthcare worker? | YES…………….................................1  NO …………….................................2 |
|  |  |  |
| CM048 | Are you currently using any of the following to treat your high cholesterol prescribed by a doctor, nurse, or other healthcare worker? [IWER: CHECK ALL THAT APPLY.] | Special diet…………………………………1 Weight loss………………………………..2 Medication…………………………………3 Other………………………………………….4 None………………………………………….5 |
|  |  |  |
| CM049 | Are you currently taking any herbal or traditional remedy for high cholesterol? | YES…………….................................1  NO …………….................................2 |
|  |  |  |
| CM050 | Do you usually cough? | YES………………..............................1  NO ………………..............................2 |
|  |  |  |
| CM051 | When you cough, do you usually bring up phlegm from your chest? | YES………………..............................1  NO ………………..............................2 |
|  |  |  |
| CM052 | Have you brought up phlegm every day for at least 3 months during the last year? | YES………………..............................1  NO ………………..............................2 |
|  |  |  |
| CM053 | For how many years have you brought up phlegm in this way? | YES………………..............................1  NO ………………..............................2 |
|  |  |  |
| CM054 | Have you ever worked in a job for more than 1 year where you were regularly exposed to smoke, dust, fumes or strong smells? | YES………………..............................1  NO ………………..............................2 |
|  |  |  |
| CM055 | Some people forget to take their medicine. How often does this happen to you? | Very often………………………………….1  Often………………………………………….2  Sometimes…………………………………3  Rarely…………………………………………4  Never…………………………………………5 |
|  |  |  |
| CM056 | Some people miss out a dose of their medicine or adjust it to suit their own needs. How often do you do this? | Very often………………………………….1  Often………………………………………….2  Sometimes…………………………………3  Rarely…………………………………………4  Never…………………………………………5 |
|  |  |  |
| CM057 | Some people stop taking their medication when they feel better. How often do you do this? | Very often………………………………….1  Often………………………………………….2  Sometimes…………………………………3  Rarely…………………………………………4  Never…………………………………………5 |
|  |  |  |
| CM058 | Some people stop taking their medication when they feel worse. How often do you do this? | Very often………………………………….1  Often………………………………………….2  Sometimes…………………………………3  Rarely…………………………………………4  Never…………………………………………5 |
|  |  |  |
| CM059 | Now I have some questions about various health behaviors. This includes things like smoking, drinking alcohol, eating fruits and vegetables, and physical activity. Let's start with tobacco.  Have you ever smoked any tobacco product such as cigarettes, cigars or pipes? | YES…………………...........................1  NO …………………...........................2 |
|  |  |  |
| CM060 | Has a doctor, nurse, or other healthcare worker ever advised you to stop smoking? | YES…………………...........................1  NO …………………...........................2 |
|  |  |  |
| CM061 | Do you currently smoke any tobacco products, such as cigarettes, cigars, or pipes? [IWER: FOR Q46, WHEN WE ASK IF THE RESPONDENT CURRENTLY SMOKES WE ARE ASKING IF THEY SMOKE IF THEY HAVE THE CHANCE.] | YES…………………...........................1  NO …………………...........................2 |
|  |  |  |
| CM062 | How often do you smoke tobacco products? | Daily…………………………………………..1 5-6 days per week………………………2 1-4 days per week………………………3 1-3 days per month……………………4 Less than once per month………….5 |
|  |  |  |
| CM063_intro | On days you smoke, how many of the following do you smoke? |  |
|  |  |  |
| CM063_cigar | Cigars, cheroots | ______________________Per day |
|  |  |  |
| CM063_handcig | Hand-rolled cigarettes… | ______________________Per day |
|  |  |  |
| CM063_mancig | Manufactured cigarettes | ______________________Per day |
|  |  |  |
| CM063_other | Other | ______________________Per day |
|  |  |  |
| CM063_pipe | Pipes full of tobacco | ______________________Per day |
|  |  |  |
| CM064 | How old were you when you first started smoking? | ________________________Years |
|  |  |  |
| CM065_age | At what age did you totally quit smoking or consuming tobacco? | Age: ________________________ |
|  |  |  |
| CM065_year |  | OR Year quit smoking: _________ |
|  |  |  |
| CM065_yearsago |  | OR _________________Years ago |
|  |  |  |
| CM066 | Have you ever used any smokeless tobacco such as snuff, chewing tobacco, snus, betel with tobacco? | YES……………………........................1  NO ……………………........................2 |
|  |  |  |
| CM067 | Do you currently use smokeless tobacco products? | YES……………………........................1  NO ……………………........................2 |
|  |  |  |
| CM068 | The next questions are about alcohol.  Have you ever consumed an alcoholic drink such as beer, wine, spirits, fermented cider, thothotho, or traditional beer? | YES……………………........................1  NO ……………………........................2 |
|  |  |  |
| CM069 | Do you currently (or in the last 30 days) consume any alcoholic drinks such as beer, wine, spirits, fermented cider, thothotho or traditional beer? | YES……………………........................1  NO ……………………........................2 |
|  |  |  |
| CM070 | How often do you have at least one alcoholic drink? | Daily…………………………………………..1 5-6 days per week………………………2 1-4 days per week………………………3 1-3 days per month……………………4 Less than once per month………….5 |
|  |  |  |
| CM071 | On days you drink, how many alcoholic drinks do you have? [IWER: SHOWCARD FOR STANDARD DRINKS] | Less than 1…………………………………1 1-2……………………………………………..2 3-4……………………………………………..3 5 or more…………………………………..4 |
|  |  |  |
| CM072 | Have you ever felt that you should cut down on your drinking? | YES……………………........................1  NO ……………………........................2 |
|  |  |  |
| CM073 | Have people annoyed you by criticizing your drinking? | YES……………………........................1  NO ……………………........................2 |
|  |  |  |
| CM074 | Have you ever felt bad or guilty about your drinking? | YES……………………….....................1  NO ……………………….....................2 |
|  |  |  |
| CM075 | Have you ever had an alcoholic drink first thing in the morning to steady your nerves or get rid of a hangover? [IWER: ALSO CALLED AN "EYE OPENER".] | YES……………………….....................1  NO ……………………….....................2 |
|  |  |  |
| CM076 | In the past year, did you ever take 6 or more drinks in a single morning, afternoon, or night? I understand that you may share drinks and that some drinks have different sizes, but please do your best to answer. For the purposes of this question, “one drink” should be considered equal to 1 shot or 1 tot of a strong alcoholic drink like spirits, or 1 full glass of a light alcoholic drink like beer. | YES…………………………..................1  NO …………………………..................2 |
|  |  |  |
| CM077 | What type of alcohol did/do you mainly drink? | Local brew………………………………….1 Spirits…………………………………………2 Beer……………………………………………3 Wine………………………………………….4 Other…………………………………………5 |
|  |  |  |
| CM078 | The next questions ask about what you eat and drink. In a typical week, on how many days do you eat fruit? [IWER: USE SHOWCARD] | ________________________Days |
|  |  |  |
| CM079 | How many servings of fruit do you eat on a typical day? (on any one day) | _____________________Servings |
|  |  |  |
| CM080 | In a typical week, on how many days do you eat vegetables? [IWER: USE SHOWCARD] | ________________________Days |
|  |  |  |
| CM081 | How many servings of vegetables do you eat on a typical day? (on any one day) | _____________________Servings |
|  |  |  |
| CM082 | In a typical week, how many meals per week do you buy from a vendor or take-away or restaurant? By meal, I mean breakfast, lunch or dinner. | _______________________Meals |
|  |  |  |
| CM083 | In a typical week, on how many days do you eat bread bought from a shop? [IWER: USE SHOWCARDS TO SHOW WHAT IS MEANT BY SHOP BOUGHT BREAD] | ________________________Days |
|  |  |  |
| CM084 | How many slices of bread bought from a shop do you eat on a typical day? [IWER: USE SHOWCARDS TO SHOW WHAT IS MEANT BY SLICES.] | _______________________Slices |
|  |  |  |
| CM085 | How many cans or bottles or cups of sugary drinks (e.g. Coke, Pepsi, Fanta, Sprite, etc.) do you drink in a typical week? [IWER: USE SHOWCARDS OF POPULAR DRINKS LOCALLY.] | _______________________Drinks |
|  |  |  |
| CM086 | How many cans or bottles or cups of fruit juice do you drink in a typical week? (e.g. Liquifruit, Tropicana, Oros, etc.) [IWER: USE SHOWCARDS OF DRINKS SERVING SIZES.] | _______________________Drinks |
|  |  |  |
| CM087 | Has a doctor, nurse, or other healthcare worker ever told you to change your diet (eg. To eat less sugar)? | YES…………………………..................1  NO …………………………..................2 |
|  |  |  |
| CM088 | Has a doctor, nurse, or other healthcare worker ever advised you to lose weight? | YES…………………………..................1  NO …………………………..................2 |
|  |  |  |
| CM089 | Has a doctor, nurse, or other healthcare worker ever told you that you have kidney disease? | YES…………………………..................1  NO …………………………..................2 |
|  |  |  |
| CM090 | Do you know what type of kidney disease? | YES…………………………..................1  NO …………………………..................2 |
|  |  |  |
| CM091 | What type of kidney disease do you have? |  |
|  |  |  |
| CM092 | Has a doctor, nurse, or other healthcare worker ever told you that your kidneys have low function? | YES……………………………...............1  NO ……………………………...............2 |
|  |  |  |
| **Created Variables:** | **Cardiometabolic Section** |  |
| *Variable* | *Label* | *Source* |
|  |  |  |
| c_cm_diabetes_srdiag | Diabetes broad definition (includes self-report of ever diagnosed) | Diabetes broad definition (includes self-report of ever diagnosed): Self-reported diabetes diagnosis (cm010);  OR glucose ≥ 7 mmol/l (126 mg/dL) in fasting group (defined as > 8 hours), glucose ≥11.1 mmol/l (200 mg/dL) in nonfasting (“random or casual”) samples. Individuals with missing fasting information were considered to be not fasting. |
|  |  |  |
| c_cm_diabetes_srtrt | Diabetes narrow definition (includes self-report of current treatment) | Diabetes narrow definition (includes self-report of current treatment): Self-reported diabetes treatment (cm007_males, cm007_females);  OR glucose ≥ 7 mmol/l (126 mg/dL) in fasting group (defined as > 8 hours), glucose ≥11.1 mmol/l (200 mg/dL) in nonfasting (“random or casual”) samples. Individuals with missing fasting information were considered to be not fasting. |
|  |  |  |
| c_cm_dyslipidemia_srdiag | Dyslipidemia broad definition (includes self-report of ever diagnosed) | Dyslipidemia broad definition (includes self-report of ever diagnosed): Elevated total cholesterol (≥6.21 mmol/L), if c_bs_chol not flagged; OR  Low HDL (1.19 mmol/L), if c_bs_hdl not flagged; OR  Elevated LDL (>4.1 mmol/L), if c_bs_ldl not flagged; OR  Elevated triglycerides (>2.25 mmol/L), if c_bs_trig not flagged; OR  Reports ever diagnosed with high cholesterol |
|  |  |  |
| c_cm_dyslipidemia_srtrt | Dyslipidemia narrow definition (includes self-report of current treatment) | Dyslipidemia narrow definition (includes self-report of current treatment): Elevated total cholesterol (≥6.21 mmol/L), if c_bs_chol not flagged; OR Low HDL (1.19 mmol/L), if c_bs_hdl not flagged; OR Elevated LDL (>4.1 mmol/L), if c_bs_ldl not flagged; OR Elevated triglycerides (>2.25 mmol/L), if c_bs_trig not flagged; OR Reports high cholesterol treatment at time of interview |
|  |  |  |
| c_cm_hypertension_srdiag | Hypertension broad definition (includes self-report of ever diagnosed) | Hypertension broad definition (includes self-report of ever diagnosed): Systolic BP ≥ 140 mmHg, or diastolic BP ≥ 90 mmHg, or reports using anti-hypertensive medication at time of interview |
| c_cm_hypertension_srtrt | Hypertension narrow definition (includes self-report of current treatment) | Hypertension narrow definition (includes self-report of current treatment): Systolic BP ≥ 140 mmHg, or diastolic BP ≥ 90 mmHg, or reports using anti-hypertensive medication at time of interview |

**Section: Physical Activity**

| PA001 | Has a doctor, nurse, or other healthcare worker ever advised you to start or do more exercise? | YES................................................1  NO ................................................2 |
| --- | --- | --- |
|  |  |  |
| PA002 | The following questions are about the time you spend doing different types of physical activities. This includes activities you do at home, at work, travelling from place to place and during your spare time. Work can be paid or unpaid. You are requested to answer the questions even if you don’t consider yourself an active person. [IWER: EVEN IF RESPONDENT IS NOT CURRENTLY WORKING FOR PAY, THEY SHOULD ANSWER THE FOLLOWING SERIES REGARDING THEIR USUAL UNPAID DAILY ACTIVITIES AT HOME.] How many days do you work per week? | ________________________Days |
|  |  |  |
| PA003 | Do you work over the weekend? | YES................................................1  NO ................................................2 |
|  |  |  |
| PA004 | Please consider your activity during a usual week. Think first about the time you spend doing work. Think of work as the things that you have to do such as paid or unpaid work, study/training, household chores, harvesting food/crops, fishing or hunting for food, seeking employment. In the following questions 'vigorous-intensity activities' are activities that require hard physical effort and cause large increases in breathing or heart rate, 'moderate-intensity activities' are activities that require moderate physical effort and cause small increases in breathing or heart rate. Does your work involve mostly sitting or standing still, or walking for very short periods (less than 10 minutes)? | YES................................................1  NO ................................................2 |
|  |  |  |
| PA005 | Does your work involve vigorous activities (heavy lifting, digging, manual labour or construction) for at least 10 minutes at a time? | YES................................................1  NO ................................................2 |
|  |  |  |
| PA006 | In a usual week, how many days are spent doing vigorous activities as part of your work? | ________________________Days |
|  |  |  |
| PA007_hours | On a usual day of vigorous work, how many hours are spent doing these activities? | _______________________Hours |
|  |  |  |
| PA007_minutes |  | _____________________Minutes |
|  |  |  |
| PA008 | Does your work involve moderate-intensity activities (brisk walking or carrying light loads) for at least 10 minutes at a time? | YES................................................1  NO ................................................2 |
|  |  |  |
| PA009 | In a usual week, how many days are spent doing moderate-intensity activities at work? | ________________________Days |
|  |  |  |
| PA010_hours | On a usual work day, how many hours are spent doing moderate-intensity activities? | _______________________Hours |
|  |  |  |
| PA010_minutes |  | _____________________Minutes |
|  |  |  |
| PA011 | How long is your usual work day? [IWER: PLEASE INDICATE HOW MANY HOURS YOU WORK PER DAY ON AVERAGE.] | _______________________Hours |
|  |  |  |
| PA012 | Please consider your activity during a usual week. These questions exclude the physical activities at work that you have already mentioned. These questions are about the usual way you travel to and from places. For example to work, for shopping, to market, to place of worship.  Do you walk or use a bicycle (for at least 10 minutes at a time) to get to and from places? | YES................................................1  NO ................................................2 |
|  |  |  |
| PA013 | In a usual week, how many days do you walk or cycle for at least 10 minutes to get to and from places? | ________________________Days |
|  |  |  |
| PA014_hours | On a usual day, how many hours do you spend walking or cycling for travel? | _______________________Hours |
|  |  |  |
| PA014_minutes |  | _____________________Minutes |
|  |  |  |
| PA015 | Please consider your activity during a usual week. The next questions, exclude the work and transport activities that you have already mentioned, they are about sports, fitness and recreational activities (leisure).  In your spare time, do you engage in any vigorous or moderate-intensity physical activities lasting more than 10 minutes at a time? | YES................................................1  NO ................................................2 |
|  |  |  |
| PA016 | In your spare time do you do any vigorous activities like running, strenuous sport or exercise for at least 10 minutes at a time? | YES................................................1  NO ................................................2 |
|  |  |  |
| PA017 | In a usual week, how many days do you engage in vigorous activities as part of your leisure time? | ________________________Days |
|  |  |  |
| PA018_hours | In a normal day, how many leisure hours are spent doing vigorous activities? | _______________________Hours |
|  |  |  |
| PA018_minutes |  | _____________________Minutes |
|  |  |  |
| PA019 | In your spare time, do you engage in any moderately intense physical activities like walking or swimming for at least 10 minutes at a time? | YES................................................1  NO ................................................2 |
|  |  |  |
| PA020 | In a normal week, how many days are spent engaging in moderately intense physical activity as part of your leisure time? | ________________________Days |
|  |  |  |
| PA021_hours | How many leisure hours are spent doing moderate-intensity activities in a normal day? | _______________________Hours |
|  |  |  |
| PA021_minutes |  | _____________________Minutes |
|  |  |  |
| PA022_hours | Please consider your activity during a usual week. The rest of the questions will provide extra information on sedentary behaviour.  On a usual week day, how many hours did you spend sitting or reclining (excluding sleep)? This may include time sitting on a chair or bench, visiting friends, reading, sitting in church, sitting down to watch television. | _______________________Hours |
|  |  |  |
| PA022_minutes |  | _____________________Minutes |
|  |  |  |
| PA023_hours | On a usual weekend day, how many hours did you spend sitting or reclining (excluding sleep)? This may include time sitting on a chair or bench, visiting friends, reading, sitting in church, sitting down to watch television. | _______________________Hours |
|  |  |  |
| PA023_minutes |  | _____________________Minutes |
|  |  |  |
| PA024_hours | How many hours do you spend sitting watching TV per day, during the week? | _______________________Hours |
|  |  |  |
| PA024_minutes |  | _____________________Minutes |
|  |  |  |
| PA025_hours | How many hours per day, do you spend watching TV during the weekend? | _______________________Hours |
|  |  |  |
| PA025_minutes | How many minutes per day, do you spend watching TV during the weekend? | _____________________Minutes |

**Section: Physical Functioning**

| PF001 | We need to understand difficulties people may have with various activities because of a health or physical problem. Please tell me whether you have difficulty performing any of the following tasks on a regular basis. Exclude any difficulties that you expect to last less than three months.  Because of a health or memory problem do you have any difficulty with walking across a room? | Yes……………………………………………..1  No………………………………………………2  Can’t do……………………………………..3  Don’t want to…………………………….4 |
| --- | --- | --- |
|  |  |  |
| PF002 | Do you ever use equipment or devices (e.g., cane) when crossing a room? | YES................................................1  NO ................................................2 |
|  |  |  |
| PF003 | What equipment is that? [IWER: CHECK ALL THAT APPLY.] | Railing………………………………………..1 Walker……………………………………….2 Cane…………………………………………..3 Crutches…………………………………….4 Orthopedic shoes……………………….5 Leg/back brace…………………………..6 Prosthesis…………………………………..7 Oxygen/respirator……………………..8 Furniture/walls…………………………..9 Wheelchair/cart……………………….10 Other……………………………………….11 |
|  |  |  |
| PF004 | Does anyone ever help you get across a room? | YES................................................1  NO ................................................2 |
|  |  |  |
| PF005 | Because of health and memory problems, do you have any difficulty with dressing? Dressing includes taking clothes out, putting them on, buttoning up, and fastening a belt. | Yes……………………………………………..1  No………………………………………………2  Can’t do……………………………………..3  Don’t want to…………………………….4 |
|  |  |  |
| PF006 | Do you ever use equipment or devices (e.g., cane) when dressing? | YES................................................1  NO ................................................2 |
|  |  |  |
| PF007 | What equipment is that? [IWER: CHECK ALL THAT APPLY.] | Railing………………………………………..1 Walker……………………………………….2 Cane…………………………………………..3 Crutches…………………………………….4 Orthopedic shoes……………………….5 Leg/back brace…………………………..6 Prosthesis…………………………………..7 Oxygen/respirator……………………..8 Furniture/walls…………………………..9 Wheelchair/cart……………………….10 Other……………………………………….11 |
|  |  |  |
| PF008 | Does anyone ever help you with dressing? | YES................................................1  NO ................................................2 |
|  |  |  |
| PF009 | Because of health and memory problems, do you have any difficulty with bathing or showering? | Yes……………………………………………..1  No………………………………………………2  Can’t do……………………………………..3  Don’t want to…………………………….4 |
|  |  |  |
| PF010 | Do you ever use equipment or devices (e.g., cane) when bathing or showering? | YES................................................1  NO ................................................2 |
|  |  |  |
| PF011 | What equipment is that? [IWER: CHECK ALL THAT APPLY.] | Railing………………………………………..1 Walker……………………………………….2 Cane…………………………………………..3 Crutches…………………………………….4 Orthopedic shoes……………………….5 Leg/back brace…………………………..6 Prosthesis…………………………………..7 Oxygen/respirator……………………..8 Furniture/walls…………………………..9 Wheelchair/cart……………………….10 Other……………………………………….11 |
|  |  |  |
| PF012 | Does anyone ever help you with bathing or showering? | YES................................................1  NO ................................................2 |
|  |  |  |
| PF013 | Because of health and memory problems, do you have any difficulty with eating, such as cutting up your food? (Definition: By eating, we mean eating food by oneself when it is ready.) | Yes……………………………………………..1  No………………………………………………2  Can’t do……………………………………..3  Don’t want to…………………………….4 |
|  |  |  |
| PF014 | Does anyone ever help you with eating? | YES................................................1  NO ................................................2 |
|  |  |  |
| PF015 | Do you have any difficulty with getting into or out of the place where you sleep? | Yes……………………………………………..1  No………………………………………………2  Can’t do……………………………………..3  Don’t want to…………………………….4 |
|  |  |  |
| PF016 | Do you ever use equipment or devices (e.g., cane) when getting into or out of the place where you sleep? | YES................................................1  NO ................................................2 |
|  |  |  |
| PF017 | What equipment is that? [IWER: CHECK ALL THAT APPLY.] | Railing………………………………………..1 Walker……………………………………….2 Cane…………………………………………..3 Crutches…………………………………….4 Orthopedic shoes……………………….5 Leg/back brace…………………………..6 Prosthesis…………………………………..7 Oxygen/respirator……………………..8 Furniture/walls…………………………..9 Wheelchair/cart……………………….10 Other……………………………………….11 |
|  |  |  |
| PF018 | Does anyone ever help you with getting into or out of the place where you sleep? | YES................................................1  NO ................................................2 |
|  |  |  |
| PF019 | Because of health and memory problems, do you have any difficulties with using the toilet, including getting up and down? | Yes……………………………………………..1  No………………………………………………2  Can’t do……………………………………..3  Don’t want to…………………………….4 |
|  |  |  |
| PF020 | Do you ever use equipment or devices (e.g., cane) when using the toilet? | YES................................................1  NO ................................................2 |
|  |  |  |
| PF021 | What equipment is that? [IWER: CHECK ALL THAT APPLY.] | Railing………………………………………..1 Walker……………………………………….2 Cane…………………………………………..3 Crutches…………………………………….4 Orthopedic shoes……………………….5 Leg/back brace…………………………..6 Prosthesis…………………………………..7 Oxygen/respirator……………………..8 Furniture/walls…………………………..9 Wheelchair/cart……………………….10 Other……………………………………….11 |
|  |  |  |
| PF022 | Does anyone ever help you with using the toilet? | YES................................................1  NO ................................................2 |
|  |  |  |
| PF023 | Do you use any of the following? [IWER: REGULAR USE. CHECK ALL THAT APPLY.] | Walking stick………………………………1 Travel device………………………………2 Manual wheelchair…………………….3 Electric Wheelchair…………………….4 Catheter, urine collection bag…….5 Toilet Series……………………………….6 None of the above……………………..7 |
|  |  |  |
| PF024 | Who most often helps you with dressing, bathing, eating, getting out of bed, using the toilet, controlling urination and defecation, doing chores, preparing hot meals, shopping, managing money, making phone calls, taking medications? [IWER: CHOOSE UP TO 3 PEOPLE.] | Spouse……………………………………….1 Mother………………………………………2 Father………………………………………..3 Mother-in-law……………………………4 Father-in-law……………………………..5 Children…………………………………..…6 Sibling………………………………………..7 Sibling of spouse………………………..8 Brother-in-law, sister-in-law………9　 Spouse of child…………………………10 Grandchild……………………………….11 Other relative…………………………..12 Paid helper(such as nanny)………13 Volunteer or Employee of facility………………………………………14 Other……………………………………….15 No one helped………………………….16 |
|  |  |  |
| **Created Variables:** | **Physical Functioning Section** |  |
| *Variable* | *Label* | *Source* |
|  |  |  |
| c_pf_diff_bath | ADL difficulty bathing | Difficulty bathing (derived from pf009) |
|  |  |  |
| c_pf_diff_bed | ADL difficulty getting in/out of bed | Difficulty getting in / out of bed (derived from pf015) |
|  |  |  |
| c_pf_diff_dress | ADL difficulty dressing | Difficulty dressing (derived from pf005) |
|  |  |  |
| c_pf_diff_eat | ADL difficulty eating | Difficulty eating (derived from pf013) |
|  |  |  |
| c_pf_diff_toilet | ADL difficulty using toilet | Difficulty using the toilet (derived from pf019) |
|  |  |  |
| c_pf_diff_walk | ADL difficulty walking across room | Difficulty walking (derived from pf001) |

**Section: CESD**

| CD001 | Now think about the past week and the feelings you have experienced. Please tell me if each of the following was true for you much of the time this past week. Would you say yes or no?  Much of the time in the past week, you felt depressed | YES................................................1  NO ................................................2 |
| --- | --- | --- |
|  |  |  |
| CD002 | Much of the time in the past week, you felt that everything you did was an effort.  **Note: This question was mistranslated and must be reverse-coded in the CESD scale. | YES................................................1  NO ................................................2 |
|  |  |  |
| CD003 | Much of the time in the past week, your sleep was restless. | YES................................................1  NO ................................................2 |
|  |  |  |
| CD004 | Much of the time in the past week, you were happy. | YES................................................1  NO ................................................2 |
|  |  |  |
| CD005 | Much of the time in the past week, you felt lonely. | YES................................................1  NO ................................................2 |
|  |  |  |
| CD006 | Much of the time in the past week, you did not enjoy life. | YES................................................1  NO ................................................2 |
|  |  |  |
| CD007 | Much of the time in the past week, you felt sad. | YES................................................1  NO ................................................2 |
|  |  |  |
| CD008 | Much of the time in the past week, you could not get "going". | YES................................................1  NO ................................................2 |
|  |  |  |
| **Created Variables:** | **Physical Functioning Section** |  |
| *Variable* | *Label* | *Source* |
|  |  |  |
| c_cd_cesd | Depression score (based on CESD-8 scale) | Summation of responses to cd001 through cd008. One point for each positive response, with reverse coding for cd002 and cd004. |

**Section: Pain**

| PN001 | Now I have some questions about pain. Throughout our lives, most of us have had pain from time to time (such as minor headaches, sprains and toothaches). Have you had pain – other than these everyday kinds of pain – today? | YES................................................1  NO ................................................2 |
| --- | --- | --- |
|  |  |  |
| PN002 | Please tell me where you feel pain. [IWER: MARK ALL AREAS RESPONDENT POINTED AT OR MENTIONS.] | Head………………………………………….1 Jaw…………………………………………….2 Neck…………………………………………..3 Shoulder(s)…………………………………4 Upper back…………………………………5  Lower back…………………………………6 Chest………………………………………….7 Abdomen……………………………………8 Hip(s)…………………………………………9 Leg(s)……………………………………….10 Knee(s)…………………………………….11 Ankle(s)……………………………………12 Foot/feet………………………………….13 Toe(s)……………………………………….14 Arm(s)………………………………………15 Elbow(s)……………………………………16 Wrist(s)…………………………………….17 Hand(s)…………………………………….18 Finger(s)…………………………………..19 Other, please specify……………….20 |
|  |  |  |
| PN002_most | Please tell me which of these areas hurts the most. | Head………………………………………….1 Jaw…………………………………………….2 Neck…………………………………………..3 Shoulder(s)…………………………………4 Upper back…………………………………5  Lower back…………………………………6 Chest………………………………………….7 Abdomen……………………………………8 Hip(s)…………………………………………9 Leg(s)……………………………………….10 Knee(s)…………………………………….11 Ankle(s)……………………………………12 Foot/feet………………………………….13 Toe(s)……………………………………….14 Arm(s)………………………………………15 Elbow(s)……………………………………16 Wrist(s)…………………………………….17 Hand(s)…………………………………….18 Finger(s)…………………………………..19  Other, please specify……………….20 |
|  |  |  |
| PN003 | On a scale of 0 to 10, where 0 is “No Pain” and 10 is “Pain as bad as you can imagine”, please rate your pain at its worst in the last 24 hours. | ____________________________ |
|  |  |  |
| PN004 | On a scale of 0 to 10, where 0 is “No Pain” and 10 is “Pain as bad as you can imagine”, please rate your pain at its least in the last 24 hours. | ____________________________ |
|  |  |  |
| PN005 | On a scale of 0 to 10, where 0 is “No Pain” and 10 is “Pain as bad as you can imagine”, please rate your pain on average. | ____________________________ |
|  |  |  |
| PN006 | On a scale of 0 to 10, where 0 is “No Pain” and 10 is “Pain as bad as you can imagine”, please rate how much pain you have right now. | ____________________________ |
|  |  |  |
| PN007 | Are you currently receiving any treatment or currently taking any medications for your pain? | YES................................................1  NO ................................................2 |
|  |  |  |
| PN008 | Are you currently taking any herbal or traditional remedy for your pain? | YES................................................1  NO ................................................2 |
|  |  |  |
| PN009 | On a scale of 0 to 10, where 0 is “Does not interfere” and 10 is “Completely interferes”, select the one number that describes how, during the past 24 hours, pain has interfered with your general activity. | ____________________________ |
|  |  |  |
| PN010 | On a scale of 0 to 10, where 0 is “Does not interfere” and 10 is “Completely interferes”, select the one number that describes how, during the past 24 hours, pain has interfered with your mood. | ____________________________ |
|  |  |  |
| PN011 | On a scale of 0 to 10, where 0 is “Does not interfere” and 10 is “Completely interferes”, select the one number that describes how, during the past 24 hours, pain has interfered with your walking ability. | ____________________________ |
|  |  |  |
| PN012 | On a scale of 0 to 10, where 0 is “Does not interfere” and 10 is “Completely interferes”, select the one number that describes how, during the past 24 hours, pain has interfered with your normal work (includes both work outside the home and housework). | ____________________________ |
|  |  |  |
| PN013 | On a scale of 0 to 10, where 0 is “Does not interfere” and 10 is “Completely interferes”, select the one number that describes how, during the past 24 hours, pain has interfered with your relations with other people. | ____________________________ |
|  |  |  |
| PN014 | On a scale of 0 to 10, where 0 is “Does not interfere” and 10 is “Completely interferes”, select the one number that describes how, during the past 24 hours, pain has interfered with your sleep. | ____________________________ |
|  |  |  |
| PN015 | On a scale of 0 to 10, where 0 is “Does not interfere” and 10 is “Completely interferes”, select the one number that describes how, during the past 24 hours, pain has interfered with your enjoyment of life. | ____________________________ |

**Section: HIV**

| HV001 | [IWER: MAKE SURE OF COMPLETE PRIVACY FOR THE FOLLOWING QUESTIONS.] Now I am going to ask you some very sensitive questions. How old were you the very first time you had sex? | Age:_____.....................................1  Never had sex…………………………..2 |
| --- | --- | --- |
|  |  |  |
| HV002 | How old were you the very first time you had sex? | ________________________Years |
|  |  |  |
| HV003 | How many different sexual partners have you had in your lifetime? | _____________________Partners |
|  |  |  |
| HV004 | Sometimes people have more than one relationship at the same time. How many relationships are you in at the moment? | _________________Relationships |
|  |  |  |
| HV005 | During the last 24 months (that is since ^FLHV005), how many sexual partners have you had? | _____________________Partners |
|  |  |  |
| HV006_intro | For the next section on partners, follow the definitions below:  1) Regular partner: is someone who is special to you, like a husband / wife / boyfriend / girlfriend, or lover.  2) Casual partner: is a person you know but who is not your main sex partner, whether you had sex only once or many times.  3) Anonymous partner: is someone whose name you did not know the day before you had sex. A sex partner who is a prostitute could be either casual or anonymous. |  |
|  |  |  |
| HV006 | Now, we are going to discuss the most recent person you had sexual intercourse with. How would you categorize this partner? | Regular………………………………………1 Casual………………………………………..2 Anonymous………………………………..3 |
|  |  |  |
| HV007 | What is/was your relationship with this partner? | Spouse with lobola done……………1  Living together without lobola……2 Girlfriend/Boyfriend…………………..3 |
|  |  |  |
| HV008_day | When did you get married or start living together? (Year, month and day) | _________________________Day |
|  |  |  |
| HV008_month |  | ______________________Month |
|  |  |  |
| HV008_year |  | ________________________Year |
|  |  |  |
| HV009 | Is/was this an ongoing relationship? | YES................................................1  NO ................................................2 |
|  |  |  |
| HV010_day | When did this partnership finish? (Year, month and day) | _________________________Day |
|  |  |  |
| HV010_month |  | ______________________Month |
|  |  |  |
| HV010_year | When did this partnership finish? | ________________________Year |
|  |  |  |
| HV012 | Is/was your partner younger or older than you? | Older………………………………………….1  Younger……………………………………..2  About the same age……………………3 |
|  |  |  |
| HV013 | Approximately how many years younger or older than you is/was your partner? | Same age……………………………………1 1 to 5………………………………………….2  6 to 10……………………………………….3 11 to 15……………………………………..4 16 to 20……………………………………..5 More than 20…………………….……...6 |
|  |  |  |
| HV014_time | How long ago did you first have sexual intercourse with this person? | Days:________________________  Weeks:______________________  Months:_____________________  Years:_______________________ |
|  |  |  |
| HV015 | Has/did your partner ever had sex with someone else during your relationship with him/her? | YES................................................1  NO ................................................2 |
|  |  |  |
| HV016_time | When (how long ago) was the last time you had sex with this partner? | Days:________________________  Weeks:______________________  Months:_____________________  Years:_______________________ |
|  |  |  |
| HV017 | Do/did you use a condom with this partner? | Always……………………………………….1  Most of the time………………………..2  Sometimes…………………………………3  Never…………………………………………4 |
|  |  |  |
| HV018 | Did you use a condom the last time you had sex with this partner? | YES................................................1  NO ................................................2 |
|  |  |  |
| HV019 | Have you had vaginal or anal sex without a condom when you were drunk or buzzed on alcohol or high on any other drug? | Yes................................................1  No ................................................2  I do not remember…………………..3 |
|  |  |  |
| HV020 | Have you had sex in exchange for money, drugs, goods or services? | YES................................................1  NO ................................................2 |
|  |  |  |
| HV021 | Did you use a condom? | Yes................................................1  No ................................................2  I do not remember…………………..3 |
|  |  |  |
| HV022 | Have you ever had sex without a condom with someone you know is HIV positive? | YES................................................1  NO ................................................2 |
|  |  |  |
| HV023 | When was the last time you had an HIV test? | In the last 6 months……………………1  6 months to 1 year ago………………2  More than a year ago…………………3  Never tested………………………………4 |
|  |  |  |
| HV024 | Where did you test the last time? | At home……………………………………1 Counseling center……………………..2 Public clinic……………………………….3 Mobile testing unit…………………….4  Hospital……………………………………..5 Private doctor/clinic………………….6 Pharmacy/Chemist……………………7 |
|  |  |  |
| HV025 | Have you ever informed anyone about your HIV status? | YES................................................1  NO ................................................2 |
|  |  |  |
| HV026 | To whom did you inform about your HIV status? [IWER: CHECK ALL THAT APPLY.] | Spouse/Current partner…………….1 Girlfriend/boyfriend…………………..2 Parent………………………………………..3 Brother/Sister…………………………….4 Someone else in the family………..5 Friend………………………………………..6 Religious leader………………………….7 Other………………………………………….8 |
|  |  |  |
| HV027 | Have you ever heard about anti-retroviral treatment (ART)? | YES................................................1  NO ................................................2 |
|  |  |  |
| HV028 | Do you think ART can help people to improve their health? | YES................................................1  NO ................................................2 |
|  |  |  |
| HV029 | Do you know where one could get ART? | YES................................................1  NO ................................................2 |
|  |  |  |
| HV030 | Where could one get ART? | At home……………………………………1 Counseling center……………………..2 Public clinic……………………………….3 Mobile testing unit…………………….4  Hospital……………………………………..5 Private doctor/clinic………………….6 Pharmacy/Chemist……………………7  Other, specify……………………………8 |
|  |  |  |
| HV031 | Has your fore skin been removed (i.e circumcision)? | YES................................................1  NO ................................................2 |
|  |  |  |
| HV032 | At what age were you circumcised? | ________________________Years |
|  |  |  |
| HV033 | When were you circumcised? | Birth…………………………………………..1 Infant (less 1 year old)………………..2 1 to 13……………………………………….3 14 to 18……………………………………..4 More than 18……………………………..5 |
|  |  |  |
| HV034 | Where were you circumcised? | Initiation…………………………………….1  Hospital……………………………………..2  Other………………………………………….3 |
|  |  |  |
| HV035 | Are you currently at risk of contracting HIV? | YES….............................................1  NO ….............................................2 |
|  |  |  |
| HV036 | Have you ever been at risk of contracting HIV in the past? | YES….............................................1  NO ….............................................2 |
|  |  |  |
| HV037 | HIV can be transmitted when an HIV-infected person has sex with an HIV-uninfected person. However, not every sex act between an HIV-infected and an HIV-uninfected person leads to an HIV infection. In the following questions, you will be asked to estimate the probability that a sex act leads to an HIV transmission. As an example of how we will ask you to answer this question, please consider the following example: “The probability of winning the lottery is 1 in ____” (the true probability of winning the lottery is somewhere around 1 in 10 million).  What do you think the probability is that a man would become infected with HIV from only one act of unprotected vaginal intercourse with an already infected woman? Probability a man would become infected = 1 in ... | ____________________________ |
|  |  |  |
| HV038 | What do you think the probability is that a woman would become infected with HIV from only one act of unprotected vaginal intercourse with an already infected man? Probability a woman would become infected = 1 in ... | ____________________________ |
|  |  |  |
| HV039 | Out of every 100 adults 15 years or older, how many do you think have been infected with HIV? | ____________________________ |
|  |  |  |
| HV040 | Out of every 100 adults 50 years or older, how many do you think have been infected with HIV? | ____________________________ |

**Section: Sleep**

| SP001_hour | Now I will ask you some questions about your sleep.  Over the past 4 weeks, what time did you usually turn the lights off to go to sleep? Please tell me the hour and minutes of the day, for example 20:30. | ________________________Hour |
| --- | --- | --- |
|  |  |  |
| SP001_minute |  | _____________________Minutes |
|  |  |  |
| SP002_hour | Over the past 4 weeks, what time did you usually get out of bed? | ________________________Hour |
|  |  |  |
| SP002_minute |  | _____________________Minutes |
|  |  |  |
| SP003 | Over the past 4 weeks, how many hours do you think you actually slept each day? This may be different than the number of hours you spent in bed. | ________________________Hour |
|  |  |  |
| SP004 | How often during the past 4 weeks did you get enough sleep to feel rested upon waking up? Would you say never, rarely, sometimes, often or very often? | Never…………………………………………1  Rarely…………………………………………2  Sometimes…………………………………3  Often………………………………………….4  Very often………………………………….5 |
|  |  |  |
| SP005 | During the past 4 weeks, how often did you wake up in the middle of the night or early morning? (Would you say never, less than once a week, once or twice a week, or three or more times a week?) | Never…………………………………………1  Less than once a week……………….2  Once or twice a week…………………3  Three or more times a week………4 |
|  |  |  |
| SP006 | During the past month, have you snored, or ever been told that you were snoring? | YES................................................1  NO ................................................2 |
|  |  |  |
| SP007 | During the past month, have you snored loudly, or ever been told that you were snoring loudly? | YES................................................1  NO ................................................2 |
|  |  |  |
| SP008 | During the last month, have you had, or ever been told that you were snorting or gasping? | YES................................................1  NO ................................................2 |
|  |  |  |
| SP009 | During the last month, have you had, or ever been told that your breathing stops or you struggle for breath? | YES................................................1  NO ................................................2 |
|  |  |  |
| SP010 | Over the past 4 weeks, how would you rate your sleep quality overall? Would you say it is very good, fairly good, fairly bad, or very bad? | Very good…………………………………..1  Fairly good…………………………………2  Fairly bad……………………………………3  Very bad…………………………………….4 |
|  |  |  |
| SP011 | During the past 4 weeks, how often could you not get to sleep within 30 minutes? Would you say never, less than once a week, once or twice a week, or three or more times a week? | Never…………………………………………1  Less than once a week……………….2  Once or twice a week…………………3  Three or more times a week………4 |

**Section: Tracking**

| TG001 | We would like to follow-up with this household in two years to see what, if anything, has changed. For this reason, I will ask for the contact information of people that may be able to tell us where you and your household are in 2 years, in case you move. This information will be kept separate from your responses in a separate, secure file where only the primary researcher can assess. Remember you are free to skip any questions that you are uncomfortable providing answers to. What other names are you commonly known by? | ____________________________ |
| --- | --- | --- |
|  |  |  |
| TG002 | Do you have access to a mobile phone or land line telephone? | YES................................................1  NO ................................................2 |
|  |  |  |
| TG003 | Could you please share with me your primary mobile phone number or land line for contacting you? | ____________________________ |
|  |  |  |
| TG004 | Could you also please share with me a second mobile phone number or land line number for contacting you? | ____________________________ |
|  |  |  |
| TG005 | Does your family/household have any plans to move in the next two years? | YES................................................1  NO ................................................2 |
|  |  |  |
| TG006 | Where do you plan to move to? | ____________________________ |
|  |  |  |
| TG007 | What is the name and mobile phone number of the head of this household? | Name:_______________________ |
|  |  |  |
| TG008 |  | Phone number:_______________ |
|  |  |  |
| TG009 | What is the name and mobile phone number of someone else within your household? | Name:_______________________ |
|  |  |  |
| TG010 |  | Phone number:_______________ |
|  |  |  |
| TG011 | What is this person's relationship to you? | (M) Mother………………………………..1 (F) Father……………………………………2 (B) Brother…………………………………3 (Z) Sister…………………………………….4 (S) Son………………………………………..5 (D) Daughter………………………………6  (T) HH head………………………………..7 (H) Husband……………………………….8 (W) Wife…………………………………….9 (R) Related indirectly………………..10 (U) Unrelated……………………………11 |
|  |  |  |
| TG012 | What is the name, location (address and description), and mobile phone number of someone outside of your household? | Name:_______________________ |
|  |  |  |
| TG013 |  | Location:____________________ |
|  |  |  |
| TG014 |  | Phone number:_______________ |
|  |  |  |
| TG015 | What is this person's relationship to you? | (M) Mother………………………………..1 (F) Father……………………………………2 (B) Brother…………………………………3 (Z) Sister…………………………………….4 (S) Son………………………………………..5 (D) Daughter………………………………6  (T) HH head………………………………..7 (H) Husband……………………………….8 (W) Wife…………………………………….9 (R) Related indirectly………………..10 (U) Unrelated……………………………11 |
|  |  |  |
| TG016 | What is the name, location (address and description), and mobile phone number of another person outside of your household? | Name:_______________________ |
|  |  |  |
| TG017 |  | Location:____________________ |
|  |  |  |
| TG018 |  | Phone number:_______________ |
|  |  |  |
| TG019 | What is this person's relationship to you? | (M) Mother………………………………..1 (F) Father……………………………………2 (B) Brother…………………………………3 (Z) Sister…………………………………….4 (S) Son………………………………………..5 (D) Daughter………………………………6  (T) HH head………………………………..7 (H) Husband……………………………….8 (W) Wife…………………………………….9 (R) Related indirectly………………..10 (U) Unrelated……………………………11 |
|  |  |  |
| TG020 | Other than this place of residence (house), where else does the household head frequently stay? Is there a phone number and contact person there? | Other residence_______________ |
|  |  |  |
| TG021 |  | Phone number:_______________ |
|  |  |  |
| TG022 |  | Contact person there:__________ |
|  |  |  |
| TG023 | Do you have an email address that you check regularly? | YES................................................1  NO ................................................2 |
|  |  |  |
| TG024 | Can you please give me your email address? | ____________________________ |

**Section: Biomarkers**

| BS001 | We will now be collecting your blood pressure using a device that is placed on the arm. I would like to place the blood pressure device on your arm now. We will take three measurements, waiting a few minutes in between each one. During the measurement the cuff will inflate and you will feel pressure on your arm. |  |
| --- | --- | --- |
|  |  |  |
| BS002_left | Have you had any recent surgeries or injuries to your left arm that will prevent me from wrapping the cuff around your left upper arm? | YES................................................1  NO ................................................2 |
|  |  |  |
| BS002_left_specify | [IWER: PLEASE DESCRIBE THE PREVENTING CONDITIONS IN THE LEFT ARM. CONDITIONS INCLUDE: OPEN SORES, WOUNDS, GAUZE DRESSINGS OR RASHES, PARTICIPANT HAS NO LEFT ARM.] |  |
|  |  |  |
| BS002_right | Have you had any recent surgeries or injuries to your right arm that will prevent me from wrapping the cuff around your right upper arm? | YES................................................1  NO ................................................2 |
|  |  |  |
| BS002_right_specify | [IWER: PLEASE DESCRIBE THE PREVENTING CONDITIONS IN THE LEFT ARM. CONDITIONS INCLUDE: OPEN SORES, WOUNDS, GAUZE DRESSINGS OR RASHES, PARTICIPANT HAS NO RIGHT ARM.] |  |
|  |  |  |
| BS003 | In the past 30 minutes, have you eaten any food, smoked a cigarette, or exercised? | YES................................................1  NO ................................................2 |
|  |  |  |
| BS005 | Now I will take the first of three blood pressure readings. While I am measuring your blood pressure, it is very important that you remain still, sitting up straight with your arms relaxed, your feet flat on the ground, and that we not have any conversation. [IWER: INTERVIEWER: ENSURE THE SETTING IS APPROPRIATE AND RESPONDENT IS READY FOR MEASUREMENT (SUFFICIENTLY QUIET, CALM, RELAXED, SITTING STRAIGHT WITH FEET FLAT ON GROUND) FOR BLOOD PRESSURE MEASUREMENT] [IWER: IT IS IMPORTANT AT THIS POINT THAT THE PARTICIPANT REMAINS STILL AND DOES NOT SPEAK. WHEN THE MEASUREMENT FINISHES THE CUFF WILL DEFLATE AND THE MEASUREMENT RESULTS (BLOOD PRESSURE VALUES, PULSE RATE, DATE AND TIME) ARE THEN DISPLAYED. IF YOU NEED TO STOP THE MEASUREMENT, PUSH THE START/STOP BUTTON TO TURN OFF THE POWER.] |  |
|  |  |  |
| BS008 | [IWER: YOU HAVE PLACED MONITOR ON ^BS007 ARM. HAVE YOU CHANGED THE ARM SELECTION SETTING TO INDICATE THE ^BS007 ARM?] |  |
|  |  |  |
| BS009 | [IWER: IT IS IMPORTANT AT THIS POINT THAT THE PARTICIPANT REMAINS STILL AND DOES NOT SPEAK.  WHEN THE MEASUREMENT FINISHES THE CUFF WILL DEFLATE AND THE MEASUREMENT RESULTS (BLOOD PRESSURE VALUES, PULSE RATE, DATE AND TIME) ARE THEN DISPLAYED.  IF YOU NEED TO STOP THE MEASUREMENT, PUSH THE START/STOP BUTTON TO TURN OFF THE POWER.] |  |
|  |  |  |
| BS010 | [IWER: INDICATE OUTCOME OF BLOOD PRESSURE READING] | Reading obtained……………………….1  Missed reading (equipment failure, interruption, other problem……………………………………..2  Refused………………………………………3 |
|  |  |  |
| BS011 | [IWER: REASON NO BLOOD PRESSURE MEASUREMENT OBTAINED? MARK ALL THAT APPLY.] | Respondent unable/unwilling to understand and follow instructions………………………………..1  Respondent refuses to be measured…………………………………..2  Withered arms, injury, recent surgery, dressing, rash (on both arms)………………………………………….3  Equipment failure………………………4  Other………………………………………….5 |
|  |  |  |
| BS012 | [IWER: ENTER BLOOD PRESSURE AND PULSE READING] ENTER SYSTOLIC READING: | ___________________SYS/mmHg |
|  |  |  |
| BS013 | ENTER DIASTOLIC READING: | ___________________DIA/mmHg |
|  |  |  |
| BS014 | ENTER PULSE READING: | ___________________PULSE/min |
|  |  |  |
| BS015_intro | [IWER: RE-ENTER BLOOD PRESSURE AND PULSE READING ]  RE-ENTER SYSTOLIC READING  ____________________SYS/mmHg  RE-ENTER DIASTOLIC READING: ____________________DIA/mmHg  RE-ENTER PULSE READING ____________________PULSE/min |  |
|  |  |  |
| BS015 | [IWER: RE-ENTER BLOOD PRESSURE AND PULSE READING] RE-ENTER SYSTOLIC READING: | ___________________SYS/mmHg |
|  |  |  |
| BS015_delay | [IWER: WAIT TWO MINUTES UNTIL NEXT MEASUREMENT. USE THIS TIME TO RETRIEVE AND PREPARE THE REST OF YOUR EQUIPMENT SUCH AS THE SCALE, TAPE MEASURE, WALKING COURSE, AND GRIP STRENGTH. ENSURE THE PARTICIPANT REMAINS CALM AND RELAXED DURING THESE TWO MINUTES. ] |  |
|  |  |  |
| BS016 | RE-ENTER DIASTOLIC READING: |  |
|  |  |  |
| BS017 | RE-ENTER PULSE READING: |  |
|  |  |  |
| BS018_form4 | [IWER: THE BLOOD SPOT SAMPLE COLLECTION (FORM 4). Make sure that: it has been SIGNED and DATED by the field worker, it has been SIGNED and DATED by the respondent, a copy has been LEFT with the respondent, you KEPT a copy for our records] | Yes, participant agreed to blood spot collection……………………………1  No, participant refused blood spot collection……………………………………2 |
|  |  |  |
| BS019_intro | [IWER: PLACE BARCODE STICKER ON CARD. REMEMBER TO RECORD YOUR NAME AND DATE ON CARD AS WELL. HOLD THE BAR CODE IN FRONT OF THE WEBCAM AT THE TOP OF THE SCREEN TO RECORD THE UNIQUE BARCODE NUMBER FOR DRIED BLOOD SPOT CARD TO BE USED WITH SUBJECT.] |  |
|  |  |  |
| BS019 | [IWER: ENTER UNIQUE ID CODE LISTED BELOW BARCODE, eg: VZHOT] [IWER: THIS IS JUST AN EXAMPLE. ENTER THE NUMBER FROM THE STICKER YOU USED!!!] |  |
|  |  |  |
| BS019_read | Please hold the barcode in front of the webcam until a clear picture appears. |  |
|  |  |  |
| BS020 | [IWER: INDICATE IF DBS CARD NUMBER WAS ENTERED WITH BAR CODE READER OR MANUALLY.] |  |
|  |  |  |
| BS021 | [IWER: RE-ENTER UNIQUE ID CODE LISTED BELOW BARCODE] |  |
|  |  |  |
| BS021_check | [IWER: ID CODES DO NOT MATCH. GO BACK TO CHECK THE CODES YOU ENTERED] |  |
|  |  |  |
| BS022 | [IWER: PLEASE PROCEED WITH BLOOD SPOT COLLECTION. REFER TO "BLOODSPOT COLLECTION CHECKLIST" IF NEEDED. DID YOU COLLECT AND LOAD A BLOOD SPECIMEN IN HEMOCUE 201+ ANALYZER (HEMOGBLOIN ANALYZER)] | YES................................................1  NO ................................................2 |
|  |  |  |
| BS022_notspecify | [IWER: WHY DID YOU NOT COLLECT AND LOAD A BLOOD SPECIMEN IN HEMOCUE 201+ ANALYZER (HEMOGBLOIN ANALYZER)?] | ___________________________ |
|  |  |  |
| BS023 | [IWER: DID YOU COLLECT AND LOAD A BLOOD SPECIMEN IN CareSens ANALYZER (GLUCOSE ANALYZER)?] | YES................................................1  NO ................................................2 |
|  |  |  |
| BS023_notspecify | [IWER: WHY DID YOU NOT COLLECT AND LOAD A BLOOD SPECIMEN IN CareSens ANALYZER (GLUCOSE ANALYZER)?] | ___________________________ |
|  |  |  |
| BS024 | [IWER: DID YOU COLLECT AND LOAD A BLOOD SPECIMEN IN CARDIOCHECK ANALYZER (LIPID PANEL ANALYZER)?] | YES................................................1  NO ................................................2 |
|  |  |  |
| BS024_notspecify | [IWER: WHY DID YOU NOT COLLECT AND LOAD A BLOOD SPECIMEN IN CARDIOCHECK ANALYZER (LIPID PANEL ANALYZER)?] | ______________________[0 to 5] |
|  |  |  |
| BS025 | [IWER: HOW MANY BLOOD SPOTS DID YOU COLLECT THAT ARE >80% FILLED?] | ____________________________ |
|  |  |  |
| BS026  *[use c_bs_hemoglobin]* | [IWER: RECORD HEMOGLOBIN (HEMOCUE 201+ ANALYZER) READING BELOW.  HEMOGLOBIN (HEMOCUE 201+ ANALYZER) READING] | ____________________________ |
|  |  |  |
| BS026_check  *[use c_bs_hemoglobin]* | [IWER: CHECK HEMOGLOBIN (HEMOCUE 201+ ANALYZER) ENTRIES] | ____________________________ |
|  |  |  |
| BS027  *[use c_bs_hemoglobin]* | RE-ENTER HEMOGLOBIN (HEMOCUE 201+ ANALYZER) READING: | ____________________________ |
|  |  |  |
| BS028  *[use c_bs_glucose]* | RECORD GLUCOSE (CareSens ANALYZER) READING BELOW | ____________________________ |
|  |  |  |
| BS028_check  *[use c_bs_glucose]* | [IWER: CHECK GLUCOSE (CareSens ANALYZER) ENTRIES] | ____________________________ |
|  |  |  |
| BS029  *[use c_bs_glucose]* | RE-ENTER RECORD GLUCOSE (CareSens ANALYZER) READING BELOW | ____________________________ |
|  |  |  |
| BS030  *[use c_bs_chol]* | RECORD CHOLESTEROL (CARDIOCHECK ANALYZER) READING BELOW | ____________________________ |
|  |  |  |
| BS030_check  *[use c_bs_chol]* | [IWER: CHECK CHOLESTEROL (CARDIOCHECK ANALYZER) READINGS] | ____________________________ |
|  |  |  |
| BS031  *[use c_bs_hdl]* | RECORD HDL CHOLESTEROL (CARDIOCHECK ANALYZER) READING BELOW | ____________________________ |
|  |  |  |
| BS032  *[use c_bs_ldl]* | RECORD LDL CHOLESTEROL (CARDIOCHECK ANALYZER) READING BELOW | ____________________________ |
|  |  |  |
| BS033  *[use c_bs_trig]* | RECORD TRIGLYCERIDES CHOLESTEROL (CARDIOCHECK ANALYZER) READING BELOW | ____________________________ |
|  |  |  |
| BS034  *[use c_bs_chol]* | RE-ENTER CHOLESTEROL (CARDIOCHECK ANALYZER) READING BELOW | ____________________________ |
|  |  |  |
| BS035  *[use c_bs_hdl]* | RE-ENTER HDL CHOLESTEROL (CARDIOCHECK ANALYZER) READING BELOW | ____________________________ |
|  |  |  |

| BS036  *[use c_bs_ldl]* | RE-ENTER LDL CHOLESTEROL (CARDIOCHECK ANALYZER) READING BELOW | ____________________________ |
| --- | --- | --- |
|  |  |  |
| BS037  *[use c_bs_trig]* | RE-ENTER TRIGLYCERIDES CHOLESTEROL (CARDIOCHECK ANALYZER) READING BELOW | ____________________________ |
|  |  |  |
| BS038 | [IWER: PREPARE THE FEEDBACK CARD BY ENTERING THE BLOOD RESULTS DISPLAYED BELOW AND PROVIDE TO SUBJECT ON (DATE): HEMOGLOBIN READING:  GLUCOSE READING:  CHOLESTEROL READING:  HDL CHOLESTEROL READING:  LDL CHOLESTEROL READING: TRIGLYCERIDES READING:  BMI:  BP:  sys:  dia:  pulse: |  |

| BS039 | [IWER: SPECIFY THE REASON(S) RESPONDENT DID NOT PROVIDE BLOOD SPOTS. SELECT ALL THAT APPLY] | Insufficient blood from finger stick……………………………………………1  Respondent felt faint…………………2  Equipment malfunction……………..3  FI did not have materials……………4  Request too invasive………………….5  Respondent does not give biologic samples……………………………………..6  Religious reasons……………………….7  Respondent concerned about confidentiality……………………………8  Respondent cannot due to illness/disability………………………….9  Other……………………………………….11 |
| --- | --- | --- |
|  |  |  |

| BS040 | Now I would like to measure your height and weight. [IWER: PLACE THE SCALE ON A FIRM SURFACE (AN UNCARPETED FLOOR AND NEAR A WALL OR DOORFRAME FOR SUPPORT). DO NOT USE A CARPETED FLOOR. IN HOMES A KITCHEN OR BATHROOM FLOOR WILL WORK BEST. ASK THE RESPONDENT EMPTY THEIR POCKETS AND TO REMOVE SHOES, HAT, HAIR ORNAMENTS, AND ANY HEAVY OUTER GARMENTS. ENSURE THE SCALE IS MEASURING IN KG AND CM. ASK THE PARTICPANT TO STEP ON THE SCALE WITH THEIR FEET SHOULD WIDTH APART, LOOKING STRAIGHT AHEAD WITH THEIR HEAD LEVEL. HAVE THE PARTICIPANT TAKE A BREATH AND HOLD IT TO ENSURE THEY ARE STANDING TALL. MAKE SURE THE HEIGHT SENSOR REMAINS LEVEL (HORIZONTAL TO THE FLOOR) AND CENTERED ON THE PARTICIPANTS HEAD. PUSH AND HOLD THE BUTTON ON THE SENSOR TO MEASURE HEIGHT. SUCCESSFULLY ABLE TO MEASURE RESPONDENT'S HEIGHT?] |  |
| --- | --- | --- |
|  |  |  |
| BS041  *[use c_bs_height]* | [IWER: ENTER RESPONDENT'S HEIGHT IN CENTIMETERS] | __________________Centimeters |
|  |  |  |
| BS041_check  *[use c_bs_height]* | [IWER: ENTER RESPONDENT'S HEIGHT IN CENTIMETERS] | __________________Centimeters |
|  |  |  |
| BS042  *[use c_bs_height]* | [IWER: RE-ENTER RESPONDENT'S HEIGHT IN CENTIMETERS] | __________________Centimeters |
|  |  |  |
| BS043 | [IWER: DOES ANYTHING IMPACT THE ACCURACY OF THE HEIGHT MEASUREMENT (E.G. PARTICIPANT WEARING SHOES, NOT ABLE TO FLATTEN HAIR TO MEASURE, ETC.)?] | YES….............................................1  NO ….............................................2 |
|  |  |  |
| BS044 | [IWER: SELECT ALL REASONS WHY UNABLE TO ACCURATELY MEASURE HEIGHT. MARK ALL THAT APPLY] | Respondent unable/unwilling to understand and follow instructions………………………………..1  Respondent refuses to be measured…………………………………..2  Respondent unable to stand unassisted………………………………….3  Unable to position respondent according to protocol…………………4  Unsuitable surface……………………..5  Equipment failure………………………6  Other………………………………………….7 |
|  |  |  |
| BS045 | [IWER: SUCCESSFULLY ABLE TO MEASURE RESPONDENT'S WEIGHT?] | YES................................................1  NO ................................................2 |
|  |  |  |
| BS046  *[use c_bs_weight]* | [IWER: ENTER RESPONDENT'S WEIGHT IN KILOGRAMS] | ____________________Kilograms |
|  |  |  |
| BS046_check  *[use c_bs_weight]* | [IWER: ENTER RESPONDENT'S WEIGHT IN KILOGRAMS] | ____________________Kilograms |
|  |  |  |
| BS047  *[use c_bs_weight]* | [IWER: RE-ENTER RESPONDENT'S WEIGHT IN KILOGRAMS] | ____________________Kilograms |
|  |  |  |
| BS048 | [IWER: DID ANYTHING IMPACT YOUR ABILITY TO COLLECT AN ACCURATE WEIGHT MEASUREMENT?] | YES................................................1  NO ................................................2 |
|  |  |  |
| BS049 | [IWER: SELECT ALL REASONS WHY UNABLE TO ACCURATELY MEASURE RESPONDENT'S WEIGHT. MARK ALL THAT APPLY] | Respondent unable/unwilling to understand and follow instructions………………………………..1  Respondent refuses to be weighed…………………………………..2  Respondent unable to stand unassisted………………………………….3  Unsuitable surface for setting up the scale………………….…………………4  Scale insufficient for respondent weight………………………………………..5  Equipment failure………………………6  Other………………………………………….7 |
|  |  |  |
| BS051  *[use c_bs_hrslastate]* | At what time did you last eat or drink (besides water)? | ____________________________ |
|  |  |  |
| BS052 | Code scanned: |  |
|  |  |  |
| **Created Variables:** | **Biomarkers Section** |  |
| *Variable* | *Label* | *Source* |
|  |  |  |
| c_bs_anemia | Anemia | If c_hemoglobin not flagged:  1. Men  Normal >12.9g/dl  Mild anemia ≤12.9g/dl AND ≥11g/dl  Moderate anemia <11g/dl AND ≥8g/dl  Severe anemia <8g/dl.  2. Women  Normal >11.9g/dl  Mild anemia ≤11.9g/dl AND ≥11g/dl  Moderate anemia <11g/dl AND ≥8g/dl  Severe anemia <8g/dl |
| c_bs_bmi | BMI | BMI calculated from cleaned height and cleaned weight |
|  |  |  |
| c_bs_bmicat | BMI category | BMI category derived from c_bs_bmi (Underweight: <18.5, Normal: 18.5 to < 25, Overweight: 25 to <30, Obese: >= 30) |
|  |  |  |
| c_bs_chol | Point of care cholesterol measurement (mmol/L) | Cleaned cholesterol reading (mmol/L): Measures taken from bs030, bs034, and comments data. |
|  |  |  |
| c_bs_fasting8hr | Was fasting for at least 8 hours at start of blood collection | Participant was fasting for at least 8 hours at time of blood collection. Determined from c_bs_hrslastate. |
|  |  |  |
| c_bs_glucose | Point of care glucose measurement (mmol/L) | Cleaned glucose reading (mmol/L): Measures taken from bs028, bs028_check, bs029, and comments data. |
|  |  |  |
| c_bs_hdl | Point of care HDL measurement (mmol/L) | Cleaned HDL (mmol/L): Measures taken from bs031, bs035, and comments data. |
|  |  |  |
| c_bs_height | Cleaned height (cm) | Cleaned height (cm): Measures taken from bs041, bs042, comments data, lab data, and re-visit data. |
|  |  |  |
| c_bs_height_origin | Origin of height value | Height measured during home interview, lab interview, or re-visit for height/weight collection |
|  |  |  |
| c_bs_hemoglobin | Point of care hemoglobin measurement (g/dL) | Cleaned hemoglobin reading (g/dL): Measures taken from bs026, bs026_check, bs027, and comments data. |
|  |  |  |
| c_bs_highchol | High total cholesterol | Elevated total cholesterol (≥6.21 mmol/L), if c_chol not flagged |
|  |  |  |
| c_bs_highldl | High LDL | Elevated LDL (>4.1 mmol/L), if c_ldl not flagged |
|  |  |  |
| c_bs_hightrig | High triglycerides | Elevated triglycerides (>2.25 mmol/L), if c_trig not flagged |
|  |  |  |
| c_bs_hrslastate | Hours since last ate at start of blood collection | Hours since last ate at the start of blood collection. Calculated from comparing time since reported last ate (bs051) to time stamp of start of blood collection. Assumes that if the response to bs051 was later than timestamp of blood collection that the respondent last ate the previous day. |
|  |  |  |
| c_bs_ldl | Point of care LDL measurement (mmol/L) | Calculated LDL (mmol/L): Measures taken from bs032, bs036, and comments data. |
|  |  |  |
| c_bs_lowhdl | Low HDL | Low HDL (<1.19 mmol/L), if c_hdl not flagged |
|  |  |  |
| c_bs_mean_dia | Diastolic blood pressure (mmHg) | Mean diastolic blood pressure (mmHg): Mean of second 2 diastolic blood pressure readings; if only 2 readings are available second reading is used; if only 1 reading is available first reading is used. |
|  |  |  |
| c_bs_mean_pulse | Pulse (BPM) | Mean pulse (BPM): Mean of second 2 pulse readings; if only 2 readings are available second reading is used; if only 1 reading is available first reading is used. |
|  |  |  |
| c_bs_mean_sys | Systolic blood pressure (mmHg) | Mean systolic blood pressure (mmHg): Mean of second 2 systolic blood pressure readings; if only 2 readings are available second reading is used; if only 1 reading is available first reading is used. |
|  |  |  |
| c_bs_trig | Point of care triglycerides measurement (mmol/L) | Cleaned triglycerides (mmol/L): Measures taken from bs033, bs037, and comments data. |
|  |  |  |
| c_bs_weight | Cleaned weight (kg) | Cleaned weight (kg): Measures taken from bs046, bs046_check, bs047, comments data, lab data, and re-visit data. |
|  |  |  |
| c_bs_weight_origin | Origin of weight value | Weight measured during home interview, lab interview, or re-visit for height/weight collection |
|  |  |  |
| fl_c_bs_bmi | Flag for c_bs_bmi | Either height or weight was flagged, or BMI <11, or BMI >60 |
|  |  |  |
| fl_c_bs_chol | Flag for c_bs_chol | Cleaned cholesterol out of range (<1mmol/L) |
|  |  |  |
| fl_c_bs_glucose | Flag for c_bs_glucose | Cleaned glucose out of test range (<1.1mmol/L or >33.3 mmol/L) |
|  |  |  |
| fl_c_bs_hdl | Flag for c_bs_hdl | Cleaned HDL out of range (<0.39mmol/L or >2.59mmol/L) |
|  |  |  |
| fl_c_bs_height | Flag for c_bs_height | Cleaned height measurement out of range (<125cm or >220cm) |
|  |  |  |
| fl_c_bs_hemoglobin | Flag for c_bs_hemoglobin | Cleaned hemoglobin out of range (<3 g/dL or >30g/dL) |
|  |  |  |
| fl_c_bs_ldl | Flag for c_bs_ldl | Cleaned calculated LDL out of range (>10 mmol/L); or triglycerides > 4.52 mmol/L (calculated LDL will be artificially low or non-reportable if the triglycerides are abnormally high (ie, >400 mg/dL (4.52mmol/L))). |
|  |  |  |
| fl_c_bs_trig | Flag for c_bs_trig | Cleaned triglycerides out of range (<0.56mmol/L or >5.65mmol/L) |
|  |  |  |
| fl_c_bs_weight | Flag for c_bs_weight | Cleaned weight measurement out of range (<30kg or >160kg) |
|  |  |  |
|  |  |  |

**Other Variables**

| **Other: Respondent And Interview Details** | | |
| --- | --- | --- |
| *Variable* | *Label* | *Source* |
|  |  |  |
| c_int_month | Interview month |  |
|  |  |  |
| c_int_year | Interview year |  |
|  |  |  |
| c_int_time | Begin time of interview |  |
|  |  |  |
| c_week | Week of interview |  |
|  |  |  |
|  |  |  |
| rproxy | Proxy interview | Indicates if a proxy respondent was surveyed |
|  |  |  |
| urid | Interviewer ID | Anonymous ID for interviewer |
|  |  |  |
| village_anon | Village ID | Anonymous ID for village of respondent |
|  |  |  |
| rage | Respondent age | Age on July 31, 2014 at time of sample selection (preloaded from census) |
|  |  |  |
| rsex | Respondent sex | Preloaded from census |
|  |  |  |
| c_agegrp | Age group | Age group from rage |
|  |  |  |
| rbirthyear | Respondent birth year | Preloaded from census |
|  |  |  |
| c_hhnum | Number of individuals in household | Determined from household roster of permanent members and preloaded value from census |
|  |  |  |
| c_hhsize | Categorical number of individuals living in household | Categorization of c_hhnum |
|  |  |  |
| **Other: Created Variables** | | |
| *Variable* | *Label* | *Source* |
|  |  |  |
| c_hh_assetindex | Asset index score | Created from census and asset ownership questions |
|  |  |  |
| fl_c_hh_assetindex | Flag for household asset index | Flag indicates if imputed values were used |
|  |  |  |
| c_wealthindex | Wealth asset index | Household quintiles from c_hh_assetindex score |
|  |  |  |
